# Fast track to environmentally adapted rhizobia for growing soybean at northern latitudes using citizen science

**DOI:** 10.1093/ismejo/wraf152

**Published:** 2025-08-06

**Authors:** Sonia García Méndez, Stien Mertens, Arne Temmerman, Helena Van den Eynde, Margo Vermeersch, Lena Vlaminck, Olivier Berteloot, Judith Van Dingenen, Alexander Clarysse, Annick De Keyser, Serge Beullens, Ilse de Baenst, Niranjana Roy, Quinten De Paepe, Jan Michiels, Isabel Roldan-Ruiz, Joke Pannecoucque, Anne Willems, Steven Maere, Sofie Goormachtig

**Affiliations:** Department of Plant Biotechnology and Bioinformatics, Ghent University, Ghent 9052, Belgium; Center for Plant Systems Biology, VIB, Ghent 9052, Belgium; Department of Plant Biotechnology and Bioinformatics, Ghent University, Ghent 9052, Belgium; Center for Plant Systems Biology, VIB, Ghent 9052, Belgium; Department of Plant Biotechnology and Bioinformatics, Ghent University, Ghent 9052, Belgium; Center for Plant Systems Biology, VIB, Ghent 9052, Belgium; Department of Plant Biotechnology and Bioinformatics, Ghent University, Ghent 9052, Belgium; Center for Plant Systems Biology, VIB, Ghent 9052, Belgium; Department of Biochemistry and Microbiology, Ghent University, Ghent 9000, Belgium; Plant Sciences Unit, Flanders Research Institute for Agriculture, Fisheries and Food (ILVO), Merelbeke 9820, Belgium; Department of Plant Biotechnology and Bioinformatics, Ghent University, Ghent 9052, Belgium; Center for Plant Systems Biology, VIB, Ghent 9052, Belgium; Department of Plant Biotechnology and Bioinformatics, Ghent University, Ghent 9052, Belgium; Center for Plant Systems Biology, VIB, Ghent 9052, Belgium; Department of Plant Biotechnology and Bioinformatics, Ghent University, Ghent 9052, Belgium; Center for Plant Systems Biology, VIB, Ghent 9052, Belgium; Department of Plant Biotechnology and Bioinformatics, Ghent University, Ghent 9052, Belgium; Center for Plant Systems Biology, VIB, Ghent 9052, Belgium; Department of Plant Biotechnology and Bioinformatics, Ghent University, Ghent 9052, Belgium; Center for Plant Systems Biology, VIB, Ghent 9052, Belgium; Center for Microbiology, VIB, Leuven, 3001, Belgium; Center for Microbial and Plant Genetics, KU Leuven, Leuven 3001, Belgium; Department of Biochemistry and Microbiology, Ghent University, Ghent 9000, Belgium; Department of Biochemistry and Microbiology, Ghent University, Ghent 9000, Belgium; Department of Plant Biotechnology and Bioinformatics, Ghent University, Ghent 9052, Belgium; Center for Plant Systems Biology, VIB, Ghent 9052, Belgium; Department of Biochemistry and Microbiology, Ghent University, Ghent 9000, Belgium; Center for Microbiology, VIB, Leuven, 3001, Belgium; Center for Microbial and Plant Genetics, KU Leuven, Leuven 3001, Belgium; Plant Sciences Unit, Flanders Research Institute for Agriculture, Fisheries and Food (ILVO), Merelbeke 9820, Belgium; Plant Sciences Unit, Flanders Research Institute for Agriculture, Fisheries and Food (ILVO), Merelbeke 9820, Belgium; Department of Biochemistry and Microbiology, Ghent University, Ghent 9000, Belgium; Department of Plant Biotechnology and Bioinformatics, Ghent University, Ghent 9052, Belgium; Center for Plant Systems Biology, VIB, Ghent 9052, Belgium; Department of Plant Biotechnology and Bioinformatics, Ghent University, Ghent 9052, Belgium; Center for Plant Systems Biology, VIB, Ghent 9052, Belgium

**Keywords:** soybean, rhizobia, nodulation, root nodule, biological nitrogen fixation, microbiome, soil, local rhizobial strains, Bradyrhizobium, citizen science

## Abstract

Soybean serves as a crucial source of plant-based protein for human diets. Recently, there is a growing incentive to extend the range of this crop to more northern latitudes, in order to enable profitable soybean production in Europe. To reach economic yields, soybean requires inoculation with symbiotic, diazotrophic rhizobial bacteria. However, the performance of commercial inocula is often variable under local conditions. Here, we present the citizen science project “Soy in 1,000 Gardens”, a large-scale trapping experiment for isolating local soybean-nodulating rhizobia in Flanders, Belgium. We identified two locally isolated *Bradyrhizobium* strains performing at least as well as commercial strain *B. diazoefficiens* G49 in local field trials. Additionally, we found that nutrient content, microbial alpha diversity, and the presence of arbuscular mycorrhizal fungi in the soil were correlated with nodulation. Finally, we report a correlation between low bacterial alpha diversity and red nodule interior, and identified *Tardiphaga* as a dominant colonizer of red nodules.

## Introduction

Soybean (*Glycine max*), the world’s most extensively farmed legume, serves as a crucial source of plant-based protein for both human diets and animal feed [1]. Currently, the leading producers of soybean are the USA, Argentina, and Brazil [2], but there is growing incentive to extend the range of the crop to more northern regions, including North-western Europe. This call is largely based on the prediction that local production, of soy in particular and of food in general, could significantly reduce greenhouse gas emissions [3, 4]. Because there is great potential to lower greenhouse gas emissions even further by promoting a switch from a meat-based to a plant-based human diet, soybeans continue to receive much attention as an alternative to meat derived protein in the human diet [3]. Especially in Europe, providing a locally grown, non-GMO alternative to the soy grown in the Americas could greatly increase the willingness to adopt soy in the daily diet [5].

Like many legumes, soy can be grown largely independently of expensive and environmentally taxing nitrogen fertilizer, thanks to a symbiotic relationship with diazotrophic rhizobial bacteria [6–8]. Housed inside specialized root organs called nodules, these symbiotic bacteria provide the plant with accessible nitrogen through the process of biological nitrogen fixation (BNF). Besides providing nitrogen for the protein-rich seeds, BNF leads to a net flux of nitrogen from the atmosphere to the plant, meaning that non-harvested parts of soy plants can act as a natural nitrogen fertilizer in crop rotation systems [9–11].

In regions outside the original range of the crop, soybean seeds have to be inoculated with rhizobia to reach economic yields. For this, elite strains are available in commercial inoculants, which have been routinely used in the major soy-producing areas, and have been successful in several European countries, including Switzerland, Austria, Germany, Russia, Scotland, and Belgium [9, 10, 12–15]. However, despite some success, the soybean yields obtained at northern latitudes with commercial inoculants often appear to be either insufficient or variable, likely because these inoculants are not fully adapted to the local climate [5, 9, 16].

A strategy to overcome this is to develop inocula from locally isolated rhizobial strains. It is debated whether or not indigenous soybean-nodulating rhizobia exist outside the original range of the crop, even though nodulation has been reported in non-inoculated soils [15, 17, 18]. Rather than true indigenous rhizobia, this is often attributed to commercial inoculants that have become naturalized, which entails the survival of an inoculant in the soil and its subsequent adaptation to the local climate. Conversely, it might be possible that local wild legumes are colonized by rhizobia that are also compatible with soy, thus maintaining a population of soybean-nodulating rhizobia in regional soils. Indeed, several *Bradyrhizobium* species were found in root nodules of legumes native to Europe, and these might be suitable candidates for the nodulation of soybean grown at northern latitudes [19, 20]. Whether or not they were truly indigenous or not, locally isolated rhizobia have, on occasion, been shown to outperform commercial inoculants, most probably owing to their adaptation to the local climate [21–23].

Trapping is a straightforward way to isolate local rhizobia from nodules of soy grown in non-inoculated soil. This strategy has been applied successfully to isolate local soybean-nodulating rhizobia in several African and South American countries, and recently also in Germany [21, 24–27]. Earlier, we conducted a small-scale study leading to the isolation of several soybean-nodulating strains from Flemish soil [18]. However, the efficacy of the local strains found in these studies was not yet clearly demonstrated.

In this “Soy in 1,000 Gardens” project, based on our previous results [18], we enlarged the trapping experiment, hypothesising that indeed there are local rhizobia capable of nodulating soybean, but that these are rare and present in too low abundances in uninoculated soil to efficiently nodulate soybean. Therefore, we set up a large-scale trapping experiment with the help of citizen scientists for the identification of local soybean-nodulating rhizobia throughout the region of Flanders, Belgium. Citizen science allows the collection of material and data on a large scale, helping to counter the expected rarity of efficient soybean-nodulating strains [28]. With the help of citizens, soybean was grown in 1,216 gardens, which are ideal for finding local rhizobia because garden soil is generally nitrogen-poor and thus conducive to nodulation [29]. Moreover, the collaboration with citizen scientists from around Flanders allowed us to monitor the willingness of consumers to adopt soy in a more plant-based diet, and to raise awareness in an effort to increase this willingness.

Our goal was fourfold: (i) to study the existence of local rhizobial strains capable of nodulating soybean, (ii) to examine whether locally adapted rhizobial strains can outperform commercial inoculants, (iii) to gain detailed insights into the influence of microbial and soil parameters on soybean nodulation in Flanders, and (iv) to raise public awareness about the benefits of legumes and microbes, and study the drivers for participation in citizen science projects. Here, we describe in detail the soybean garden trapping experiment, the microbial and soil parameters influencing nodulation, the bacterial isolates obtained from these nodules, and the competitiveness of these strains in the field. We refer to earlier publications for a detailed description of the project workflow [28, 30], as well as novel insights into the drivers for citizens’ engagement and participant retention [31, 32].

## Materials and methods

### Participant recruitment and selection

Participants were recruited via “Mijn Tuinlab”, an interactive platform where citizen science projects linked to gardens are gathered (https://mijntuinlab.be) [30]. The selection of participants from the pool of interested citizens was based on garden location, whether their garden was chemically/organically fertilized (25% of selected gardens) or not (75% of selected gardens), and demographic characteristics (e.g. age, gender, and education level). A total of 1,200 candidates were selected out of 5,335 applicants. All candidates received a unique ID number and were asked to create an account on the project website to confirm their participation and to ensure they had the digital skills required for online data submission throughout the project. Notably, though 1,200 gardens were available, some analyses have been done on fewer gardens due to technical and organizational reasons.

### Soil samples

Soil samples were taken in April 2021, when the participation packages were delivered, and the garden grid was prepared. Soil samples of the top 30 cm were collected in each garden by mixing 20 to 30 auger samples that were randomly taken throughout the plot. Subsamples were then stored for physicochemical soil characteristics (PSC) analysis (4°C), phospholipid-derived fatty acids (PLFA) analysis (−20°C), and 16S rRNA and internal transcribed spacer (ITS) microbial profiling (−70°C).

### Analysis of physicochemical soil characteristics

The PSC analysis of 1,138 samples was performed by the Soil Service of Belgium using a standardised soil analysis protocol (KEMA analysis) including an ammonium lactate extraction [33]. During the PSC data processing, the measurements that had a value below the detection limit were handled with an often-used substitution-based imputation method, namely to set the value to half the detection limit [34]. This approach could only be applied to variables that had a known detection limit, which was not the case for ammonium. Consequently, ammonium values that fell below the detection limit were set to 0.

### PLFA soil analysis

PLFA isolation and analysis were performed on 1,151 samples with a previously described protocol with minor modifications [35]. Details are provided in the Supplementary Information.

### Bulk soil data exploration

All soil data analyses were carried out using R, versions 4.0.3 and 4.1.3 [36]. The datasets analysed included the participant/garden level PSC (*n* = 1093), PLFA (*n* = 1151), ITS microbiome (*n* = 1079), and 16S rRNA microbiome (*n* = 1152) data, as well as information on previous fertilization practices of the citizen scientists, the presence of other legumes in their gardens, and the presence of nodules on the harvested soybean roots (data available in a zenodo archive: https://doi.org/10.5281/zenodo.12755613). Initial exploration of the 16S rRNA and ITS data was performed on amplicon sequence variant (ASV) level, whereas for the correlation analyses and models (see below) only genus-level data was used. Data exploration included a principal component analysis (PCA) to evaluate batch effects, spatial autocorrelation, and the presence of potential confounding factors and outliers (prcomp function, stats R package [36]). Further details are provided in the Supplementary Information.

### Nodule presence prediction modeling

Models were developed to investigate whether bulk soil data can be used to predict the presence of nodules in a given garden and, if so, which soil factors potentially influence nodule presence. Soil PSC data, corrected PLFA data, ITS and 16S rRNA genus-level data, data on the previous fertilization practices of the citizen scientists and the presence of other legumes in their gardens (data available in a zenodo archive: https://doi.org/10.5281/zenodo.12755613), and the Shannon Index were used to train single- and multi-variable models. Different modelling approaches were evaluated, including auto-logistic regression (auto-log), elastic net logistic regression (eln-log), random forest classification (RF), and partial least squares auto-logistic regression (pls-log). Because the PCA analyses and variable maps indicated that a spatial structure was present in the data (Figs. S1 and S2), a distance-weighted autocovariate term was included in all models to take spatial autocorrelation effects into account. This autocovariate was calculated using the autocov_dist function of the spdep R package [37], using the presence/absence of nodules and garden coordinates as input, inverse squared distance weights, a neighbourhood style B and a neighbourhood radius of 0.2. Model accuracy was calculated using nested leave-pairs-out cross-validation (NLPO). To determine the variables that were most strongly related to nodule presence, the importance of the NLPO model predictors was evaluated by calculating the median importance score. Further details are provided in the Supplementary Information.

### Nodule sterilization

Soybean plants were harvested 10–14 weeks after sowing in the gardens. Afterwards, nodules were stored at 4°C on silica. Before sterilization, desiccated nodules were rehydrated individually in sterile tubes with sterile distilled water overnight at 4°C. Sterile glass beads were added and the tubes were vortexed for 1 min. Nodules were transferred to a fresh sterile tube, glass beads and 70% ethanol were added, and tubes were vortexed for 1 to 3 min, depending on the nodule size. Next, the nodules were washed 3 times with sterile distilled water. Subsequently, glass beads were added and nodules were sterilized with 2.5%–3% NaClO for 3 to 5 min by vortexing. Next, the nodules were washed 4 times with sterile distilled water. Finally, nodules were again vortexed in 70% ethanol for 2 min and washed 3 times with sterile distilled water. As a control for sterility, the last wash was plated on tryptic soy agar (TSA) medium [38] and the sterilized nodule was rolled on to a TSA plate that was incubated. Large nodules were cut into equal halves, each placed in a separate 2-ml tube. One tube was immediately snap-frozen with liquid nitrogen and stored at −80°C for use in the nodule microbiome experiment. The other nodule half was used for bacterial isolation. For small nodules that could not be cut into two equal parts, separate nodules from the same plant were used for bacterial isolation and the microbiome experiment.

### Bacterial isolation from nodules

The nodule material was squashed with 50 μl of sterile distilled water using a sterile plastic pestle. Nodule juice was plated on Reasoner's 2A (R2A) [39] and yeast mannitol agar (YMA) [40]. In addition, a 10x, 20x, and 100x dilution was made and then plated on R2A. Plates were incubated at 28°C. The remaining nodule juice was mixed with 50% glycerol and stored at −20°C. Plates were regularly checked for growth and after 14 days, single colonies were selected and replated for purification. For temporary storage, a single colony from the pure isolate was inoculated in 5 ml sterile R2A broth. After incubation at 28°C under constant shaking for 24 h (or until the bacteria had grown well), 0.9 ml of the culture was combined with 0.9 ml of sterile 50% glycerol and stored at −20°C until further use.

### MALDI-TOF MS

Pure isolates were grown on R2A plates at 28°C. Sample extraction, MALDI-TOF mass spectrometry data acquisition, and data analysis were performed as described before [41]. Details are provided in the Supplementary Information.

### Microbiome analysis: 16S rRNA gene and ITS amplicon sequencing

Relative abundances of bacteria and fungi in soil and nodule samples were determined using 16S rRNA gene and ITS amplicon sequencing. DNA was extracted using the DNeasy PowerSoil Pro kit (QIAGEN, Hilden, Germany), according to the manufacturer’s instructions. Sequencing, read processing, and data analysis were performed using established protocols [42–44]. Details are provided in the Supplementary Information.

### Whole-genome sequencing, assembly, and annotation

DNA from 45 selected strains was extracted from colonies grown on R2A plates using the Maxwell RSC Cultured Cells DNA Kit (Promega), and the quantity and quality was checked as described previously [45]**.** The DNA of the strains was then sequenced using the NovaSeq 6000 System (Illumina) at Oxford Genomics Centre, University of Oxford, Oxford, UK, preparing the library with an in-house adapted protocol of the NEB prep kit (New England Biolabs, Ipswich, MA, USA). We also used Oxford Nanopore Technologies (PromethION P24) (Neuromics Support Facility VIB-UAntwerp, Antwerp, Belgium), preparing the library with the native barcoding kit 96 V14. The genomes were then assembled using the Unicycler pipeline for hybrid assembly [46], with the exception of 1200_B8_N1.2, which, due to the large amount of data obtained from long-read sequencing, was assembled with the tool Trycycler [47], using Unicycler to generate the initial assemblies required for the tool. Information on the assembly quality can be found in Table S1. Subsequently, they were annotated using the Bacterial and Viral Bioinformatics Resource Center (BV-BRC) tool [48]. The phylogenetic tree was built using bcgTree with the default parameters, as described previously [49]. In short, bcgTree uses a partitioned maximum-likelihood analysis and Markov models on 107 single-copy core genes found in most bacteria. The number of bootstraps was set at 1,000, and the tree was visualized with the ggtree package in RStudio [50]. Additionally, to assign the species to these strains and assess their similarity, the Average Nucleotide Identity (ANI) values were calculated using the orthoANIu method [51]. For species assignment, the type strains of *Bradyrhizobium* sp., *Rhizobium* sp., and *Tardiphaga* sp. were defined based on the “List of Prokaryotic names with Standing in Nomenclature” (LPSN, https://lpsn.dsmz.de/), and representative genomes were downloaded from the NCBI. The ANI threshold for assignment to a species was set at 95–96% when compared to the respective type strain. Additionally, progressiveMauve [52], with default parameters, was used to align and compare the genomes of interest against the commercial strains. A set of genes was selected for presence/absence screening based on their importance for nodulation and nitrogen fixation. The borders of the symbiotic islands where identified by looking for a region with low GC content, proximity to tRNA-VAL [53], and using Islandviewer 4 [54] to predict the position and length of the symbiotic island. Moreover, to determine if it was a bipartite symbiotic island, the gene *ybgC* obtained from *B. diazoefficiens* USDA 122*,* known to contain a bipartite symbiotic island [53], was located in our genomes using BLAST [55] to identify the starting position of the small B element [56], which was then identified by Islandviewer 4 [54]. Further details are provided in the Supplementary Information.

### Pot trials in sterile vermiculite

Soybean seeds (*G. max* cv. Acardia) were surface sterilized by first washing them in sterile water for 5 min, followed by a 2-min 70% ethanol washing step. Next, the seeds were washed for 10 min in 30% NaClO (12%–13%) stock solution and then washed for 1 min in sterile water. Finally, the seeds were washed 5 additional times for 2 min in sterile water. During each washing step, the seeds were shaken thoroughly. The sterile seeds were pre-germinated on 1% plant agar plates at 22°C in the dark. After 4 days, for each inoculation, 12 seedlings were sown in sterilized vermiculite in small square pots (250-ml size, 2 plants/pot) and grown under a 16-h light/8-h dark photoperiod at 22°C. Using a wick system, plants were watered continuously with nitrogen-poor SOLi solution [57]. After 1 week, plants were inoculated at the shoot/root transition with 1 ml of the indicated bacterial strain diluted to OD 0.01 when in the exponential growth phase. Non-inoculated mock plants and plants inoculated with the *Bradyrhizobium diazoefficiens* strain G49 were used as negative and positive controls, respectively. Four weeks post-inoculation, nodules were counted and nodule colour was noted. A first screening was done to demonstrate the ability of the strains to form red nodules. In a follow-up experiment, the nodule formation of a selection of well-performing strains was also compared quantitatively. Significant differences between the treatments were determined by performing One-way ANOVA (F (7, 88) = 30.7, *P* < .0001) with Tukey multiple comparison correction, using GraphPad Prism version 9.3.1 (Table S2).

### Pot trials in non-sterile substrate

Non-sterilized seeds of five soybean varieties, *G. max* cv. Hermes (Protealis), Acardia (Saaten-Union), Lenka (Prograin), Aurelina (Saatbau Linz), and Gallec (Agroscope/DSP), were sown in pots and grown for 8 weeks in a 16-h light/8-h dark photoperiod at 20°C/10°C. Each six-pot trial consisted of three replicates organized in a randomized block design, using three individual plants for each soy variety-inoculant strain combination per replicate. Pots (4-l size) were filled with 4 l substrate, consisting of 50% (v/v) fresh agricultural soil +50% (v/v) (0/2) sand (particle size 0-2 mm; Leus N.V.), and were placed on trays to avoid cross-contamination. Soil samples were taken to check pH-KCl, %TOC, NO_3_/NH_4_, P, K, Ca, Mg, and Na in ammonium-lactate. On the day of sowing, each pot received 200 ml water, and three sowing holes of ±2.5 cm deep were prepared to sow nine soybean seeds (3x3) of the same variety per pot. Per variety, 35 seeds were soaked in 15 ml of the indicated bacterial strain when in the exponential growth phase (OD 0.01). Non-inoculated mock plants and plants inoculated with the *B. diazoefficiens* strain G49 were used as negative and positive controls, respectively. After sowing the seeds, water was given three times a week, with a starting weight of the whole pot of 3,750 g to avoid overwatering and adding 50 ml on the third and fifth day, which was increased upon plant growth. Two weeks after sowing, seedlings were reduced to three plants per pot by selectively cutting excess plants, ensuring minimal disturbance to the developing root systems. Seedlings were selected based on uniformity in the early growth stage. This resulted in nine plants per variety–strain treatment combination and 45 plants for the null object (non-inoculated mock plants of different varieties) and *B. diazoefficiens* strain G49. Plant growth, yield, and nodulation parameters were determined as described previously [10]. Details are provided in the Supplementary Information.

### Field trials

Two field trials were set up at the beginning of May 2022 at nearby locations (Merelbeke and Bottelare, Belgium), both featuring sandy-loam soils. Two soybean varieties, *G. max* cv. Lenka (Prograin) and RGT Shouna (RAGT), were tested to examine potential *variety × rhizobia* interactions. Seeds were inoculated by coating them in a cement mixer with the indicated bacterial strain when in the exponential growth phase (OD 0.01) and an adhesive (IMPF Signum Soy) with 0.001 ml bacterial culture per seed (estimated concentration of 10^6^ CFU/ml or 1,000 CFU/seed). Non-inoculated mock plants and plants inoculated with the *B. diazoefficiens* strain G49 were used as negative and positive controls, respectively. Seeds were sown within 24 h after inoculation with a sowing machine at a depth of +/− 3.5 cm with a row distance of 25 cm (5 rows per plot). Detailed descriptions of soil characteristics, fertilizer, sowing, and harvesting are provided in Table S3. Field trials were set up in a randomized complete block design (RCBD) with three replicates, at both locations. Each plot comprised a net surface area of 9.375 m^2^. After sowing, pre-emergence herbicides were applied (1 l Arundo (720 g/l dimethenamide-p), 1.5 l Proman (500 g/l metobromuron), and 0.2 l Centium 360 CS (360 g/l clomazon) per hectare). Plant growth, yield, and nodulation parameters were determined as described previously [10]. Details are provided in the Supplementary Information.

### Pot and field trials data analysis

Data analysis was performed using R version 4.4.0 [36]. Pot trials were analysed separately from field trials using generalized linear mixed models (GLMMs) with the *glmmTMB* package [58]. For the field trial data, each location was analysed independently. For field-measured variables, averages over all sampled plants per variety- and treatment-specific plot were used as measurements (see Supplementary Information for details). Data and residual distributions were assessed using QQ-plots to confirm whether model assumptions were met.

For the nodule number in the pot trial data, which follows a count distribution with an excess of zeros, a Zero-Inflated Poisson model was applied. The nodule dry weight, a continuous variable, was modelled using a Zero-Inflated Gaussian model to account for the zero values (when no nodules were formed), while treating non-zero values as continuous data. All other variables in the pot trials were modelled using Gaussian distributions.

The following formulas were used to model the data:

Pot trials (2):


$$ (2)\ Y=V\ast B+\left(1| Trial\right)+\left(1| Trial: Block\right)+\left(1| Pot\right) $$

Field trials (each trial analysed separately) (3):


$$ (3)\ Y=V\ast B+\left(1| Block\right) $$

with “*Y*” being the response variable, “*V*” the fixed effect of the soybean variety, and “*B*” the fixed effect of the *Bradyrhizobium* strain. For pot trials, “Trial” accounts for the random variation between the six trials, because they were analysed together. “Trial: Block” accounts for the random variation among blocks within each trial, with the “Block” nested within the pot trial. The “Pot” effect was included to account for the shared environment of plants within each pot, ensuring that within-pot correlations are considered.

Pairwise comparisons were conducted among the levels of ``*B*'' using the Tukey's Honestly Significant Difference (HSD) test, and means and standard errors were estimated using the emmeans package [59] (*P* ≤ .05).

### Nitrogen fixation capacity measurements

To check the nitrogen fixation capacity of the selected strains isolated from nodules, soybean plants were grown as discussed in the section “Pot trials in non-sterile substrate”. The nitrogenase activity of each root system was determined by measuring the acetylene reduction activity (ARA), as described previously [60]. Details are provided in the Supplementary Information.

## Results

### Gardens with diverse soil types delivered 267 nodules

**Figure 1:**
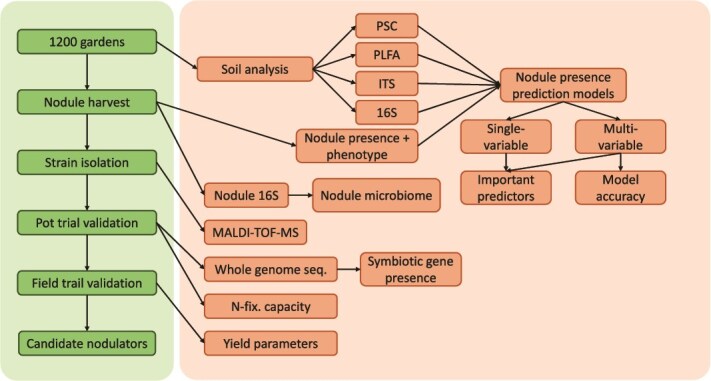
Schematic overview of the sampling and analysis pipeline. In green, the central pipeline to obtain candidate nodulating rhizobial strains is displayed. In orange, the analyses are presented that were performed to investigate nodule presence and the performance of the strains.

The “Soy in 1,000 Gardens” project comprised a pipeline to trap bacteria that nodulate locally grown soybean combined with additional analyses to investigate the biotic and abiotic bulk soil characteristics that influence nodulation success (Fig. 1) [30]. At the start of the citizen science experiment, soil samples were collected to characterize the garden soils. Analysis of physicochemical soil characteristics (PSC), phospholipid fatty acid (PLFA) analysis, bacterial 16S rRNA gene sequencing, and fungal internal transcribed spacer (ITS) sequencing analysis were performed on soil samples from each garden to extract information on the soil texture, nutrient content, microbial biomass, and bacterial and fungal community structure. Soil textures ranged from sandy soil to loam and clay. Nitrate levels were generally low, whereas the phosphorus levels were higher (Figs. S1 and S3). The lower nitrate levels confirmed our assumption that the citizens’ gardens are nitrogen-poor and thus probably conducive to nodulation. The bulk soil 16S rRNA-based microbiome characterization showed that the relative abundance of the known soybean-nodulating *Bradyrhizobium* genus was generally low, with a mean value of 0.018 ± 0.03% (Fig. S1F).

After the initial sampling, citizen scientists reported on soybean growth and yield and, in total, returned 4,436 plants from 1,093 gardens back to the laboratory. Some gardens dropped out because citizens lost interest or because the plots were destroyed (i.e. heavy rainfall, herbivores). Of these plants, 1,004, coming out of 386 gardens, exhibited root nodules or nodule-like structures (Figs. S1A and S4). Nodules with a red interior, indicating active nitrogen fixation, were distinguished from white, non-fixing nodules and brown, necrotic nodules. This can give a first indication of the quality of the symbiosis between the plant and the respective rhizobium inhabiting the nodule. We found 34 nodules with a red interior originating from 27 gardens, 133 white nodules coming from 80 gardens and 100 brown nodules coming from 52 gardens. The 737 remaining nodules were too small to determine the colour and were classified as nodule-like structures or nodules of “unknown” colour. Each nodule was given a unique identifier (e.g. 590_E5_N4), indicating the exact garden (e.g. 590), the plant (e.g. E5), and the nodule (e.g. N4). Plant identifiers (e.g. E5) denote the location of the plant within a 1 m × 1 m grid divided into six columns (A-F) and 10 rows (1–10).

### Can biotic and abiotic bulk soil characteristics predict nodule presence?

**Figure 2:**
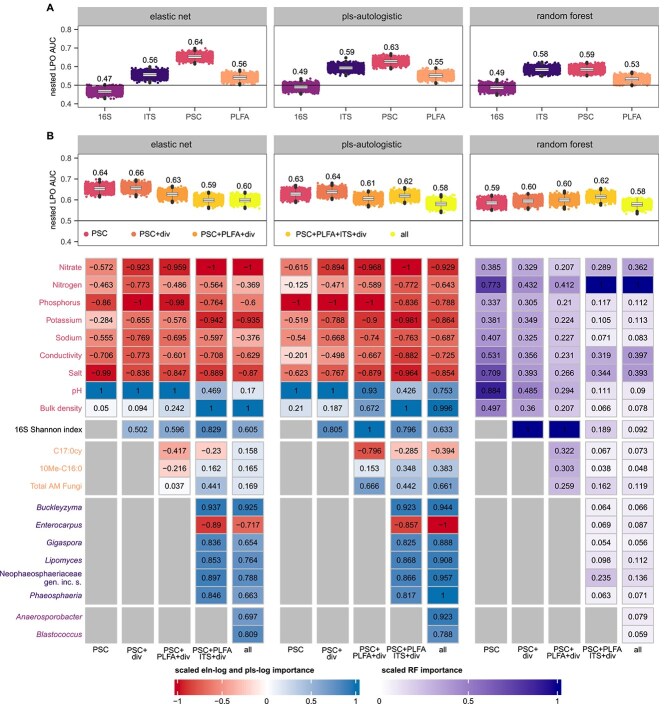
Model accuracy and the importance of soil characteristics to predict nodule presence. (A) Scatter-boxplots of the nested leave-pairs-out AUC of single-data layer models. The dots represent the bootstrapped nested LPO AUC values (*n* = 2,000). The horizontal lines within the boxes indicate the median across plants, while the lower and upper ends of the boxes visualize the first and third quartiles. The lines below and above the boxes extend to the minimum and maximum values, with boxplot outliers marked with black dots. The mean AUC is indicated above each boxplot. (B) Scatter-boxplots of the different data combination models and the PSC model. The importance of the top predictors is visualized in a heatmap below the scatter-boxplots. The importance values correspond with model coefficients for the eln-log models, conditional permutation accuracies for the RF models, and the product between the coefficients of significant PLS components and the variable loadings for the pls-log models. In case a variable is related to multiple significant PLS components, the importance was calculated by taking the sum of these products. The eln-log and pls-log importance values were scaled between −1 and 1, and the RF values between 0 and 1 to improve visualization and model comparisons. The red colour in eln-log and pls-log heatmaps indicates a negative relationship, whereas the blue colour corresponds with a positive one. The white to dark blue gradient indicates the importance of RF predictors. Predictors that were not included in the models are indicated in grey. The row names of the heatmap were coloured to group variables of the same dataset together. Predictors from the PSC data layer are visualized in red, PLFA in orange, ITS in blue, 16S in purple, and the 16S Shannon diversity index in black. div = 16S Shannon diversity data layer.

Because the variability in nodule presence might be related to differences in soil or soil microbiome characteristics, we tried to predict nodule presence from the data gathered in the bulk soil analyses. Prediction models were trained on the bulk soil data using three distinct algorithms: elastic net logistic regression (eln-log), partial least squares auto-logistic regression (pls-log), and random forest (RF). The best-performing single data layer models were the ones based on the PSC data, followed by ITS, PLFA, and 16S rRNA. The AUC values for these models are low, ranging from 0.467 to 0.640, but, except for the 16S rRNA model, they do perform better than randomly assigning nodule presence to gardens (AUC > 0.5) (Fig. 2A, Table S4). This indicates that, apart from the 16S rRNA data, all data layers contain useful information to predict nodule presence, but that additional factors influencing nodule presence are unaccounted for in these data. To investigate whether the datasets contain complementary information, models incorporating different data layer combinations were developed. However, combining different datasets did not improve the prediction accuracy of the nodule presence models (Fig. 2B, Table S4).

### Which factors influence nodule occurrence in Flemish gardens?

Next, we assessed which specific bulk soil variables from the bulk soil datasets were related to nodulation, by training single-variable models and evaluating the best predictors of these and the previously mentioned multi-variable models. The single-variable models give information on significant relationships between each variable and nodule presence, whereas the multi-variable models provide a list of the most important predictors taking into account relationships with other bulk soil variables from the included data layers. Single-variable models showed that nine KEMA variables (total mineral nitrogen, conductivity, nitrate, potassium, salt, phosphorus, sodium, total organic carbon (TOC), and bulk density) were significantly related to nodule presence (Figs. S3 and S5; Table S5). The variables had a negative relationship with nodulation, except for bulk density, suggesting that nodules are more likely to occur in soils with a lower nutrient content. Additionally, models of five fungal (ITS) genera (*Enterocarpus, Nigrograna, Lipomyces, Buckleyzyma*, and *Neophaeosphaeriaceae* genus incertae sedis (gen. Inc. s.)), three bacterial (16S rRNA) genera (*Candidatus Udaeobacter, Blastococcus,* and *Anaerosporobacter*), and the 16S rRNA Shannon Index (measure for bacterial alpha diversity) had a significant relationship with nodule presence (Fig. S5; Table S5).

The PSC, PLFA, and ITS variables with significant links to nodule presence were also important predictors in the multi-variable models, appearing among the top 10 predictors in the majority of the relevant models on different data layer combinations (Figs. 2 and S6). Many of the re-occurring variables, including salt/conductivity, phosphorus, sodium, pH, bulk density, and soil texture, were sometimes missing in the top-10 list of the large models (>1,000 predictors, “Without 16S” and “all” columns in Table S6), but were still present in the top 2–12%. The fungal genera *Enterocarpus, Lipomyces, Buckleyzyma,* and *Neophaeosphaeriaceae* gen. Inc. s. were not present in the top-10 list of the relevant RF models, because these models can capture non-linear relationships and consequently selected different sets of top predictors compared to the other models. However, these genera were still present in the top 5%. Although the bacterial genera data did not contain useful information, the 16S rRNA Shannon Index was found in the top-10 list of multiple multi-variable models. This indicates that to predict nodule occurrence, bacterial alpha diversity is more important than the presence of specific genera. The ITS Shannon Index was a less important predictor, because it was only found in the top 16% of the large eln-log and pls-log models. The total arbuscular mycorrhizal (AM) fungi biomass (or 10Me-C16:0) and C17:0cy phospholipid fatty acid content (Gram-negative bacteria) were mostly found in the top 11% of PLFA-containing models and twice in the top-10 predictor list, respectively. In general, nutrients such as nitrogen/nitrate, potassium, phosphorus, and salt/conductivity were most consistently selected as top predictors in different models, supplemented by fungal and bacterial information, including some fungal genera, AM fungi biomass, and the 16S rRNA Shannon Index (Table S6).

### Characterization of the nodule microbiome

**Figure 3:**
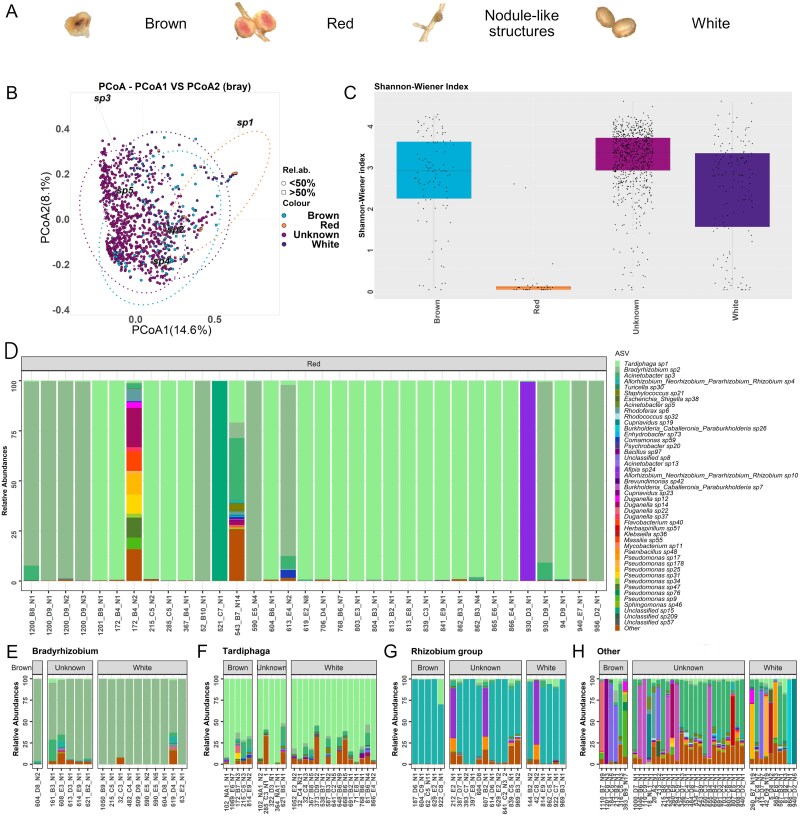
Microbiome of soybean nodules. (A) Pictures of the collected nodules, representing four categories: brown, red, nodule-like structures (indicated as colour “unknown”), and white. (B) Principal coordinate analysis (PCoA) based on Bray–Curtis dissimilarity representing the association between colour phenotype and the nodule bacterial communities. Shape of data points represents whether a nodule contained a dominant ASV (rel. ab. > 50%) or not (rel. ab. < 50%). Orange labels “sp1”, “sp2”, “sp3”, “sp4”, and “sp5” represent *Tardiphaga* ASV1, *Bradyrhizobium* ASV2, *Acinetobacter* ASV3, *Allo-neo-Para-rhizobium* ASV4, and *Acinetobacter* ASV5, respectively. (C) The 16S Shannon index for the different nodule colour groups. Colours of data points (B) and boxplots (C) represent nodule colour groups. The centre lines in the boxplots show the median, with the box limits representing the upper and lower quartiles, and the whiskers representing the maximum and minimum values without outliers, defined as values outside 1.5x the interquartile range above or below the box. Each dot represents a nodule or nodule-like structure. (D) Bar chart representing the microbial community of the red nodules. (E - H) Bar charts representing the microbial community of brown, white, or unknown colour with a dominant ASV (rel. Ab. > 50%) belonging to *Bradyrhizobium* (E), *Tardiphaga* (F), *rhizobium* (G), or another genus (H). The colour legend for panels E-H is the same as for panel D.

The next step was to decipher the bacterial diversity within the nodules and assess how this influences nodule functionality. Hence, 16S rRNA amplicon sequencing was performed, and the bacterial community was compared in nodules with red (indicating active nitrogen fixation), white, brown, or unknown colour (Fig. 3). Permutational analysis of variance (PERMANOVA) revealed that nodule colour weakly, but significantly, correlated with the beta diversity of the bacterial communities (df: 3, *R*^2^: 0.056, *F* value: 19.9, *P* value: 0.001). However, this effect was too small to clearly visualize in a Principal Coordinate Analysis (PCoA) plot (Fig. 3B). Moreover, red nodules presented significantly lower 16S rRNA microbiome Shannon Index values than nodules with a white, unknown, or brown phenotype (Wilcoxon test, *P* < .001) (Fig. 3C, Table S7).

Most of the 34 red nodules were dominated by a single ASV (rel. ab. >50%): 10 of the red nodules were dominated by *Bradyrhizobium* ASV2, and 20 by *Tardiphaga* ASV1 (Fig. 3D). *Afipia* ASV (ASV24) and an unclassified ASV (ASV15, *Xanthobacteraceae*) dominated one nodule each, and the two remaining red nodules contained a highly diverse mix of ASVs (Fig. 3D). The presence of a dominant *Bradyrhizobium* ASV2 was not exclusive to red nodules, and we also observed this in a number of nodules with brown (1), white (10), and unknown colour (5) (Fig. 3E). Every nodule colonized by *Bradyrhizobium* ASV2, regardless of colour, was also colonized by *Tardiphaga* ASV1 and *Acinetobacter* ASV3 in low relative abundances (mean rel. ab. of 0.71 ± 0.96% and 2.53 ± 5.4%, respectively) (Fig. S7A). Similarly, *Tardiphaga* ASV1, which colonized most of the red nodules, was also dominantly present (rel. ab. >50%) in a number of nodules with a brown (5), white (15), or unknown (5) colour (Fig. 3F). In these nodules, except for one white nodule (373_D9_N5), *Bradyrhizobium* ASV2 and *Acinetobacter* ASV3 were consistently present in low relative abundances (average of 1.20 ± 2.14% and 2.70 ± 4.89%, respectively) (Fig. S7B).

Other dominant ASVs include ASV4 and ASV10, belonging to the Allorhizobium-Neorhizobium-Pararhizobium-Rhizobium group and dominantly present in nodules of brown (5), white (6), or unknown (11) colour, but not in red nodules (Fig. 3G). Finally, 39 additional non-red nodules contained a dominant ASV belonging to different genera (Fig. 3H). The rest, and majority, of the non-red nodules and nodule-like structures contained a highly diverse ASV mix with no predominant ASVs (rel. ab. > 50%), hence lacking a main colonizer (Fig. S7C).

### Isolation and identification of indigenous rhizobial bacteria from soybean nodules

In parallel to the nodule microbiome analysis, putative nitrogen-fixing bacteria were isolated from the nodules. Initially, 954 isolates were retrieved, of which 392 isolates were unique after dereplication with MALDI-TOF MS. These originated from 186 nodules with distinct colour phenotypes (32 red, 54 brown, 87 white, and 13 of unknown colour), obtained from 134 plants grown in 93 gardens. Each isolate was given a unique identifier (e.g. 590_E5_N4.2) derived from the nodule it was isolated from (e.g. 590_E5_N4). Sixty genera were represented, with *Bacillus* being the most common (216 isolates, 40% of total isolates), whereas the microbiome analysis showed *Bacillus* sp. in low relative abundance. This discrepancy is probably caused by the relative ease with which *Bacillus* species are isolated with the used media. Conversely, genera that accounted for more than 50% of the relative abundance in the nodule microbiome, such as *Bradyrhizobium, Rhizobium,* or *Tardiphaga*, had less representatives during the isolation (Fig. S8A). Because MALDI-TOF MS only allows for reliable identification up to genus level, isolates belonging to these three genera were also analysed by 16S rRNA gene sequencing, to allow for comparison with the microbiome data. In total, 17 *Bradyrhizobium* strains, belonging to ASV2 and ASV15, seven *Rhizobium* sp. (ASV4 and ASV109), and 26 isolates belonging to the genus *Tardiphaga* (ASV1) were obtained (Figs. S8A and S9A). As expected, most of these isolates originated from red, white, and brown nodules containing either a dominant *Bradyrhizobium, Tardiphaga,* or *Rhizobium* ASV (Fig. S8B and S9A).

### Nodulation capacity of selected strains

To identify the most promising nodulators among the isolated strains, we investigated their ability to induce nodulation on a soybean host in a series of increasingly physiologically relevant environments. First, we used sterile vermiculite and ideal growth conditions (constant light and 22°C). Next, we switched to pots with non-sterile substrate and growth conditions more closely approaching field conditions (20°C/10°C light/dark cycles), and finally switched to field conditions. As positive control, we used *B. diazoefficiens* strain G49 from the commercial inoculant BIODOZ, because it was found to perform well in comparison to the other inoculants tested in Belgian fields [9].

**Figure 4:**
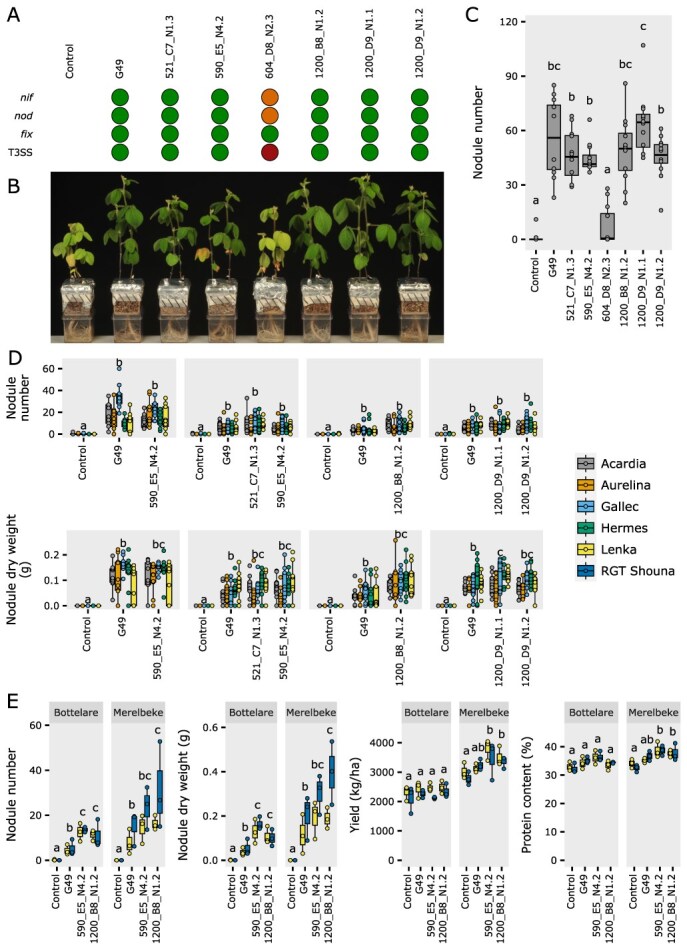
Selection and performance of locally isolated rhizobial strains. (A) Presence of the core set of *nif*, *nod*, *fix* genes, and components of the type 3 secretion system (T3SS) in the commercial strain G49 and six locally isolated *Bradyrhizobium* strains. Green, orange, and red colours indicate the presence of all, some or none of the symbiosis genes that are found in G49, respectively. (B-C) Phenotype (B) and number of red nodules (C) on soybean plants inoculated with these six strains and grown until four weeks past inoculation in sterile vermiculite. For each treatment, 12 plants were analysed, with each plant being represented by a single dot. (D) Nodule number and dry weight for soybean plants inoculated with the indicated strains and grown in a 1:1 mixture of fresh agricultural soil and sand for eight weeks. The four plots for each phenotype represent four of the six different pots per trial (Fig. S11) containing local strains that performed better than the control treatment (no inoculation). For each treatment and variety in each 6-pot trial, nine plants, grouped in three pots, were analysed. (E) Nodule and yield parameters for soybean plants inoculated with the indicated strains and grown in fields at two different locations (Bottelare and Merelbeke). For each treatment, variety, and location, three separate plots were analysed. Nodule and yield parameters were measured across the whole plot, except for nodule number, which was measured for five plants per plot. Seeds inoculated with G49 or non-inoculated (control) seeds were used as a positive or negative control, respectively. Colours of dots and boxplots indicate the soybean variety used. The centre lines in all boxplots (C-E) show the median, with the box limits representing the upper and lower quartiles, and the whiskers representing the maximum and minimum values without outliers, defined as values outside 1.5x the interquartile range above or below the box. Each dot represents a separate plant (C-D) or field plot (E). Significant differences between treatments were determined using one-way ANOVA with Tukey multiple comparison correction (C) or a generalised linear mixed model with the Tukey's honestly significant difference (HSD) test for pairwise differences (D, E). Letters indicate significance groups (*P* value *<*0.05), which in panel D were determined across all pot trials and in panel E for each field trial separately.

First, we selected local strains belonging to the genus *Bradyrhizobium*, *Rhizobium,* or *Tardiphaga*, and assessed their nodulation capacity in pots filled with sterile substrate (Fig. S10). A first screening showed that although the tested strains could induce nodulation, most could only induce small white nodules (Fig. S10). Only eight strains, belonging to the genera *Bradyrhizobium* or *Rhizobium,* consistently produced red nodules (Fig. S10). Then we selected three *Bradyrhizobium* strains (521_C7_N1.3, 590_E5_N4.2, and 1200_B8_N1.2) for a follow-up experiment in sterile vermiculite, as well as *Bradyrhizobium* strains 1200_D9_N1.1 and 1200_D9_N1.2, which were not isolated until after the first screening was performed. These five strains were shown to form red nodules in a quantity at least as high as the commercially used *B*. *diazoefficiens* strain G49 (Fig. 4B,C; Table S8). A sixth *Bradyrhizobium* strain, 604_D8_N2.3, was also tested here as a control for later analyses, because it displayed a significantly lower capacity to induce red nodules (Fig. 4B,C). Correspondingly, plants inoculated with strain 604_D8_N2.3 appeared shorter and less green, when compared to the other five strains (Fig. 4B).

Also in pots filled with non-sterile agricultural soil, all five tested *Bradyrhizobium* strains consistently induced red nodules, in each of five soybean varieties, with nodule numbers and nodule dry weight reaching levels similar to plants inoculated with G49, and in one case marginally but significantly higher, for 1200_D9_N1.1 in terms of nodule dry weight (Figs. 4D and S11; Table S8). Nodule number and strain performance were largely independent of soybean variety (Table S9), and strains that could induce small white nodules in sterile substrate (Fig. S10) were no longer able to do so in agricultural soil (Fig. S11). The five locally isolated strains all displayed a similar nitrogen fixation efficiency compared to G49, as assessed in an acetylene reduction assay (ARA) (Fig. S12 and Table S10).

The nodulation efficacy of two of these local *Bradyrhizobium* strains (590_E5_N4.2 and 1200_B8_N1.2) was subsequently assessed in two field trials at different sites, Merelbeke and Bottelare, using two soybean varieties currently grown in Western Europe (Fig. S13). Based on the availability with breeders at the time, the selected varieties were *G. max* cv. Lenka (Prograin, Canada) and RGT Shouna (RAGT, France). In both field trials, no nodulation was observed on non-inoculated soybean, and strains 590_E5_N4.2 (in Bottelare) and 1200_B8_N1.2 (in Bottelare and Merelbeke) induced significantly more nodules and had significantly higher nodule dry weight than G49 (Figs. 4E and S13A-B; Table S11). Again, the effects on nodulation were independent of the soybean variety.

In Merelbeke, the efficient nodulation appeared to correlate with a darker green and more extensive foliage, as well as an increased grain yield and protein content (Figs. 4E and S13C-E). Indeed, when compared to non-inoculated plants, inoculation with G49 resulted in an increased average grain yield (2.85 ± 0.15 t/ha to 3.25 ± 0.15 t/ha) and protein content (33.2 ± 0.85% to 36.2 ± 0.85%) (Table S11). Inoculation with 590_E5_N4.2 (3.61 ± 0.15 t/ha, 38.5 ± 0.85%) or 1200_B8_N1.2 (3.39 ± 0.13 t/ha, 37.7 ± 0.71%) could increase both yield and protein content even higher, though the difference with G49 was not statistically significant. In Bottelare, a lower yield (2.21 ± 0.14 t/ha) was obtained in non-inoculated plants compared to the yield observed in Merelbeke, and inoculation with G49 or any of the local strains did not result in a significant increase in either yield or protein content compared to the control (Figs. 4E and S13D-E).

We can conclude that despite the variable effects of nodulation on soybean yield in the two field trials, the newly isolated strains 590_E5_N4.2 and 1200_B8_N1.2 performed at least as well as G49 in the field, and might even be able to outperform this commercial inoculant in terms of both yield and protein content. However, further field trials are needed to confirm this.

### Characterization of the phylogeny and presence of the symbiotic genes in the candidate soybean nodulators

Whole-genome sequencing analysis was performed on all the *Bradyrhizobium*, *Rhizobium,* and *Tardiphaga* strains that were tested for their nodulation capacity. The aim was to evaluate the phylogenetic relationships between these isolates and assess the presence of genes involved in the symbiosis, to provide a genetic basis for the performances of these locally isolated strains. The genomes of two commercially used *Bradyrhizobium* strains, G49 and 532C, were included to compare them with our isolates. The assessed genes were subdivided into categories based on their function: nitrogenase synthesis (*nifABDEHKNOQSU* genes), nodulation factor synthesis and transport (*nodABCDIJ* genes), or symbiosis-specific respiration (*fixABCGHIJLNOPQSX* genes) (Table S12). Additionally, genes encoding the structural components of the type III secretion system (T3SS; *rhcVCJLNUQRST, nolU,* and *nopABX* genes) were included, because it was demonstrated that the T3SS, and the effectors it can release, play a positive role in symbiosis and nodulation [61–64].

Most of the strains only contain genes involved in respiration under microaerobic conditions (*fix*), and either belonged to the species *B. baranii*, *T. robiniae*, *R. lusitanum,* or did not belong to any described species (Fig. S9 and Table S13). A few strains, including *B. baranii* 604_D8_N2.3, *R. leguminosarum* 862_C5_N1.2, 814_E9_N1.1 and 62_C5_N11.2, and *R. redzepovicii* 969_B3_N1.2, contain most of the main genes involved in symbiosis but lacked the T3SS (Fig. S9A). For *Bradyrhizobium* strain 604_D8_N2.3, this might explain its inability to efficiently form red nodules (Fig. 4C). Conversely, the *Bradyrhizobium* strains that appeared to perform well in terms of induction of nodulation (521_C7_N1.3, 590_E5_N4.2, 1200_D9_N1.1, 1200_D9_N1.2, 1200_B8_N1.2, Fig. 4C), as well as the commercial strains (G49, 532C), contain all the main genes involved in symbiosis (*nif, nod,* and *fix*) (Figs. 4A and S9). They also contain most of the genes that code for the structural components of the T3SS, including *rhv* (annotated in the genomes as *hrcV, yscCJLNUQRST*) and *nolU* genes (Fig. S9). However, the predicted T3SS component *nopX* could not be found in any of the genomes, *nopB* shared 98% sequence identity with a protein found in the genomes annotated as “nodulation protein” (query cover 56.44%, subject cover 100%), and *nopA* shared a 100% sequence identity with a hypothetical protein in all genomes (Fig. S9B).

**Figure 5:**
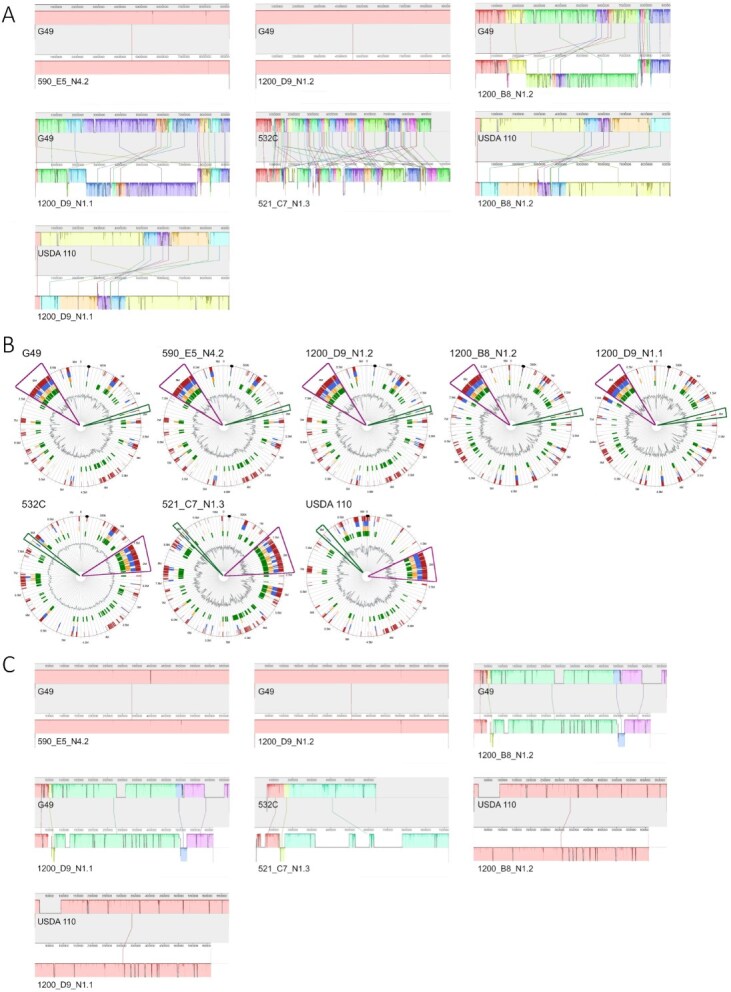
Genomic comparison of the strains of interest and the commercial strains. (A) Full genome alignment, comparing (from left to right, top to bottom) *B. diazoefficiens* G49 with *B. diazoefficiens* 590_E5_N4.2, 1200_D9_N1.2, 1200_B8_N1.2 or 1200_D9_N1.1, *B. japonicum* 532C with *B. japonicum* 521_C7_N1.3, and *B. diazoefficiens USDA 110* with 1200_B8_N1.2 or 1200_D9_N1.1. Each genome is represented in a row, and the blocks with the same colour linked by a line are genetically similar and have no genomic rearrangements. Inversions are represented by blocks found below the horizontal axis of a given track. Empty regions represent parts of the genomes that did not share homology and are unique for the genome concerned. Rearrangements are represented by changes between genomes in the position of coloured blocks relative to other blocks, leading to the crossing of lines linking homologous blocks in the compared genomes. (B) Circular representation of the genomes, presenting the GC content (grey line in the centre) and the predicted genomic islands. Each colour represents a different prediction method; red for integrated, blue for IslandPath-DIMOB, yellow for SIGI-HMM, and green for IslandPick. The purple triangles represent the big element of the bipartite tRNA-Val symbiotic island, whereas the green triangles represent the small element, based on the presence of *ybgC.* The circular genomic representations were generated with IslandViewer 4 [54]. (C) Alignment of the big element of the tRNA-Val symbiotic island comparing (from left to right, top to bottom) *B. diazoefficiens* G49 with *B. diazoefficiens* 590_E5_N4.2, 1200_D9_N1.2, 1200_B8_N1.2 or 1200_D9_N1.1, *B. japonicum* 532C with *B. japonicum* 521_C7_N1.3, and *B. diazoefficiens USDA 110* with 1200_B8_N1.2 or 1200_D9_N1.1. Both alignments (A, C) were preformed using progressiveMauve with the default parameters.

Whole-genome sequencing also confirmed that the isolates of interest belonged either to the species *B. diazoefficiens* (G49, 590_E5_N4.2, 1200_D9_N1.1, 1200_D9_N1.2, and 1200_B8_N1.2) or the species *B. japonicum* (532C, 521_C7_N1.3) (Fig. S13 and Table S13). 590_E5_N4.2 and 1200_D9_N1.2 shared a high genetic similarity (ANI > 99%) with G49 (Table S13). This was confirmed by comparing the genomes using progressiveMauve, where a complete alignment with no reorganizations or inversions in the genomes could be observed between these strains and G49 (Fig. 5A). When 1200_D9_N1.1 and 1200_B8_N1.2 were aligned to G49 (ANI ≈ 98.7%), a large inversion of the genome could be observed, together with some reorganizations (Fig. 5A). These two strains exhibit identical genome rearrangement patterns compared to G49 and are highly similar to each other (ANI = 99.96%; Table S13). Given the high similarity of 1200_D9_N1.1 and 1200_B8_N1.2 with the type strain USDA 110 (ANI ≈ 99.6%; Table S13), an additional comparison between these strains was performed. The results indicated the presence of a complete inversion of the genomes but for a small fragment (Fig. 5A). 521_C7_N1.3 was more similar to 532C (ANI = 96.96%), but contained a larger genome and presented several reorganizations and inversions (Fig. 5A).

The bipartite tRNA-Val symbiotic islands of the five *Bradyrhizobium* strains that contain all main genes involved in symbiosis were also identified (Fig. 5B), and the large element of the island, which contains the *fix, nif,* and *nod* genes, as well as genes encoding components of the T3SS, were extracted and compared to the large element of the commercial strains. Similar to the whole-genome comparisons, the large elements of the symbiotic islands of 590_E5_N4.2 and 1200_D9_N1.2 aligned perfectly to that of G49, whereas those of 1200_D9_N1.1 and 1200_B8_N1.2 presented a couple of inverted regions and were slightly shorter (Fig. 5C). 521_C7_N1.3 presented an inversion when compared to 532C (Fig. 5B,C). Another comparison of the symbiotic islands was conducted using the orthoANIu method. The results showed that the symbiotic islands of strains 1200_D9_N1.1 and 1200_B8_N1.2 were more similar to G49 (99.63%) than to 532C (99.41% and 99.44%, respectively). Nonetheless, the symbiotic islands of these two strains shared more similarity with USDA 110 than to G49 (99.95% and 99.93%, respectively). Conversely, strain 521_C7_N1.3 showed a greater similarity to 532C (99.61%) than to G49 (99.32%). These findings suggest that horizontal gene transfer has likely not occurred between G49 and 521_C7_N1.3, nor between 532C and 1200_D9_N1.1 or 1200_B8_N1.2, because the symbiotic islands appeared to be more conserved within each species than between them (the ANI between G49 and 532C is 99.56%).

## Discussion

There is a growing incentive to promote the cultivation of soybean at northern latitudes, outside the current range of this crop [3, 4, 65, 66]. Several studies have already reported the use of soybean varieties more adapted to a temperate climate [9, 67–71]. To complement this, locally adapted soybean-nodulating rhizobial strains should be identified, and further understanding of how the interaction between soybean variety, the local environment, and rhizobia strain influences soybean yield is needed [21–23, 72–74]. To address both gaps, we designed the “Soy in 1,000 Gardens” project [18, 28, 30]. Using a citizen science approach, we performed a large-scale trapping experiment to identify locally adapted rhizobia that can efficiently nodulate soybean, as well as to generate the data necessary to unravel the subtle and complex interactions between soil parameters, bacterial community, nodulation efficiency, and soybean yield. In addition, the data collected during this project provided novel insights into the factors influencing participation in citizen science, as well as the effectiveness of different strategies for increasing participant retention [31, 32].

The use of soil without a history of soybean growth, and without the use of commercial inocula this entails, should theoretically allow for the identification of native soybean-nodulating rhizobia. However, true indigenous nodulators are expected to be rare in regions where soybean is non-native, because there has been no opportunity for co-adaptation between native rhizobia and the plant [17, 23, 75, 76]. This is reflected in the fact that only 34 red (nitrogen-fixing) nodules were found, in 27 of the 1,200 gardens. Additionally, it may explain why most of the nodules presented a high microbial diversity, suggesting the inability of most of the locally isolated nitrogen-fixing strains to establish a successful symbiosis and/or to prevent other taxonomic groups from occupying the niche. For red nodules, the average 16S rRNA Shannon Index was lower, corresponding with earlier observations stating that effective nodules usually contain a highly dominant nitrogen-fixing strain [77–80]. It should be noted, however, that white and brown nodules sometimes also presented a main colonizer. The bacterial community of these nodules differed marginally from the red nodules, underscoring that the division between red and white or brown nodules is not a perfect proxy for the potential to fix nitrogen. This subset of white and brown nodules could well be nitrogen-fixing nodules that were either immature or in the early stages of senescence, further explaining the lack of nodule colour groupings in the PCoA plots.

The incompatibility between soybean and most local strains might also be reflected by the dominant presence of the non-fixing *Tardiphaga* in many nodules, independent of the colour phenotype. Previously found at low abundances in soybean nodule microbiomes, *Tardiphaga* species do not contain the main nitrogen fixation genes, as revealed by genome sequencing and previous reports [81, 82], and failed to induce nodulation in non-sterile soils. In all nodules colonized by *Tardiphaga*, rhizobial bacteria were present in lower abundances, indicating that this taxon might be able to outcompete local symbionts after the nodule primordia were established by a rhizobial strain. The exact role of *Tardiphaga* as a potential non-rhizobial endophyte in the soybean nodules remains to be determined, whether it acts as a helper, i.e. promoting nodulation and plant growth, or a cheater, just exploiting the nutrient-rich niche found in the nodule.

Despite the rarity of local soybean-nodulating strains, the pipeline led to the identification of five *Bradyrhizobium* strains that consistently performed equally well or better than the commercial strain G49 in terms of nodulation. Moreover, the strains were obtained with a selection pipeline spanning 1.5 years, through a direct assessment of the strain performance in increasingly physiologically relevant conditions. In one of our field trials, two local strains were demonstrated to induce a higher number of nodules compared to G49, correlating with an apparently higher soybean yield and protein content, though the difference with G49 was not significant. For now, it appears that these two strains perform at least as well as G49, though additional trials are necessary before any stronger conclusions can be drawn. Additionally, a dedicated BNF quantification experiment should be conducted to link the performance of the isolated strains (i.e. their ability to fix nitrogen) to any observed increase in soybean yield and protein content. In this study, none of the local strains showed a higher BNF compared to the commercial strains. However, this was determined using ARA measurements, which are known to only provide a rough estimate of the true BNF levels [83–85]. For future studies on this, the more physiologically relevant ^15^N natural abundance method is recommended to measure BNF [86].

Any differences in performance between the locally isolated strains and G49 are likely due to a better adaptation to the local environment and a higher competitiveness in the local soil microbiome. Indicative of this is the observation that the transition from pot to field trials appeared to enhance the performance of the two local strains compared to G49. Interestingly, several of the well-performing strains were genetically highly similar to a commercial strain. Indeed, 590_E5_N4.2 and 1200_D9_N1.2 were highly similar to G49 (ANI > 99.9%), whereas 1200_B8_N1.2 and 1200_D9_N1.1 were highly similar to USDA110 (ANI > 99.6%; Table S13). Moreover, the structure of the genomes of 590_E5_N4.2 and 1200_D9_N1.2 appeared nearly identical to the one of G49, whereas both 1200_B8_N1.2 and 1200_D9_N1.1 presented some rearrangements and inversions with respect to G49 and USDA110, with a complete genomic inversion but for a small fragment in the case of USDA110 (Fig. 5). It could be that these locally isolated strains share a common ancestor with G49 or USDA110, or they could be naturalized and evolved commercial strains. Indeed, it has been observed that locally isolated soybean-nodulating rhizobia are often derived from naturalized commercial inoculants [23, 75]. Inoculants have the ability to survive saprophytically for prolonged periods of time, meanwhile evolving and adapting to the local conditions [75, 76, 87]. How these commercial strains would have arrived in garden soil without history of soybean cultivation remains unknown however. In contrast, 521_C7_N1.3 is rather different from the most closely related commercial strain (532C, Fig. 5), and hence may be an indigenous *Bradyrhizobium* strain that nodulates some legume species native to Flanders [20].

The variation we observed between the two field trials indicates that further field trials in varying environmental conditions are necessary to further illuminate the influence the environment has on soybean nodulation, and to verify the robustness of our isolated strains’ performance. When bacteria are introduced in natural environments, they must compete with the already established microbiome, and survive the changed environmental conditions and different management practices, factors which often hinder the application of an inoculum [88–90].

Also indicative of this environmental complexity is our observation that available soil data, representing both nutrient analysis and microbiome analysis, could only partially predict nodule presence (Fig. 2). This observation implies the importance of unmeasured variables such as the light, water, or temperature conditions the plants grew in. Moreover, the low abundance of nodulating bacteria might overrule and therefore impede the recognition of otherwise favourable environmental conditions. An additional limitation, specific to the soil microbiome analysis, was the necessity to rarefy the samples to relatively conservative read counts. Although the average sequencing depth per sample was high, we preferred to perform alpha diversity analyses on rarefied data, to account for the relatively high inter-sample variability in read depth. Although this approach helps to mitigate bias in diversity estimates, particularly in downstream modelling, it might have limited the detection of rare taxa and lead to a slight underrepresentation of overall diversity. Nonetheless, we were able to identify some factors that likely influence nodulation in Flanders, such as lower nutrient content in the soil, the presence of certain fungal communities such as AM fungi, and a higher 16S rRNA Shannon Index in the soil. Of these factors, lower concentrations of nutrients such as nitrogen and phosphorus, as well as the presence of AM fungi, are known to promote nodule formation in legumes [91–94]. Taken together, future efforts should aim to account for additional environmental variables that may affect nodule presence, using field trials across a range of growing conditions with soybean seeds inoculated with a nodulating strain.

To summarize, by means of a fast and efficient pipeline, this project delivered locally adapted strains, which performed equal to or better than the commercial inoculant strain *B. diazoefficiens* G49 in a complex field environment. The citizen science approach we used effectively enabled us to find these rare, well-performing, local strains by increasing the number of samples that could be taken, and we hence highly recommend the use of citizen science for future trapping experiments in other regions. Our first field trial results suggest that locally adapted rhizobia may boost soybean yield, in particular when combined with locally adapted soybean varieties, but this hypothesis remains to be tested more extensively. If so, identifying locally adapted rhizobia in different regions may help enable profitable and competitive soybean cultivation at more northern latitudes.

## Code availability

The 16S rRNA and ITS processing code, and the single-variable and multi-variable modelling code is available in a zenodo archive (https://doi.org/10.5281/zenodo.12755613).

## Supplementary Material

Supplementary_locally_adapted_soybean_nodulating_rhizobia_wraf152

## Data Availability

Sequencing data have been deposited in the National Center for Biotechnology Information (NCBI) Sequence Read Archive. The accession numbers for the respective BioProjects can be found in Table S14. The environmental and microbiome biomass data, pot and field trial data, and modelling data are made available in a zenodo archive (https://doi.org/10.5281/zenodo.12755613).
