# Supplementary material for: Fast track to environmentally adapted rhizobia for growing soybean at northern latitudes using citizen science
Source: ISME J. 2025 Aug 6;20(1):wraf152. doi: 10.1093/ismejo/wraf152 (PMC13280949; doi:10.1093/ismejo/wraf152)
Supplement: Supplementary_locally_adapted_soybean_nodulating_rhizobia_wraf152 [file supplementary_locally_adapted_soybean_nodulating_rhizobia_wraf152.docx]

Supplementary Information

# Fast track to environmentally adapted rhizobia for growing soybean at northern latitudes using citizen science

Sonia García Méndez^1,2^*, Stien Mertens^1,2^*, Arne Temmerman^1,2^*, Helena Van den Eynde^1,2,3^, Margo Vermeersch^4^, Lena Vlaminck^1,2^, Olivier Berteloot^1,2^, Judith Van Dingenen^1,2^, Alexander Clarysse^1,2^, Annick De Keyser^1,2^, Serge Beullens^5,6^, Ilse de Baenst^3^, Niranjana Roy^3^, Quinten De Paepe^1,2,3^, Jan Michiels^5,6^, Isabel Roldan-Ruiz^4^, Joke Pannecoucque^4,X^, Anne Willems^3,X^, Steven Maere^1,2,X^ & Sofie Goormachtig^1,2,X^

^1^Department of Plant Biotechnology and Bioinformatics, Ghent University, 9052 Ghent, Belgium

^2^VIB-UGent Center for Plant Systems Biology, 9052 Ghent, Belgium

^3^Department of Biochemistry and Microbiology, Faculty of Sciences, Ghent University, 9000 Ghent, Belgium

^4^Flanders Research Institute for Agriculture, Fisheries and Food (ILVO), Plant Sciences Unit, 9820 Merelbeke, Belgium

^5^Center for Microbiology, VIB, 3001 Leuven, Belgium

^6^Center for Microbial and Plant Genetics, KU Leuven, 3001 Leuven, Belgium

*These authors contributed equally to this work

^X^Corresponding author.

# Supplementary Materials and Methods

### PLFA soil analysis

PLFA isolation and analysis were performed on 1,151 samples with a previously described protocol with minor modifications [1]. PLFAs were extracted from 0.75 g freeze-dried material by adding extraction buffer (3 mL ethyl acetate, 1.5 mL 0.8% (v/v) 19:0 PC diluted in ethanol and 0.1 mL ultrapure water) and subsequent sonication for 15 min. After incubation for 15 min at room temperature, 5 mL KCl 0.58% (w/v) was added, samples were vortexed for 10 s, and centrifuged at 2500 rpm and 20°C. Finally, the supernatant was collected and dried under N_2_ at 45°C. Next, PLFAs were purified by solid phase extraction (SPE) using a Discovery DSC-Si (50 mg) SPE 96-well Plate (Merck, Darmstadt, Germany) that was prepared by rinsing with ethanol (3x1 mL), methanol (3x1 mL), and chloroform (3x1 mL). Samples were dissolved in 0.5 mL chloroform, added to the columns, rinsed with 0.5 mL chloroform and 0.5 mL acetone, and finally eluted with methanol (2x1 mL). The samples were dried under N_2_ at 45°C. Next, PLFAs were methylated by adding 0.2 M methanolic KOH to form fatty acid methyl esters (FAME). These were analysed using a capillary gas chromatograph with flame ionisation detector (Thermo Scientific Trace 1300, Thermo Scientific, Waltham, MA, USA) with a Supelco SP-2560 column. PLFAs were identified by retention time using an external FAME (RESTEK Corporation, Bellefonte, PA, USA) and bacterial acid methyl ester BAME mix (Sigma Aldrich, St Louis, MO, USA), and were quantified with a C19:0 internal standard. Total microbial biomass was calculated as the sum of 18 PLFAs (i-C15:0, a-C15:0, i-C16:0, i-C17:0, C16:1c9, C17:0cy, C19:0cy, C14:0, C15:0, C16:0, C17:0, C18:0, 10Me-C16:0, 10Me-C18:0, C18:2c9,12, C16:1c11, and C18:1c9). To cope with the range of starting material contents and bulk density, total microbial biomass was expressed per g of starting material.

### Bulk soil data exploration

All soil data analyses were carried out using R, versions 4.0.3 and 4.1.3 [2]. The datasets analysed included the participant/garden level PSC (n = 1,093), PLFA (n = 1,151), ITS (n = 1,079), and 16S (n = 1,152) data, as well as information on previous fertilisation practices of the citizen scientists, the presence of other legumes in their gardens, and the presence of nodules on the harvested soybean roots. Initial exploration of the 16S and ITS data was performed on ASV level, while for the correlation analyses and models, only genus-level data was used.

Data exploration included a PCA analysis to evaluate batch effects, spatial autocorrelation, and the presence of potential confounding factors and outliers (prcomp function, stats R package [2]). The analysis was performed on z-scored PSC, PLFA, ITS, and 16S data separately (Fig. S2). Spatial patterns were also visualised on a map of Flanders (available at www.geopunt.be) using the tmap and sf packages [3,4] (Fig. S1). Total biomass and batch effects were present in the PLFA data (Fig. S2). These effects were removed by normalising the data points of a given sample for the total biomass of the sample and by performing a batch correction, respectively. For batch correction, linear regression models were used with the total biomass-normalised PLFA variables as independent variables and the batch number as the dependent variable (stats R package). The residuals of these regression models were used as batch-corrected data. Spearman correlations were calculated for the combined soil PSC, corrected PLFA, 16S, and ITS data using the stats R package (Fig. S14).

### Nodule presence prediction modelling

Models were developed to investigate whether bulk soil data can be used to predict the presence of nodules in a given garden and, if so, which soil factors potentially influence nodule presence. Soil PSC data, corrected PLFA data, ITS, and 16S genus-level data, as well as data on the previous fertilisation practices of the citizen scientists, the presence of other legumes in the gardens, and the Shannon index, were used to train single- and multi-variable models. The Shannon index (microbiome *R* package [5]) was calculated using data that was rarefied to an even depth. This standardisation helps to control for sequencing depth as a confounding factor in the diversity estimates. Indeed, while the average sequencing depth was high (149275 for 16S; 25089 for ITS), we observed variability across samples. Therefore, to ensure fair comparisons for diversity indices, used in downstream statistical models, we rarefied all samples to the minimum read count of 786 (16S) and 1000 (ITS) reads (phyloseq *R* package [6]), which represented at least 94% of the maximum observed Shannon diversity (Fig. S15). Only gardens that had a complete dataset were taken into account. As most data layers are only available on the garden level (rather than the individual plant level), the models were trained on the garden level, and a garden was categorised as ‘nodules present’ if at least one plant from the garden exhibited at least one bona-fide root nodule. Gardens with an ambiguous nodule status, i.e., that contained nodule-like structures, but no confirmed nodules, were removed (n_nodule_: 247, n_no nodule_: 401). Different modelling approaches were evaluated, including auto-logistic regression (auto-log), elastic net logistic regression (eln-log), random forest classification (RF), and partial least squares auto-logistic regression (pls-log). As the PCA analyses and variable maps indicated that a spatial structure was present in the data (Figs. S1 and S2), a distance-weighted autocovariate was included in all models to take spatial autocorrelation effects into account. This autocovariate was calculated using the autocov_dist function of the spdep *R* package [7], using the presence/absence of nodules and garden coordinates as input, inverse squared distance weights, a neighbourhood style B and a neighbourhood radius of 0.2. Model accuracy was calculated using nested leave-pairs-out cross-validation (NLPO). To determine the variables that were most strongly related to nodule presence, the importance of the NLPO model predictors was evaluated by calculating the median importance score.

#### Single-variable models

Single-variable auto-logistic regression models predicting nodule presence were developed for each data layer. Individual PSC soil variables were supplemented with information on the citizen scientists’ fertilization practices and the presence of other legumes in the gardens, whereas the ITS and 16S variables included the corresponding Shannon indices. The models for PLFA variables also included TOC and soil type as potential confounding factors. The auto-logistic models were built using the glm function (family:binomial, stats package), with nodule presence as the dependent variable and the factor of interest, potential confounders for this factor, and the distance weighted auto-covariate as independent variables. False Discovery Rate (FDR) correction was performed on the *P* values of the coefficients using the Benjamini-Hochberg procedure [8].

#### Multi-variable models

Three algorithms were evaluated to predict nodule presence as a function of multiple variables and data layers, and the results of the different algorithms were combined to identify a robust set of important predictors. The model algorithms used in this analysis were elastic net logistic regression (eln-log), random forest classification (RF), and partial least squares auto-logistic regression (pls-log). All three algorithms can cope with multicollinearity, which is common in high-dimensional data. The models were trained on different data layer combinations supplemented by the spatial autocovariate. The combinations included each separate data layer, all available data, all data except 16S (PSC+PLFA+ITS+div), PSC+PLFA+div, and PSC+div (with div = 16S Shannon index). The potential confounders TOC and soil type were also included in the multi-variable PLFA models. The training data of the eln-log and pls-log models were z-scored, and for the regression methods, the categorical variables were converted to dummy variables.

The eln-log models were developed using the glmnet function (glmnet R package [9]) with a conditional response variable distribution of the binomial family. The alpha and lambda parameters of the final model were determined through a grid search in a 10-fold cross-validation (CV) setup (caret package [10]; Table S15). The optimal values were selected based on the 10-fold CV area under the receiver operating characteristic curve (AUC).

The pls-log models combined partial least squares (pls) dimensionality reduction with auto-logistic regression models. The pls algorithm removes multicollinearity by calculating latent variables that maximise the covariance between the features and the response variable. By calculating these latent variables, no features are removed. However, the interpretation of the model predictors can become more complicated. Partial least squares dimensionality reduction was performed with the tidymodels and learntidymodels *R* packages. The number of pls components was determined using the 10-fold CV AUC (caret package, Table S15). These components were subsequently used in an auto-logistic model, which had nodule presence as dependent variable, and the pls components and the spatial auto-covariate as independent variables (glm function, stats package).

RF models were created using the randomForest R package. A grid search in a 10-fold CV setup was performed to determine the number of trees (ntree), number of variables randomly sampled at each split (mtry), the number of samples drawn (sampsize), and the minimum size of the terminal nodes (nodesize). The optimal values were selected based on the 10-fold CV AUC (mlr package [11]; Table S15).

#### Model accuracy

Model accuracy was calculated using nested leave-pairs-out cross-validation (NLPO). The idea of NLPO is to hold out pairs of one positive and one negative case as a test set. The remaining data is used to train and fine-tune the model, which is subsequently used to make predictions for the test pair. The hyperparameter finetuning approach of eln-log and pls-log was the same for NLPO as for the final model (grid search using 10-fold CV loop setup in inner NLPO loop), whereas for RF NLPO, a computationally less intensive randomized search in a 10-fold CV setup was implemented, in which 1000 hyperparameter combinations were randomly selected from the hyperparameter space. The NLPO approach applied in this study estimated an average AUC from the predictions of 1,000 random test set pairs containing one garden with and one without nodules. AUC was calculated by averaging over the Heaviside step function with the following formula [12]:

$$\left( 1 \right) \hat{{AUC}_{NPLO}}\left( f_{x} \right)=\frac{1}{\left| I_{+} \right||I_{-}|}\sum_{i\in I_{+}} \sum_{j\in I_{-}} H(f_{\overline{\left\{ i,j \right\}}}\left( x_{i} \right)-f_{\overline{\left\{ i,j \right\}}}\left( x_{j} \right))$$

Where x_i_ and x_j_ are the samples with and without nodules, respectively. $f_{\overline{\left\{ i,j \right\}}}$ denotes a model algorithm trained without the i and j sample test pair. $\left| I_{+} \right|\left| I_{-} \right|$ corresponds to the number of pairs, with I_+_ representing the positive, and I_-_ representing the negative instances. H is the Heaviside step function.

Confidence intervals of the AUC values were determined by bootstrapping the test set prediction pairs 2,000 times and calculating AUCs for each of these bootstrap samples (boot *R* package [13]). The 95% confidence intervals were calculated under the assumption that the sampled AUC values are normally distributed.

#### Predictor importance

To determine the variables that were most strongly related to nodule presence, the importance of the NLPO model predictors was evaluated by calculating their median importance score. The eln-log coefficients of the independent variables were taken as variable importances of the eln-log models. Importance values for the pls-log models were calculated by multiplying the coefficients of the significant PLS components with the loadings of the variables. In case a variable had loadings for multiple significant components, the sum of these products was taken. The conditional permutation accuracy was used as a variable importance metric for the RF models (permimp R package [14]). The importance values were calculated using an RF model without resampling and 1,000 trees. The other fine-tuned hyperparameters of the NLPO models stayed the same. To facilitate model comparison, the importance values of the eln-log and pls-log models were scaled between -1 and 1 before the median value was calculated, while the RF importance values were scaled between 0 and 1.

### MALDI-TOF MS

Pure isolates were grown on R2A plates at 28°C. Protein extracts for MALDI-TOF mass spectrometry analysis were prepared as described before [15]. All extracts were spotted in duplicate as technical replicates. Each sample spot was overlaid with 1 µL of matrix solution (10 mg mL−1 α-cyano-4-hydroxycinnamic acid in acetonitrile:water:trifluoroacetic acid 50:47:5:2:5) and MALDI-TOF MS profiles were acquired on a Bruker MicroflexTM LT/SH (Bruker Daltonik, Bremen, Germany) as previously described [15]. For identification, spectra from a specific mass range (2-20 kDa; mainly corresponding to ribosomal proteins) were selected and compared to the Bruker BDAL (9607 MSP) and the LM-UGent *in house* databases using MBT Compass Explorer according to the manufacturer’s guidelines (Bruker Daltonik, Bremen, Germany). Identification scores were interpreted following Bruker’s instructions as ‘highly probable species identification’ (2.300-3.000), ‘secure genus identification’ (2.000-2.299), ‘low-confidence identification’ (1.700-2.000), or ‘no identification possible’ (<1.700). For dereplication (i.e. grouping of strains according to similarity of MALDI-TOF MS profiles and selection of representative isolates) the SPeDE software tool was used as described previously [15]. For cluster analysis, profile similarities were calculated using the curve-based Pearson product-moment correlation coefficient and the unweighted pair group method using arithmetic average (UPGMA) clustering algorithm was used in BioNumerics 7.6.3 (Applied Maths). It should be noted that, because the available databases are primarily designed for clinical samples and lack many protein profiles of environmental bacteria, the method is considered fully reliable only at the genus level. Consequently, in this study, MALDI-TOF MS was used solely for identification at the genus level.

### Soil microbiome analysis: 16S rRNA gene sequencing

The 1,153 soil samples were stored in -70°C in 2mL Eppendorf tubes. DNA was extracted with the DNeasy PowerSoil Pro kit (QIAGEN, Hilden, Germany). The V4 region of the 16S rRNA gene was PCR-amplified as described previously [16]. The amplicons of all soils were pooled and sequenced on the Illumina NovaSeq 6000 instrument with the following parameters: NovaSeq6000 flowcell SP 500 kit v1.5., paired-end reads (251-12-12-251), 130pM/120pM + 1% PhiX (VIB, Nucleomics Core, Leuven, Belgium). After sequencing, the samples were demultiplexed and the primers were removed. The reads were trimmed by means of the DADA2 package in RStudio, by truncation at 200 and 180 bp for the forward and reverse reads, respectively, and with the default quality parameters [trunQ = 2; maxEE = c(3,3)]. After filtering, the amplicon sequence variants (ASVs) were generated as described previously [17]. Subsequently, the taxonomy was assigned with the Silva vs138 database using the function IdTaxa (DECIPHER) and the default minimum bootstrap confidence (100%), . The database used in this study was the Silva vs138 database [18,19]. The reads belonging to chloroplast (Class Chloroplast) and mitochondrial (Order Rickettsiales) DNA were removed, reducing the read count by 0.109% on average. Also, ASVs that did not represent four reads in at least one sample were discarded. The final mean read count per sample was 149,275.4, and the library yielded 172,114,563 reads corresponding to a total of 19,235 ASVs across the full dataset. All the ASVs that were not classified at phylum level were subsequently removed from the analysis. Moreover, the 175 ASVs belonging to the Archaea (phylum Crenarchaeota, Euryarchaeota, Halobacterota, and Thermoplasmatota) were kept during the analysis.

### Soil microbiome analysis: ITS amplicon sequencing

The 1,145 soil samples were stored in -70°C in 2mL Eppendorf tubes. DNA was extracted with the DNeasy PowerSoil Pro kit (QIAGEN, Hilden, Germany). The fungal rDNA-ITS2 region was PCR-amplified as described previously [20], with some adjustments of the protocol. Mastermixes for all PCRs were prepared using the iProof High-Fidelity PCR Kit (Bio-rad, Hercules, USA). The total reaction volume of the first PCR was 20 μL (1 μL template, 4 μL buffer, 1 μL dNTP, 1 μL FW primer, 1 μL REV primer, 0.5 μL polymerase, 11.5 μL distilled water) and for the second PCR 40 μL (2 μL template, 8 μL buffer, 2 μL dNTP, 10 μL FW primer, 10 μL REV primer, 1 μL polymerase, 7 μL distilled water). Each PCR was followed by a PCR product clean-up using magnetic bead purification with HighPrep PCR beads (CleanNGS) (Cleanna, Waddinxveen, The Netherlands). The amplicons of the soils were pooled and sequenced in 4 runs on an Illumina MiSeq instrument, using the Reagent Kit v2 for 500 cycles (4.5 pM + 24.10% PhiX v3), and generating paired-end reads (251-8-8-251) (VIB, Nucleomics Core, Leuven, Belgium). After sequencing, the samples were demultiplexed. Primers and adapters were trimmed using the cutadapt tool [21], DADA2 [22], ShortReads [23], and Biostrings [24] *R* packages (*R* version 3.6.0 [25] *R* core team, 2019). Sequences with ambiguous bases and low-quality tails were removed using the DADA2 filterAndTrim function [22]. The minimum length of this function was set to 50 bases and the truncation length (204 to 150) was determined with the Figaro tool [26], which analyses the error rates in the FastQ files to determine the optimal trimming parameters per run. The quality parameters were set at trunQ = 2 and maxEE = 5. The generation of the ASVs was performed as described previously [17]. The taxonomy of the ITS data was assigned with the naive Bayesian classifier method (assignTaxonomy function in the DADA2 package [22]) using the UNITE database (V9 [27]). The confidence intervals of the assignments were determined using a default minimum bootstrap sample of 50. Samples that had less than 5,000 reads were not included in the analysis. Chimeras, sequences of which the phylum was not identified, and ASVs that did not represent four reads in at least one sample were discarded from the dataset. The final average read count was 25,089, the total library had 27,071,472 reads corresponding to a total of 40,619 ASVs across 1,079 samples. Some samples were resequenced to improve read count, in these cases the samples with the highest read numbers were used for modelling to allow the evaluation of batch effects.

### Nodule microbiome analysis: 16S rRNA gene sequencing

The 1,094 nodules that were stored in -70°C in 2-mL Eppendorf tubes after sterilisation were kept on dry ice. Two 5-mm stainless-steel balls were added and the nodules were flash-frozen in liquid nitrogen before homogenisation using a bead beater for 2 times 1 min at 25 Hz. Subsequently, DNA was extracted with the DNeasy PowerSoil Pro kit (QIAGEN, Hilden, Germany). First, the Powerbeads from the kit were added to the Eppendorf tubes before following the provided protocol starting from the vortexing step. The V4 region of the 16S rRNA gene was PCR-amplified as described previously [16]. The amplicons of all nodules were pooled and sequenced on an Illumina NovaSeq 6000 instrument following these parameters: NovaSeq6000 flowcell SP 500 kit v1.5., paired-end reads (251-12-12-251), 130pM/120pM + 1% PhiX (VIB, Nucleomics Core, Leuven, Belgium). After sequencing, the samples were demultiplexed and the primers were removed. The reads were trimmed by means of the DADA2 package in RStudio, by truncation at 230 and 180 bp for the forward and reverse reads, respectively, and with the default quality parameters [trunQ = 2; maxEE = c(3,3)]. After filtering, the amplicon sequence variants (ASVs) were generated as described previously [17]. Subsequently, the taxonomy was assigned using the function IdTaxa (DECIPHER), and the default minimum bootstrap confidence (100%). The database used in this study was the Silva vs138 database [18,19]. The reads belonging to chloroplast (Class Chloroplast) and mitochondrial (Order Rickettsiales) DNA were removed, reducing the read count by 83.86% on average. Samples with a read count below 1,000 reads were eliminated, and those ASVs that did not represent four reads in at least one sample were discarded from the dataset. The final mean read count was 67,288.63, and the library yielded 73,546,471 reads corresponding to a total of 23,766 ASVs across the full dataset. All the ASVs that were not classified at phylum level were subsequently removed from the analysis. Moreover, the 39 ASVs belonging to the Archaea (phylum Crenarchaeota and Thermoplasmatota) were kept during the analysis.

### Statistical analysis of the nodule microbiome

*R* version 4.2.1 was used for the statistical analysis of the nodule microbiome. For the samples’ beta diversity (n = 1,003), a dissimilarity matrix based on the Bray-Curtis dissimilarity index was generated with the function vegdist in the vegan package [28] using the ASV count tables as input. The differences between the samples due to colour phenotype were checked using the Permutational Multivariate Analysis of Variance (PERMANOVA), using the function adonis2 in the vegan package (n_red_: 34 , n_brown_: 100 , n_white_: 133 , n_unkown_: 736). Variance homogeneity between groups was verified by calculating the Multivariate Homogeneity of Groups Dispersions with the betadisper function within the vegan package (df: 3, F: 27.932, nr permutations: 999, p: 0.001). Principal Coordinate Analysis (PCoA) plots were generated with the package MicrobiotaProcess [29]. The analyses were done at ASV level.

For analysis of the samples’ alpha diversity, the ASV count tables were rarefied to the sample with the smallest read count of 1,008, with the function rarefy_even_depth in the phyloseq package. The Shannon-Wiener index was calculated with the function alpha in the package microbiome. We performed a Kruskal-Wallis test by using the function kruskal.test in *R* (X^2^: 145.81, df: 3, p:2.12e^-31^), followed by a two-sided Wilcoxon signed-rank test with the function pairwise.wilcox.test to explore whether the nodule colour phenotype was associated with differences in the alpha diversity of the samples. The *P* values were adjusted with the Holm–Bonferroni method. To confirm these results, we also created mixed models with the lme4 R package [30], integrating the soil and the plants the nodules came from as random effects and nodule colour as fixed effect. Subsequently, we performed a pairwise analysis with the emmeans R package (Table S16) [31].

### Whole-genome sequencing gene selection

We screened for the presence of genes of importance for nodulation and nitrogen fixation (Fig. S9, Table S12). For the nitrogenase synthesis (*nif* genes), we included structural genes (*nifHDK*), regulators of nitrogen fixation (*nifA*), genes involved in the formation of the iron–sulphur cluster (*nifUS*), and the synthesis of the iron–molybdenum cofactor (*nifB*, *nifEN*, *nifOQ*). For nodulation factors (*nod* genes), we determined the presence of genes involved in their synthesis (*nodABC*), transport (*nodIJ*), and the regulation of *nod* gene expression (*nodD*). For symbiosis-specific respiration (*fix* genes), we also included genes involved in electron transfer to the nitrogenase (*fixABCX*), in respiration under the microaerobic conditions (*fixGHIS* and *fixNOQP*), and in low oxygen-dependent induction of nitrogen fixation (*fixLJ*). Finally, we also assessed the presence of genes encoding the conserved structural components of the type III secretion systems (T3SS) [32], including *rhcV* (*LcrD*, *HrcV*, *EscV*, *SsaV*), *rhcC1* (*YscC*, *MxiD*, *HrcC*, *InvG*), *rhcJ* (*YscJ*, *HrcJ*, *EscJ*, *PscJ*), *nolU*, *nolV* (*YscL*), *rhcN* (*YscN*, *SpaL*, *MxiB*, *HrcN*, *EscN*), *rhcU* (*YscU*, *SpaS*, *EscU*, *HrcU*, *SsaU*), *rhcQ* (*YscQ*), *rhcR* (YscR, SpaR, HrcR, EscR), rhcS (YscS), rhcT (YscT, HrcT, SpaR, EscT, EpaR1), as well as *nopA*, *nopX*, *nopB*, which are predicted to encode components of the secretion system. To corroborate the annotation of these genes, the proteins were blasted against the UniProt database [33]. The position of *nif*, *fix*, and *nod* genes, and of the genes encoding the T3SS within the symbiotic island was represented with an arrow chart generated with the *R* package geneviewer [34].

### Non-sterile substrate pot trial measurements

At 8 weeks, plant height was measured and plants were harvested. Per plant, shoot dry weight, the number of nodules, and nodule dry weight were determined. Chlorophyll content was measured using a CCM-200 m (Opti-Sciences Inc., Hudson, USA), as described previously [35]. The output was expressed as chlorophyll content index (CCI), defined as the ratio of transmission at 931 to 653 nm through a leaf. Measurements were conducted on the youngest fully expanded leaf of each of the plants.

### Field trial measurements

Chlorophyll content was measured at the reproductive growth stage R5, using a CCM-200 m (Opti-Sciences Inc., Hudson, USA). The output was expressed as CCI, and measurements were conducted on the youngest fully expanded leaf of 10 plants of each plot and the average was calculated. Five randomly chosen plants per plot were harvested in the front and the back of the plot (ten plants per treatment). When nodules were present, they were counted and weighed. Nodule dry weight was determined after drying the nodules for 72 h at 70°C. The remaining plants were harvested at full plant maturity (growth stage R8) with a trial field thresher. After harvest, soybean yield per plot was measured in kg ha^−1^ and adjusted to 0.150 kg H_2_O kg^−1^ seed. Thousand kernel weight was determined for dry soybeans after drying for 72 h at 70°C. Finally, the protein content of the soybeans was determined on dry material milled over a 1-mm screen using a cutting mill (Peppink Type 200AN). Protein content was estimated using near-infrared reflectance spectroscopy (NIRS). Calibration was based on soybean samples from 2014 to 2017 from different soybean trials in Belgium.

### Nitrogen fixation capacity measurements

To check the nitrogen fixation capacity of the selected strains isolated from nodules, soybean plants were grown as discussed in the section ‘Pot trials in non-sterile substrate’. For each treatment, at least 9 plants were harvested 4 weeks post-inoculation. For each plant, nodules were counted, and nodule fresh weight and dry weight were measured. Additionally, the nitrogenase activity of each root system was determined by measuring the acetylene reduction activity (ARA), as described previously [36]. Briefly, ethylene production was quantified using a Hewlett-Packard 5890A gas chromatograph (Agilent Technologies, Santa Clara, CA, USA) equipped with a PLOT fused silica column, with propane as an internal standard. Finally, ARA is reported as μmol of ethylene produced per h. Significant differences between the treatments were determined by performing One-way ANOVA (F (6, 71) = 3.964; p = 0.0018) with Tukey multiple comparison correction, using GraphPad Prism version 9.3.1 (Table S10).

# Supplementary Figures


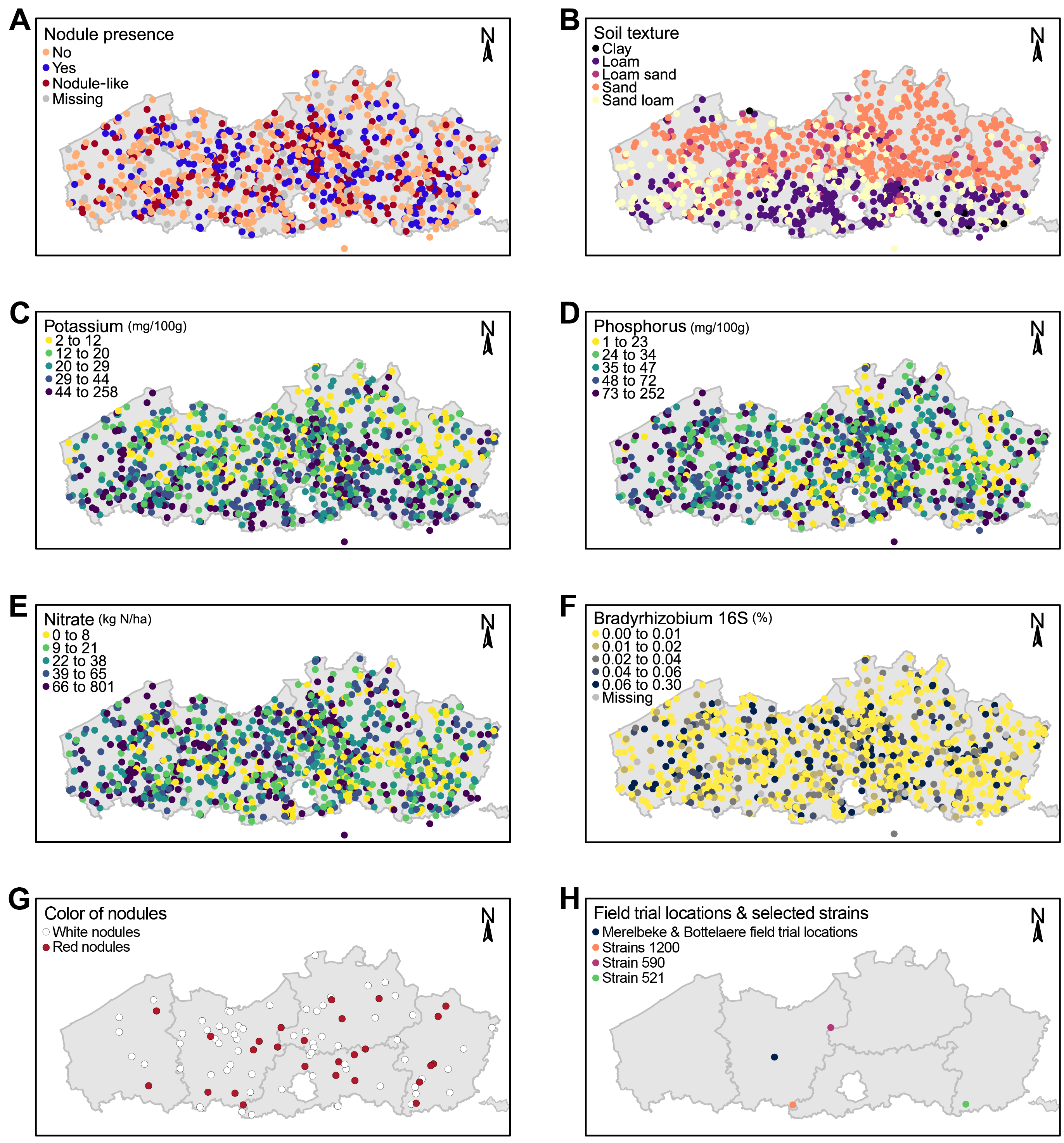


###### **Figure S1:** Maps visualising the spatial distribution of gardens and selected soil, nodule, and microbiome characteristics in Flanders, Belgium. (A) Presence of nodules, (B) soil texture, (C) potassium (mg/100g), (D) phosphorus (mg/100g), (E) nitrate (kg N/ha), and (F) *Bradyrhizobium* relative abundance. G) Gardens where white and/or red nodules were found. Gardens where both red and white nodules were found are also marked as red. H) Location of the field trials and the gardens where the strains selected for validation were isolated. ‘Strains 1200’: 1200_B8_N1.2, 1200_D9_N1.1, and 1200_D9_N1.2. ‘Strain 590’: 590_E5_N4.2. ‘Strain 521’: 521_C7_N1.3.


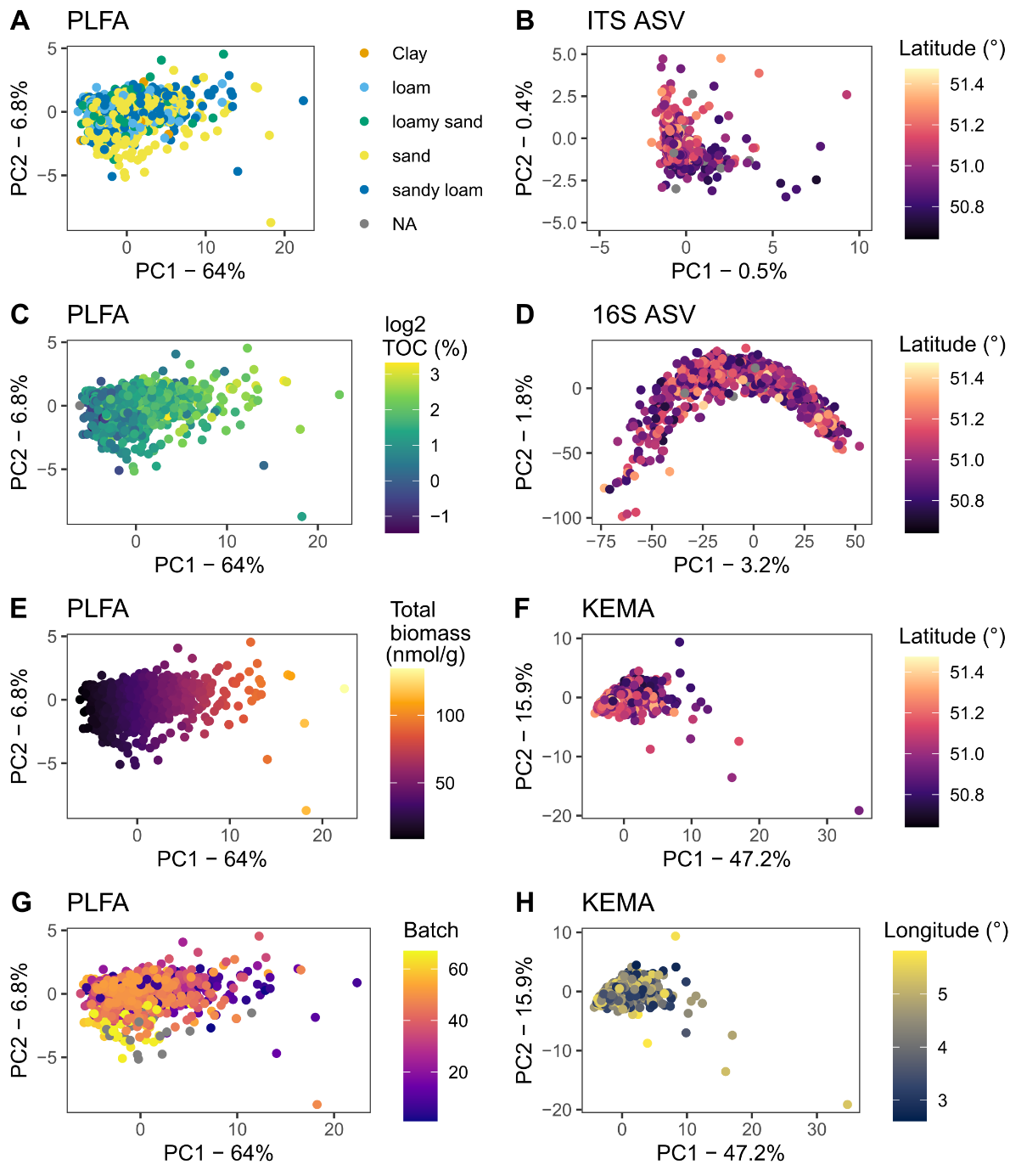


###### **Figure S2:** PCA plots for PLFA, 16S, ITS, and PSC data. On the left, PLFA PCAs visualise the effects of soil texture (A), TOC (%) (C), total biomass (nmol/g dry soil) (E), and batch effects (G) on the data. The effects of latitude (°) or longitude (°) on ASV-level ITS (B), ASV-level 16S (C), and PSC data (F and H) are visualised on the PCAs on the right-hand side of the figure.


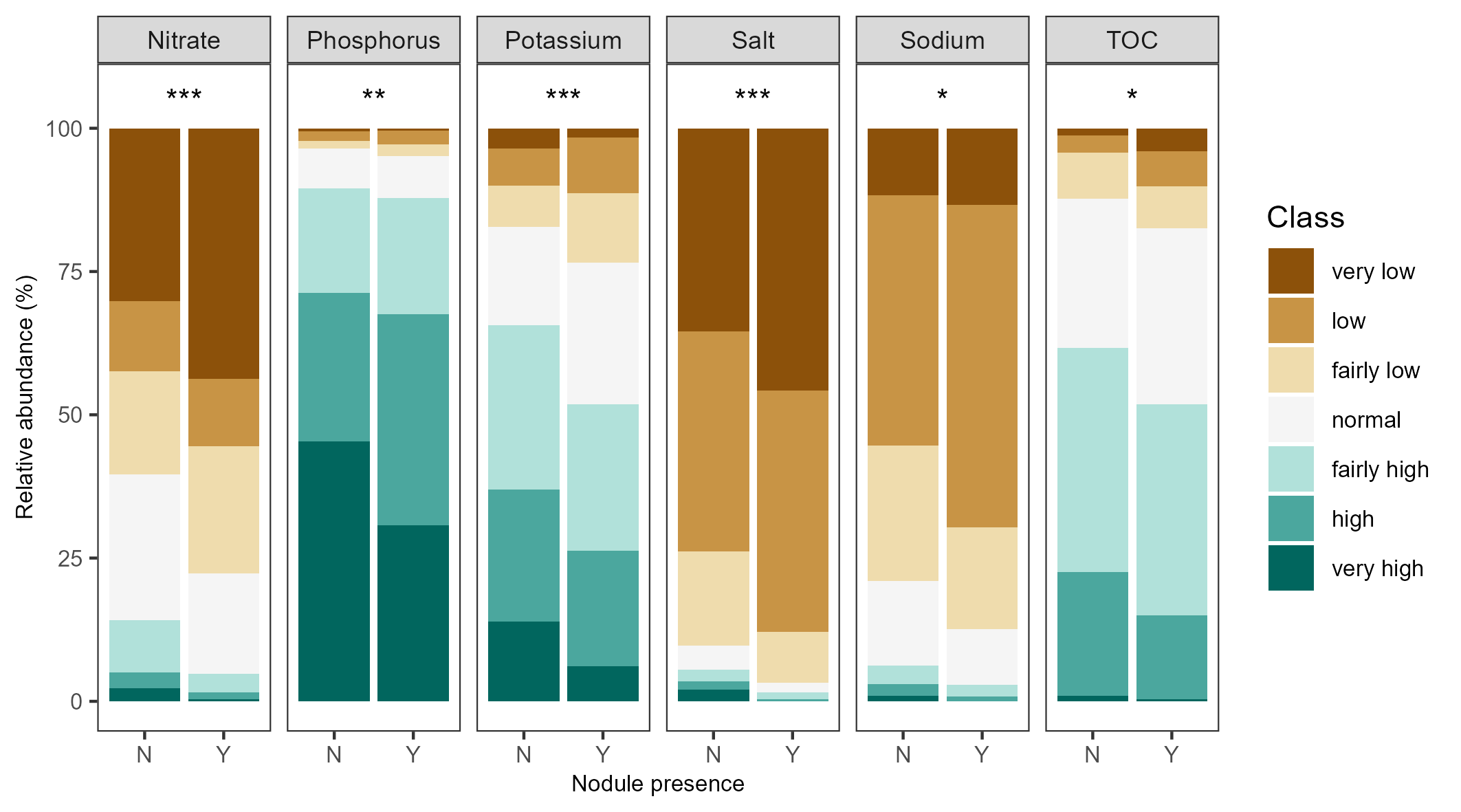


###### Figure S3: Relative abundance of physicochemical soil classes in gardens with and without nodules. Nitrate, phosphorus, potassium, salt, sodium, and TOC levels in the soil are subdivided into classes defined by the Soil Service of Belgium, taking the soil texture into account [37]. *: *P*< 0.05, **: *P* < 0.01, ***: *P* < 0.001. *P* values are calculated from univariate auto-logistic models trained on the raw data with Benjamini-Hochberg FDR correction.


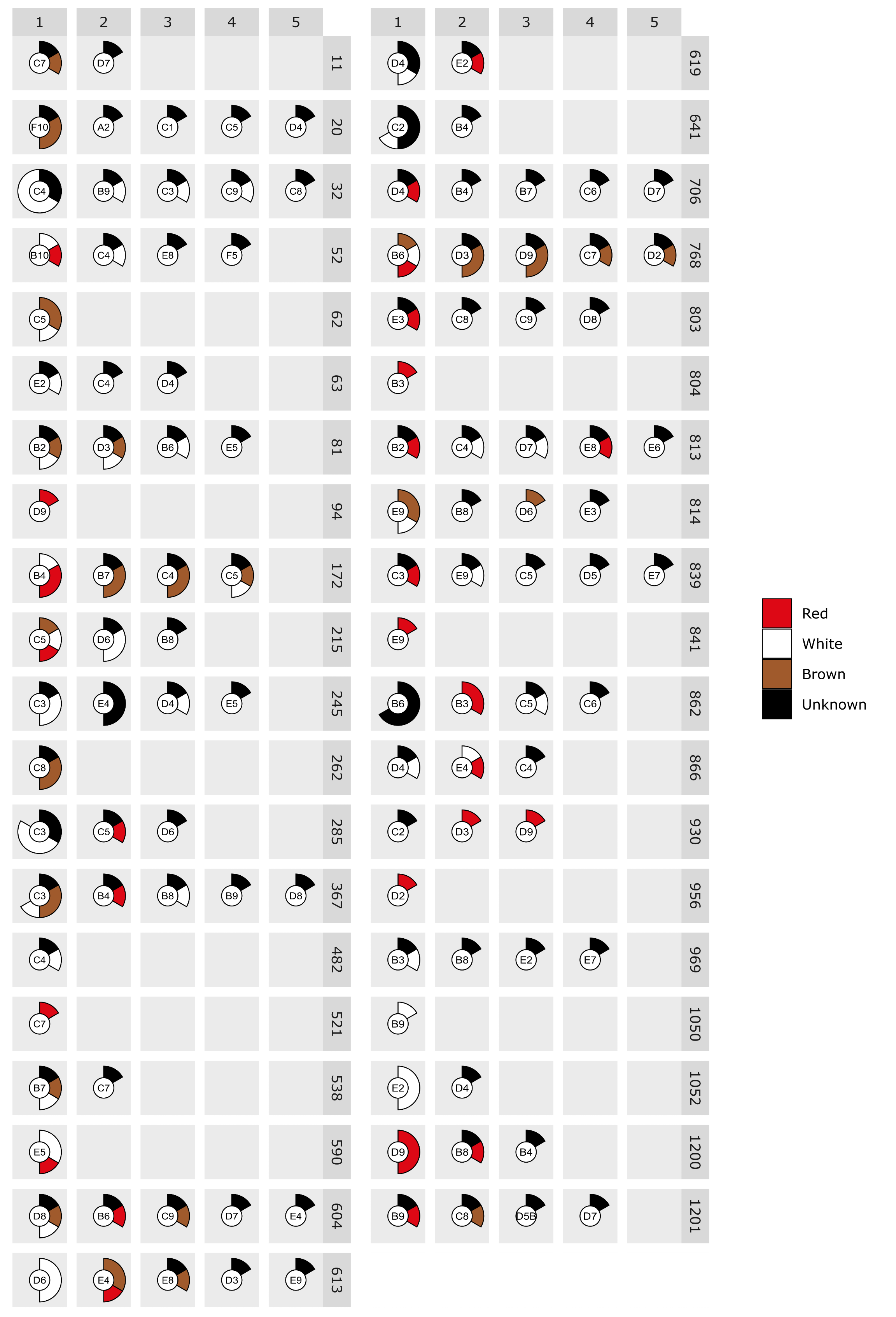


###### **Figure S4:** Nodule colours from the nodules harvested from gardens from which one or more strains used in any follow-up experiments in this study were isolated. Row numbers indicate garden numbers. Column numbers indicate up to five different plants from the same garden. Each graph indicates, for each plant, the plant label in the centre, as well as which nodule colours were found, with a full circle indicating a maximum of 6 nodules.


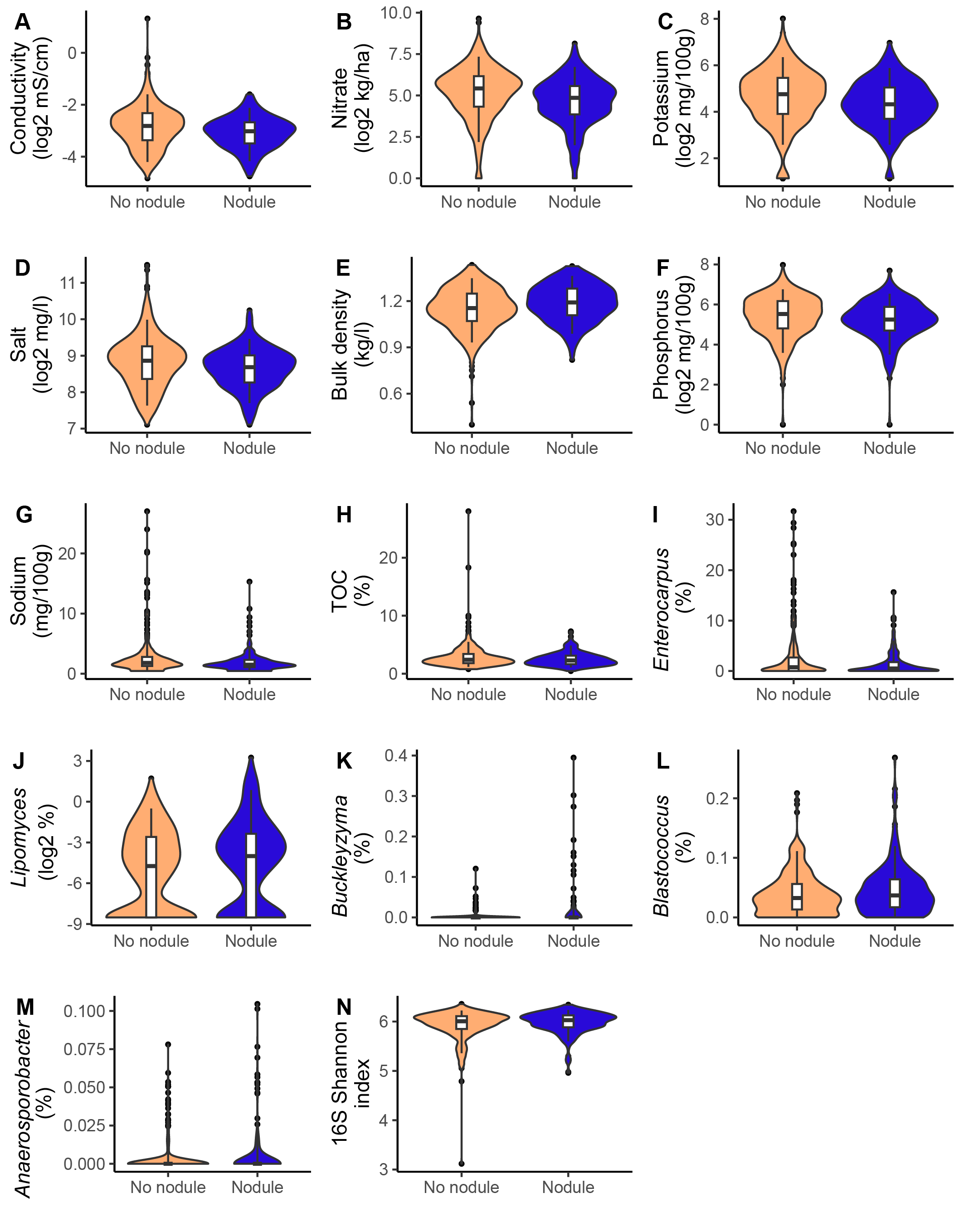


###### **Figure S5: The difference in significant PSC, ITS, and 16S rRNA variables between gardens with and without nodules.** The violin and boxplots show the difference in log-transformed (A) conductivity (EC, mS/cm), (B) nitrate (kg N/ha), (C) potassium (K-AL, mg/100 g soil), and (D) salt (mg/L soil) levels, (E) bulk density (kg/L soil), (F) log-transformed phosphorus (P-AL, mg/100 g soil) and (G) sodium (Na-AL, mg/100 g soil) levels, (H) TOC (%), and the relative abundances (%) of (I) *Enterocarpus*, (J) log-transformed *Lipomyces*, (K) *Buckleyzyma*, (L) *Blastococcus*, and (M) *Anaerosporobacter*, and (N) the 16S Shannon index. The centre lines in the boxplots show the median, with the box limits representing the upper and lower quartiles, and the whiskers representing the maximum and minimum values without considering outliers, defined as values outside 1.5x the interquartile range above or below the box. Outliers are marked with black dots (note that the lines passing through the outliers are violin plot contours, not whiskers).


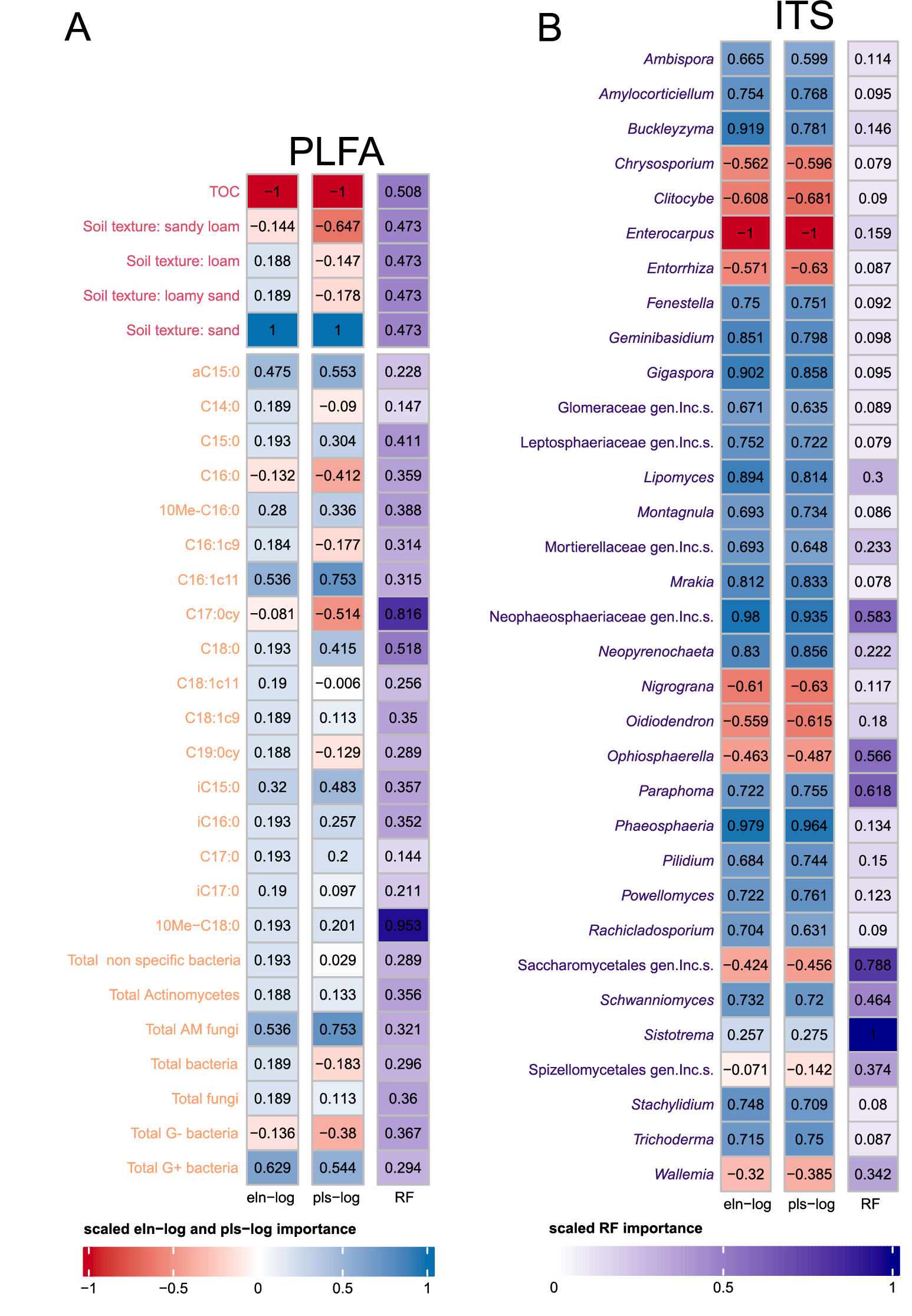


###### **Figure S6: Predictor importance for the multi-variable PLFA and ITS models.** (A) Importance of PLFA predictors and (B) importance of ITS predictors. The importance values for eln-log models are the variable coefficients, for the RF models the conditional permutation accuracy, and for the pls-log models the product between the coefficients of significant PLS components and the variable loadings of the pls-log models. In case a variable is related to multiple significant PLS components, the importance is calculated by taking the sum of these products across the components concerned. Eln-log and pls-log importance values were scaled to values between -1 and 1, and the RF values between 0 and 1 to facilitate visualisation and model comparison. The red colours in the eln-log and pls-log heatmaps indicate negative relationships, whereas blue colours correspond with positive relationships. For RF model importance values, a white to dark blue gradient is used. PLFA, ITS, and PSC predictor variables are indicated in orange, blue and red font, respectively.


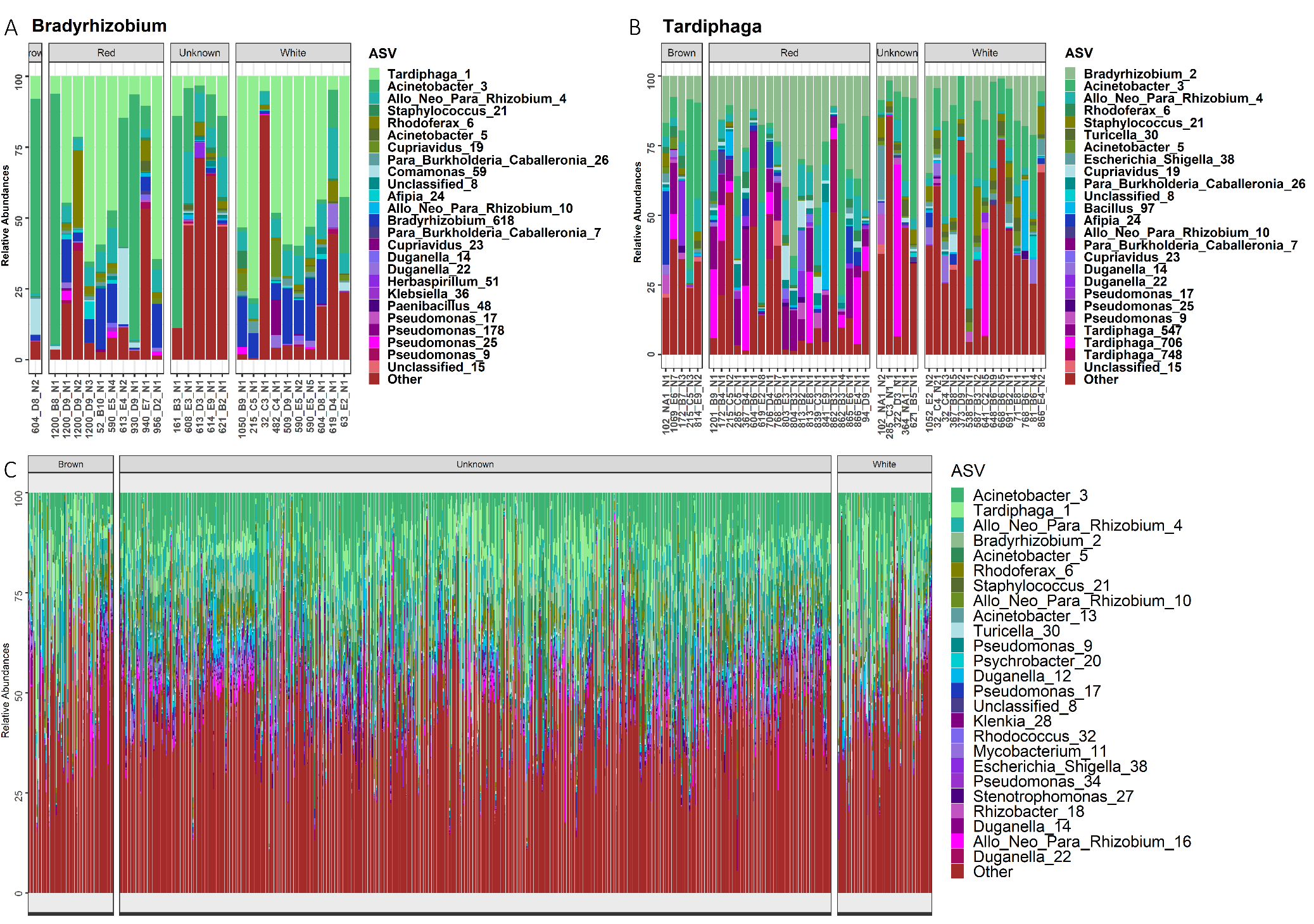


###### **Figure S7:** (A) Bar chart representing the bacterial community on the nodules that are mainly colonized by *Bradyrhizobium* ASV2, when ignoring this ASV. (B) Bacterial community of the nodules that are mainly colonized by *Tardiphaga* ASV1 when ignoring this ASV. (C) Bacterial community of the nodules and nodule-like structures that did not present a predominant ASV.


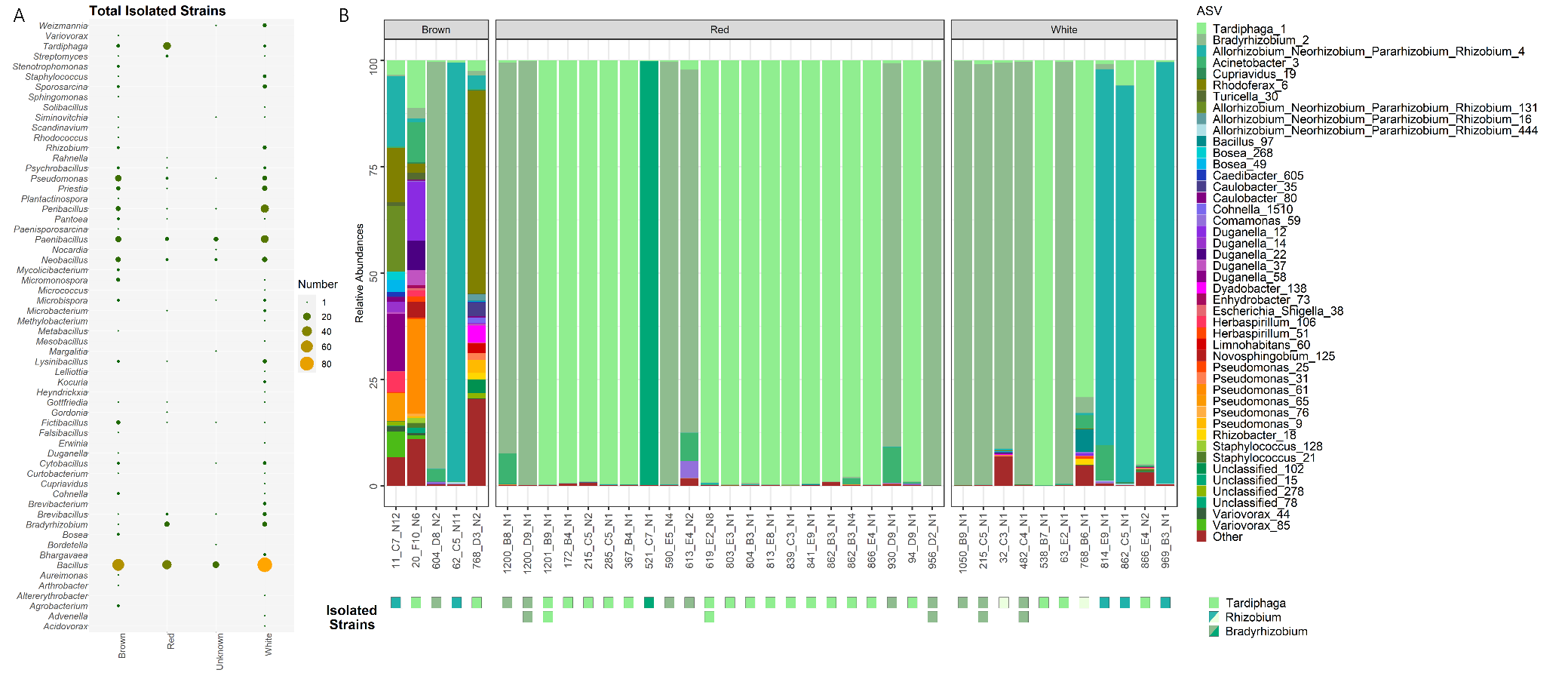


###### **Figure S8:** (A) Bubble plot representing the isolated diversity from the nodules grouped by the nodule colour phenotype. Isolates were identified down to genus level using MALDI-TOF MS. Colours indicate the number of strains isolated from the indicated type of nodule. (B) 16S microbiome of the nodules where the *Tardiphaga*, *Bradyrhizobium,* and *Rhizobium* strains selected for validation were isolated from. Coloured squares below the bar chart indicate the number of selected strains for each nodule. Square colours indicate the specific ASV these isolates belong to, as determined by whole genome sequencing. These colours correspond to the ASVs shown in Figure S9A.


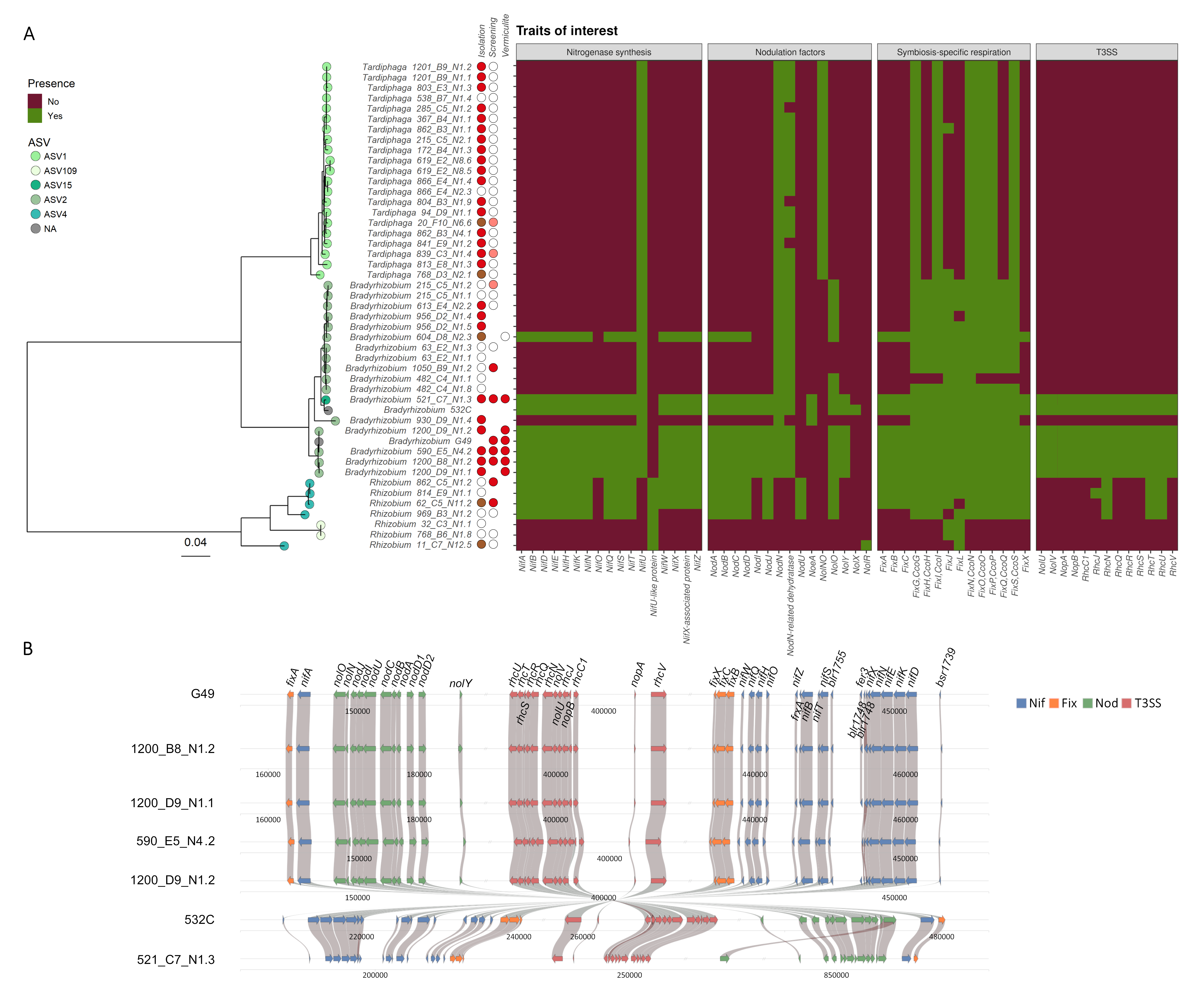


###### **Figure S9: Phylogeny of isolated strains and presence of the symbiotic genes.** (A) A phylogenetic tree based on a concatenated alignment of 107 conserved core genes was generated using bcgTree with partitioned maximum-likelihood analysis and 1,000 bootstrap replicates. The tree illustrates the phylogenetic relationship between isolated strains belonging to the genera *Tardiphaga*, *Bradyrhizobium,* or *Rhizobium*. Coloured dots indicate the ASV each strain belongs to. The heatmap represents the presence (green) or absence (dark red) of symbiotic genes in the genome of each strain. Symbiotic genes are grouped in the following classes: nitrogenase synthesis genes, nodulation factor synthesis and transport genes, symbiosis-specific respiration genes, and genes encoding components of the type 3-secretion system (T3SS). Coloured dots next to strain names indicate the colour of the nodule the strain was isolated from, and the colour of the nodules the strain could induce in pot trials (screening experiment: Fig. S10, vermiculite pot trial: Fig. 4C). Species assignments were validated using Average Nucleotide Identity (ANI) via the orthoANIu method. (B) Arrows representing the position of genes important for nodulation and nitrogen fixation inside the big element of the tRNA-Val symbiotic island. The blue-coloured arrows are the genes involved in nitrogenase synthesis, the orange-coloured arrows are symbiosis-specific respiration genes, the green-coloured arrows represent genes involved in nodulation factors synthesis, and the red-coloured arrows are the structural components of the T3SS. These gene cluster visualisation was generated with the *R* package geneviewer.


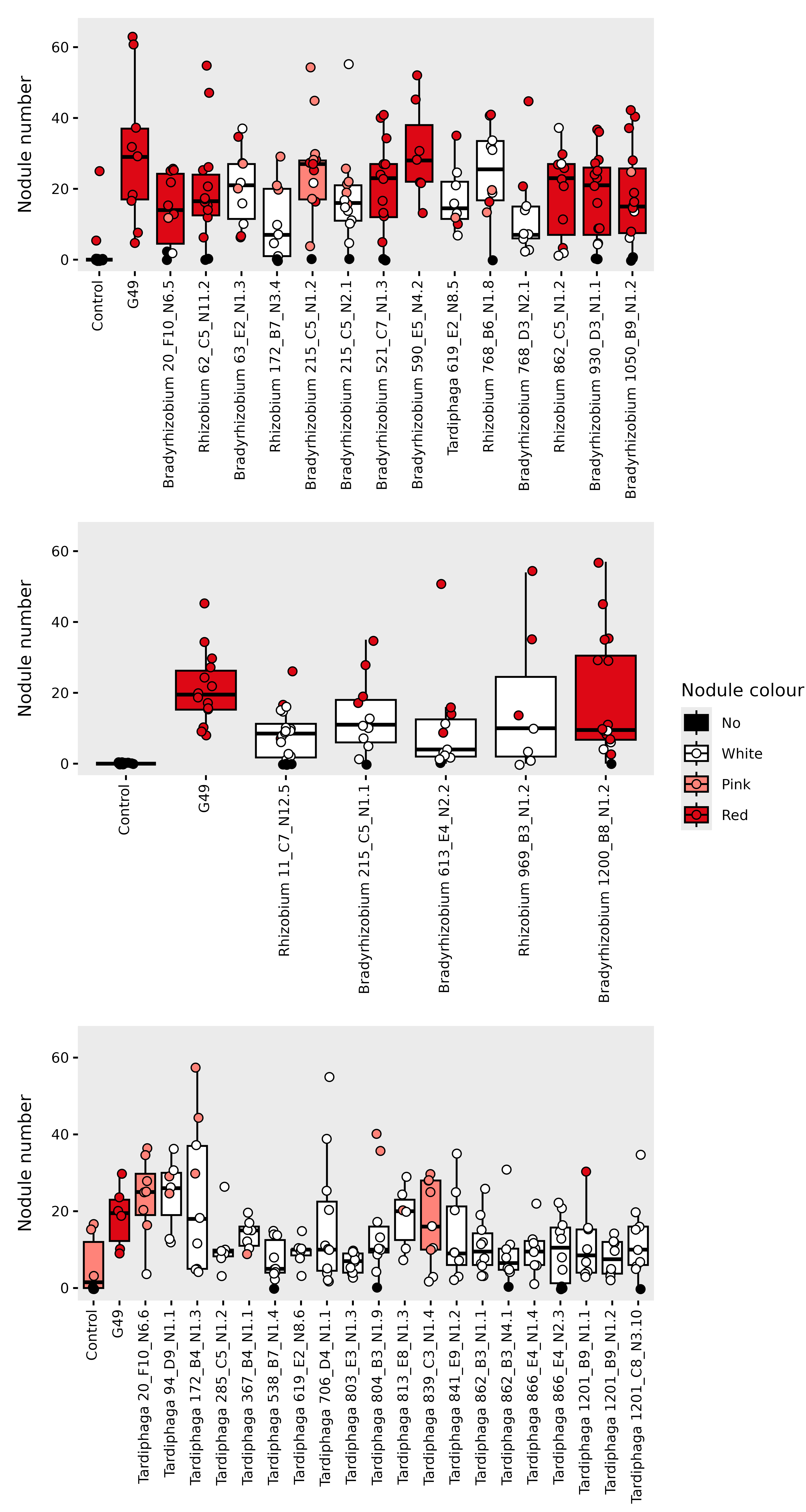


###### **Figure S10: Nodulation capacity of native isolated strains.** Soybean seeds of variety ’Acardia’ were inoculated with the indicated isolated strains and grown in sterile vermiculite. Seeds inoculated with *B. diazoefficiens* G49 or non-inoculated (Control) seeds were used as a positive or negative control, respectively. At four weeks post-inoculation, nodules were counted, and nodule colour was noted. For each treatment, at least six plants were analysed, with each plant being represented by a single dot. Treatments were grouped in three separate experiments, each displayed in a separate graph. Each dot represents the number of nodules on a single plant, with colour indicating the colour of the most active nodule found on that plant (red > pink > white). Plants without nodules are presented in black. Colours of the boxplots represent the most prevalent nodule colour across plants receiving the same treatment. The centre lines in the boxplots show the median, with the box limits representing the upper and lower quartiles, and the whiskers representing the maximum and minimum values without considering outliers defined as values outside 1.5x the interquartile range above or below the box.

**A
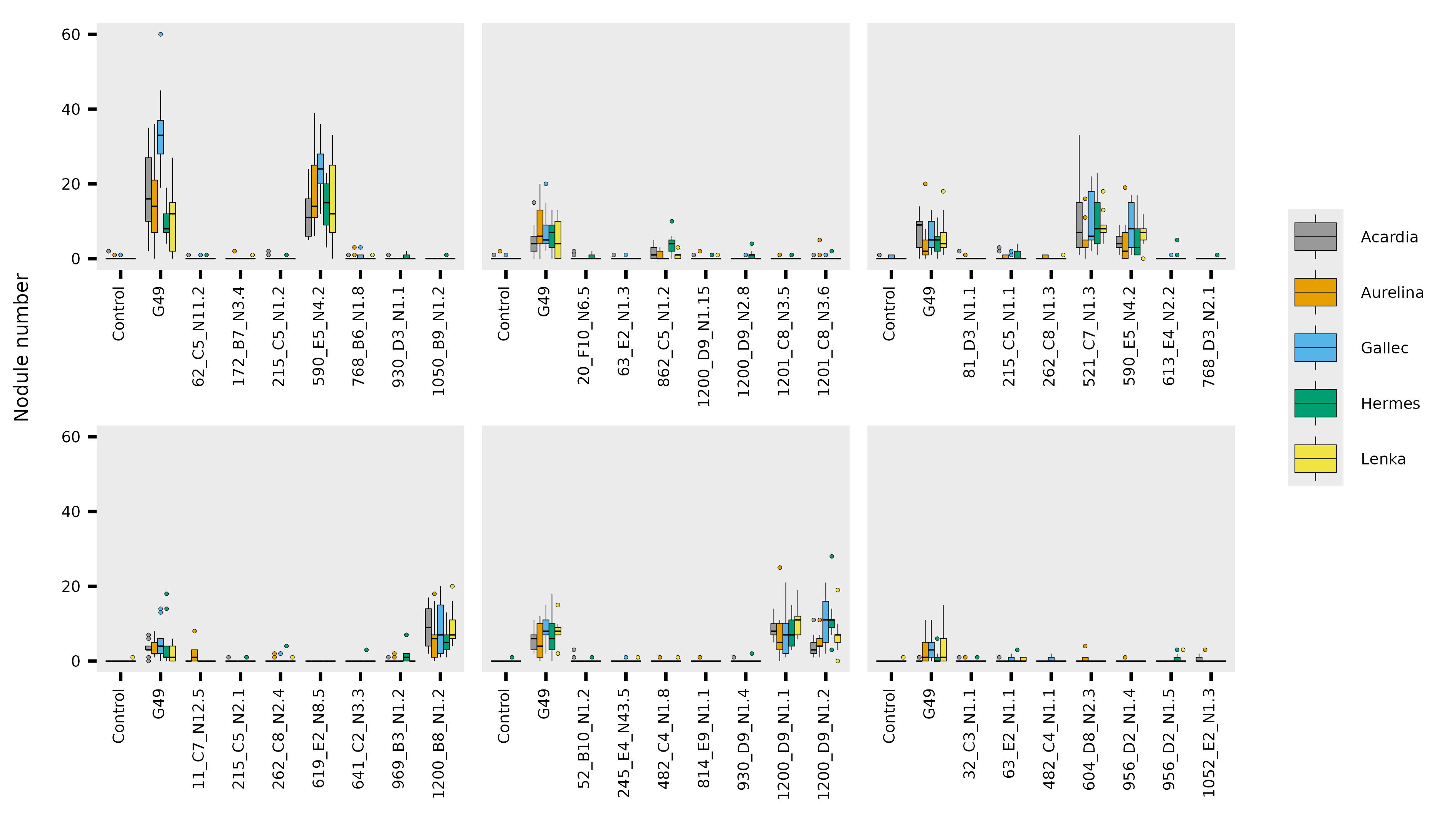
**

**B
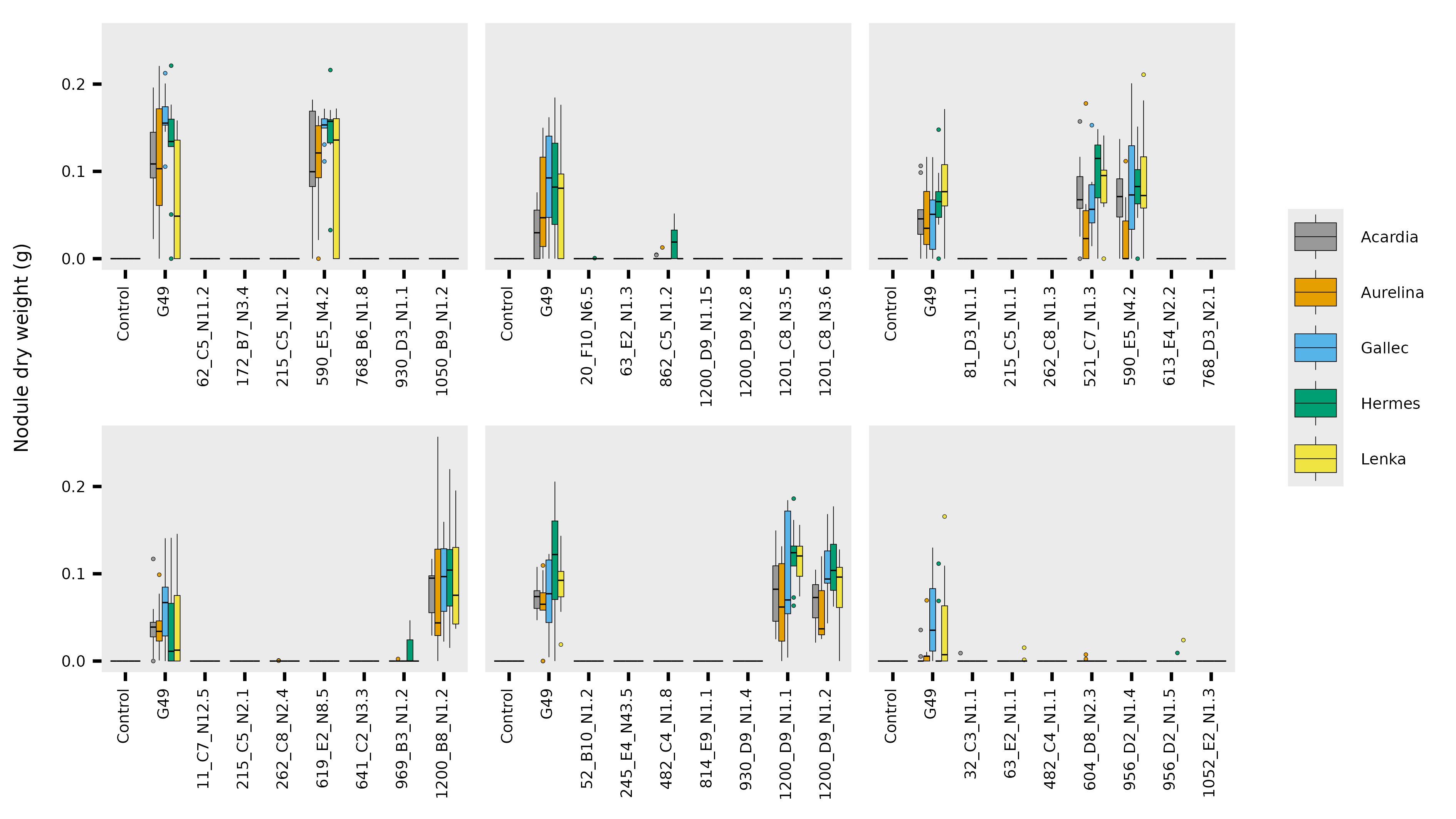
**

**C**

**
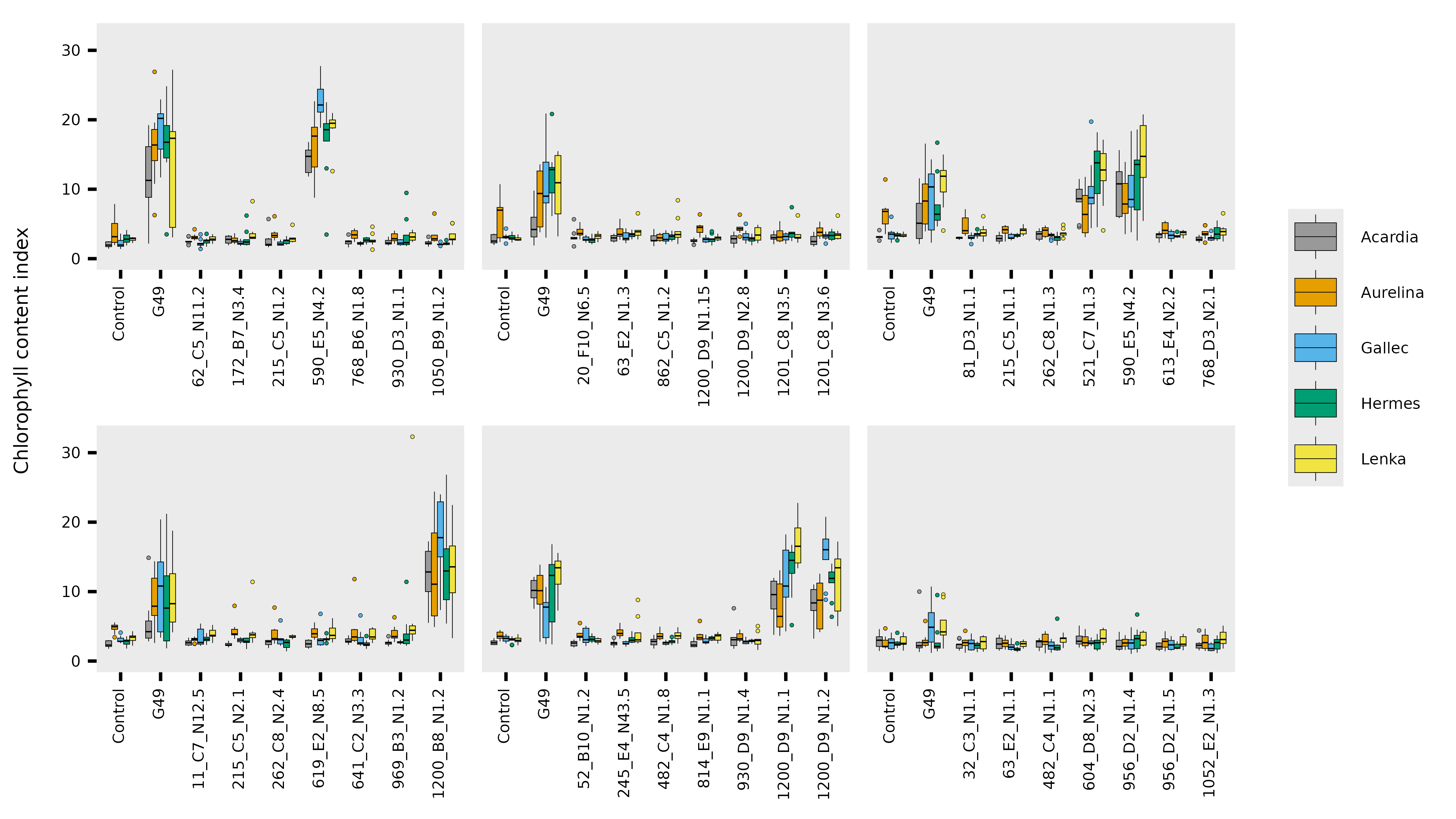
D**

**
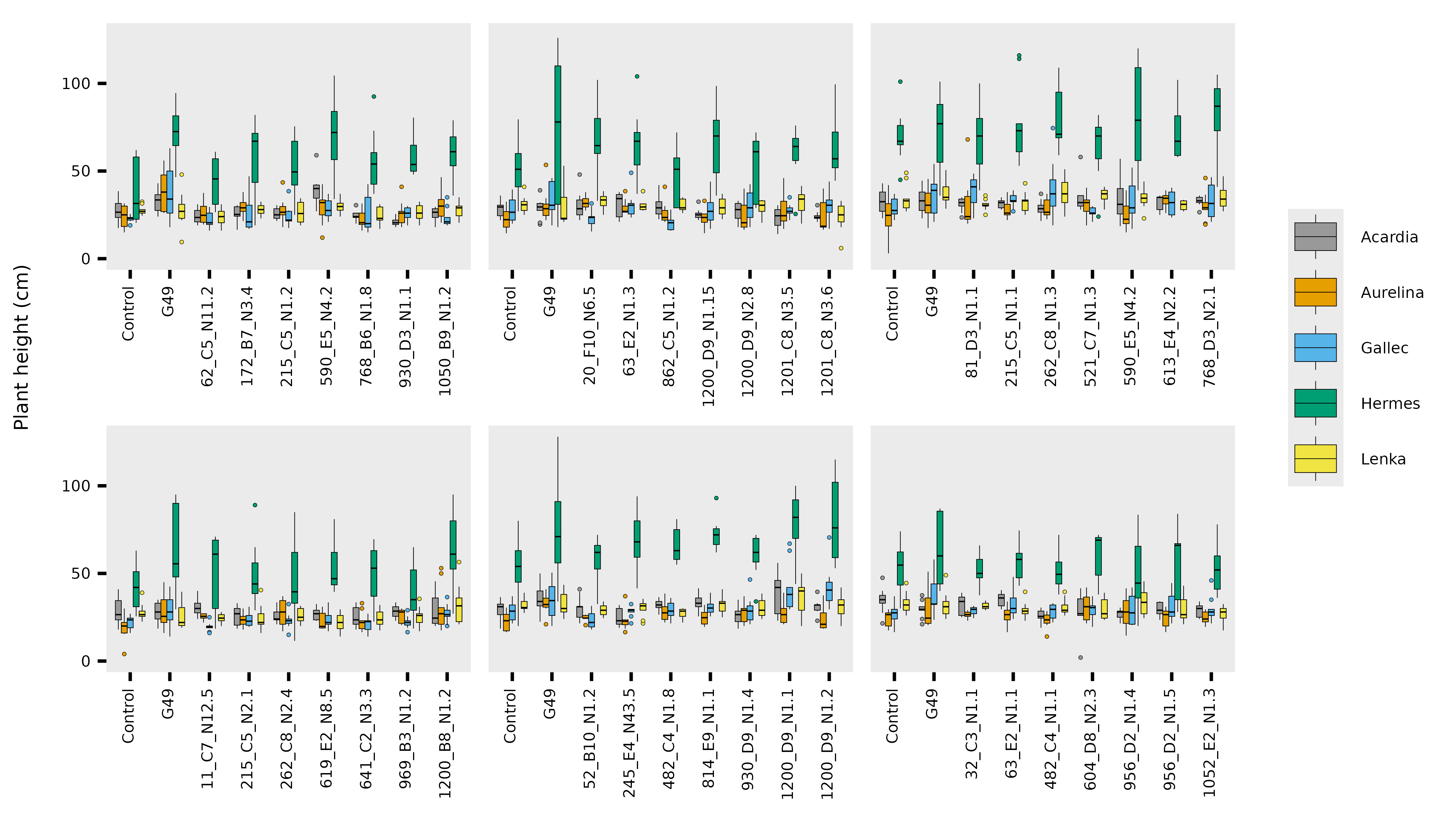
**

**E
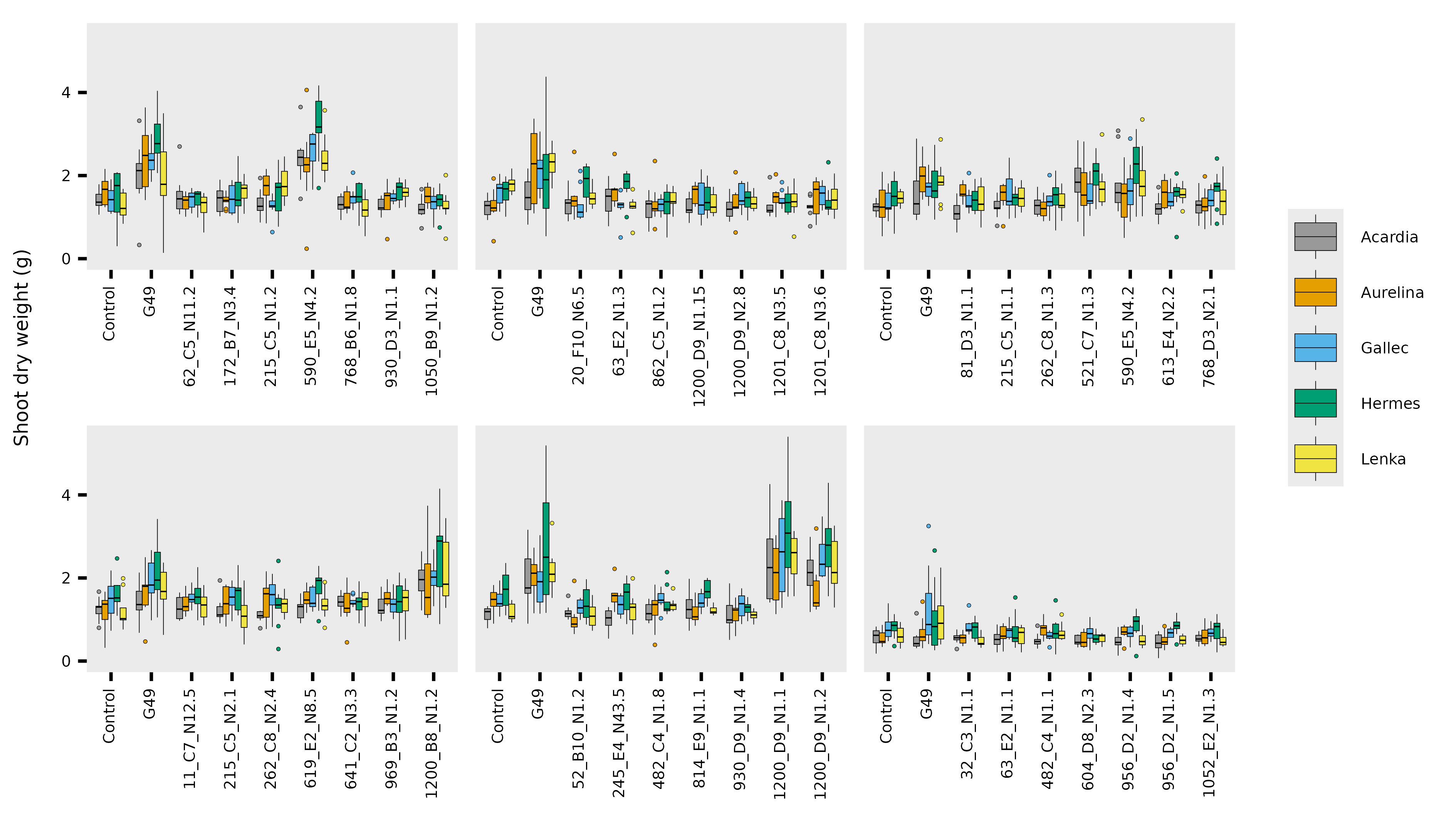
**

###### **Figure S11: Complete results of the pot trials in unsterilised soil.** Soybean seeds of 5 varieties were inoculated with the indicated isolated strains and grown in a 1:1 mixture of fresh agricultural soil and sand. Seeds inoculated with G49 or non-inoculated (Control) seeds were used as a positive or negative control, respectively. At eight weeks after sowing, nodule number (A), nodule dry weight (B), chlorophyll content (C), plant height (D), and shoot dry weight (E) were determined. For each treatment and variety, nine plants, grouped in three pots, were analysed. Treatments were grouped in six separate experiments, each displayed in a separate graph. The centre lines in the boxplots show the median, with the box limits representing the upper and lower quartiles, and the whiskers representing the maximum and minimum values without considering outliers defined as values outside 1.5x the interquartile range above or below the box. Boxplot colour represents the soybean variety.

######
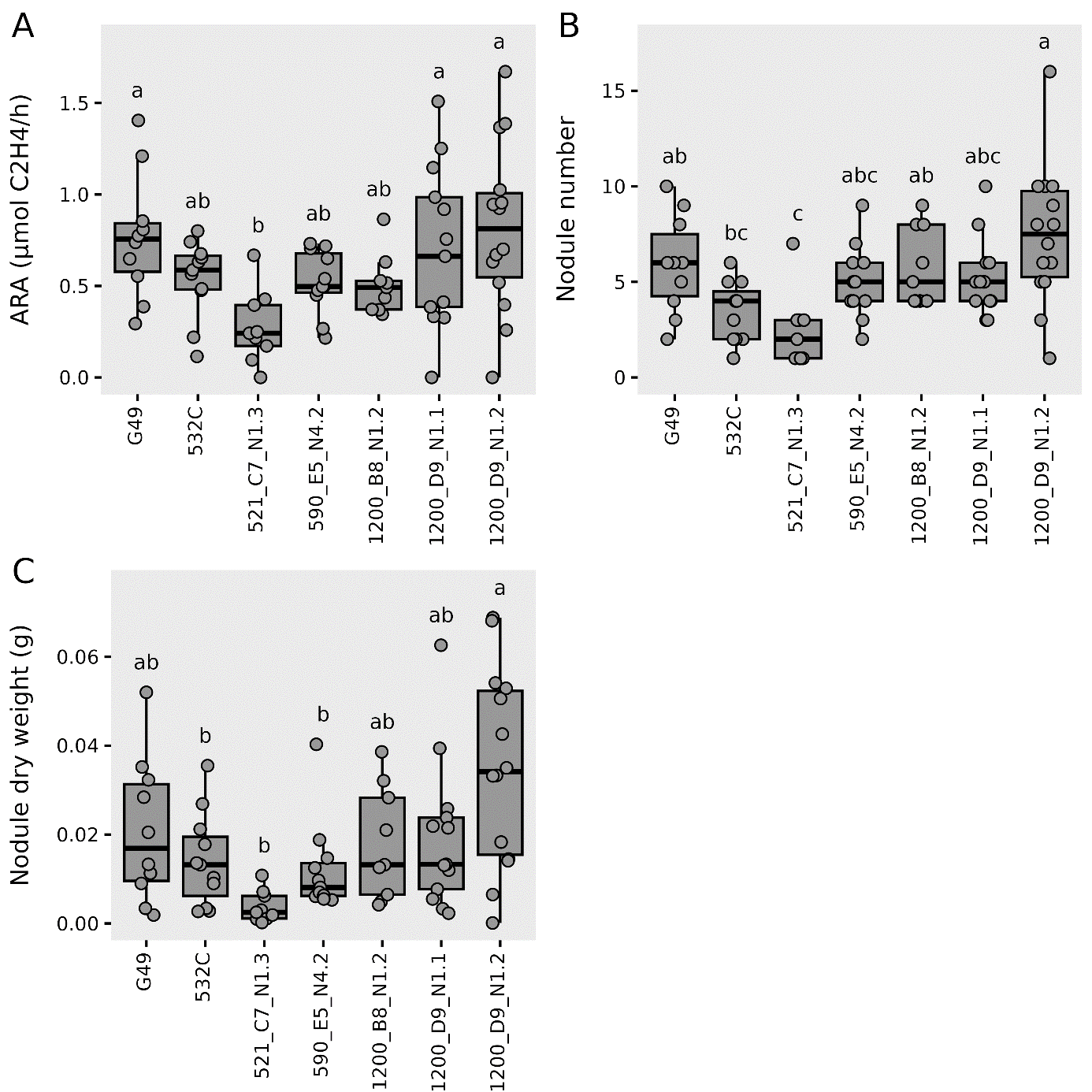
**Figure S12: Nitrogen fixation efficiency of selected trapped rhizobia strains.** Soybean seeds of variety ’Acardia’ were inoculated with the indicated isolated strains and grown in a 1:1 mixture of fresh agricultural soil and sand. Seeds inoculated with *Bradyrhizobium* strains G49 or 532C were used as a positive control. Nitrogenase activity (A) was determined at four weeks post-inoculation by measuring the acetylene reduction activity (ARA) for each root system. ARA is represented as μmol of ethylene (C_2_H_4_) produced per hour. Per root system, nodule number (B), and nodule dry weight (C) were also assessed. For each treatment, at least nine plants were analysed, with each plant being represented by a single dot. The centre lines in the boxplots show the median, with the box limits representing the upper and lower quartiles, and the whiskers representing the maximum and minimum values without considering outliers defined as values outside 1.5x the interquartile range above or below the box. Significant differences between treatments were determined using one-way ANOVA with Tukey multiple comparison correction. Letters indicate significance groups (*P* < 0.05). **A**


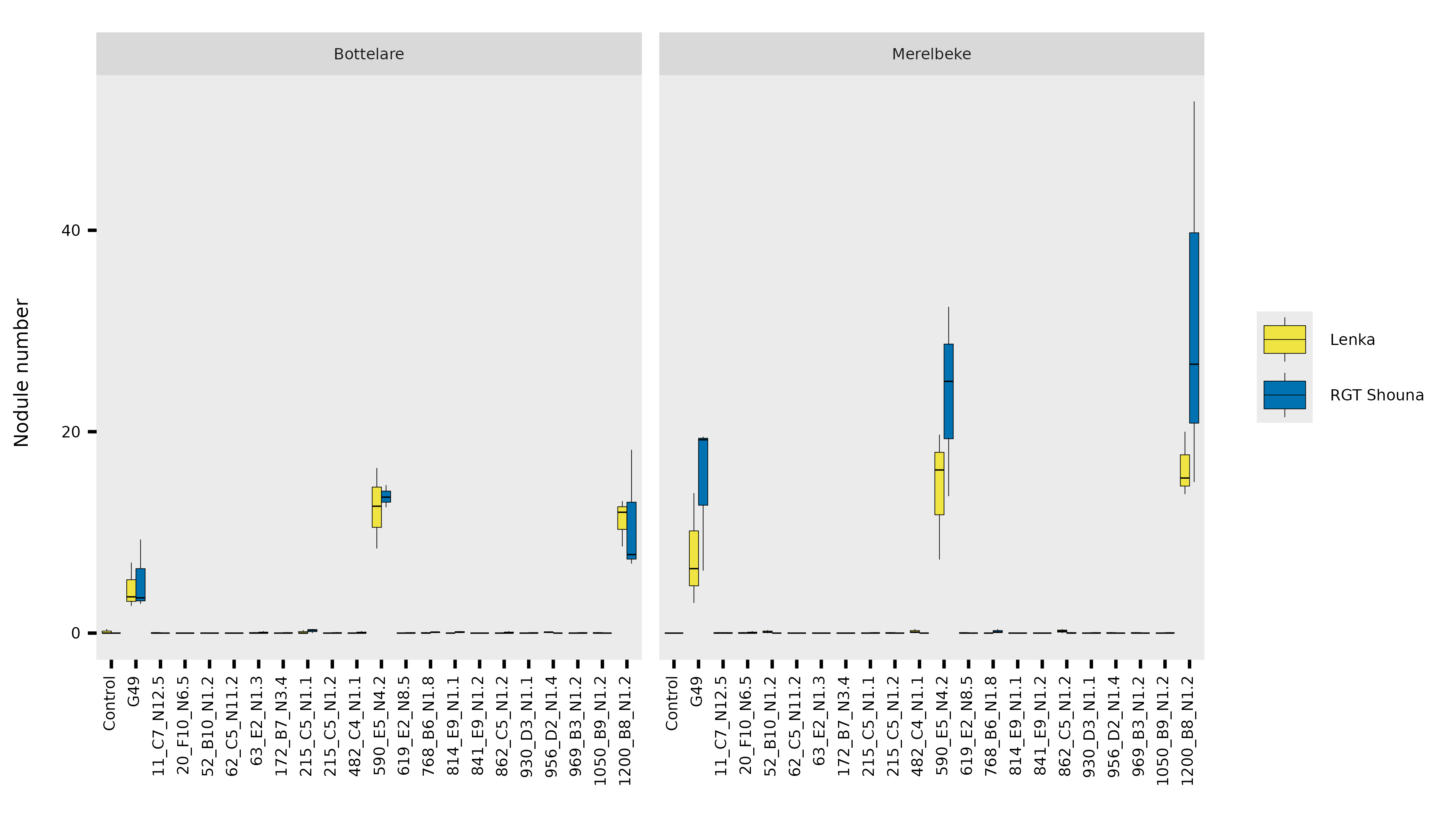
**B**


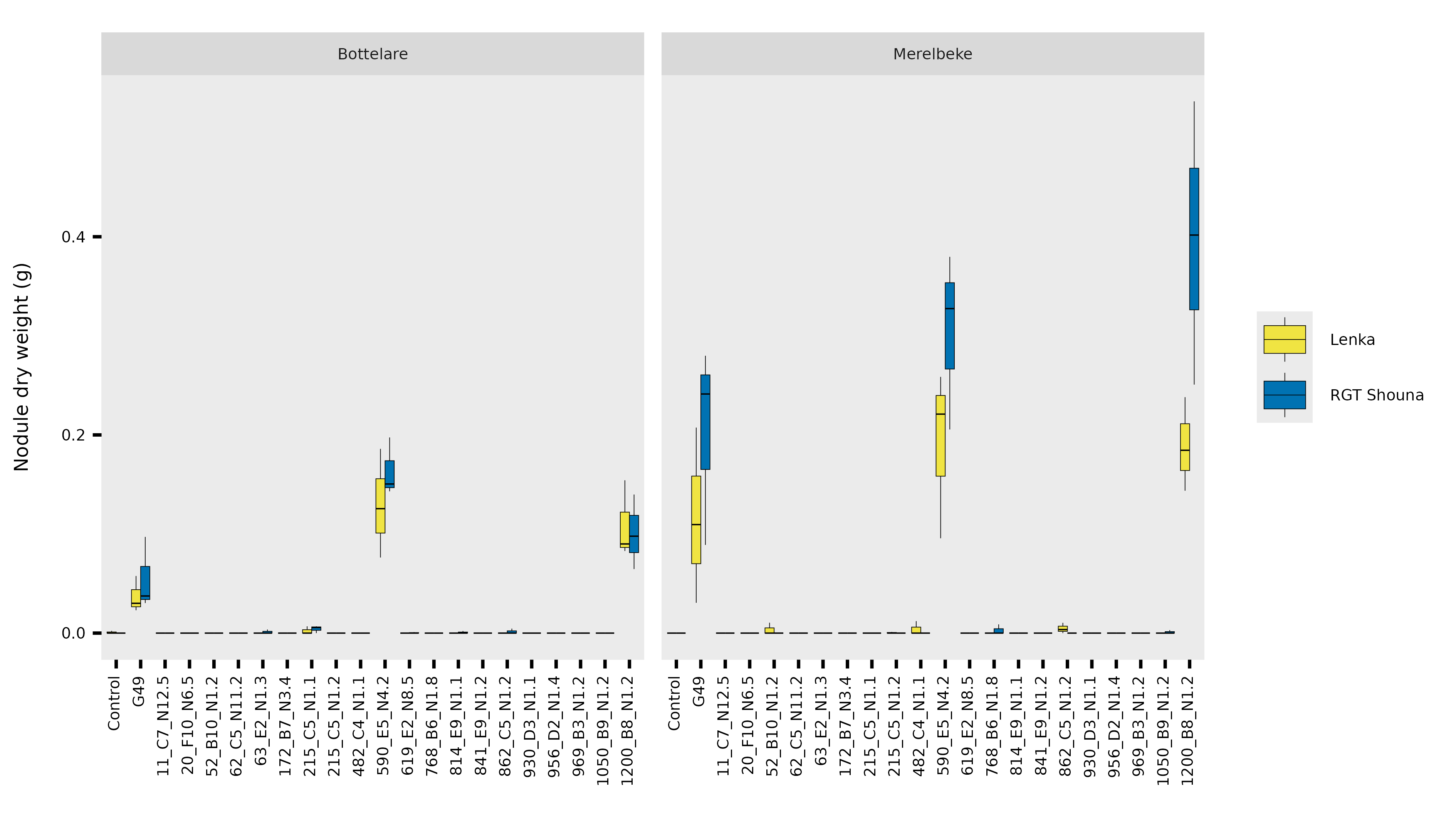


**C**
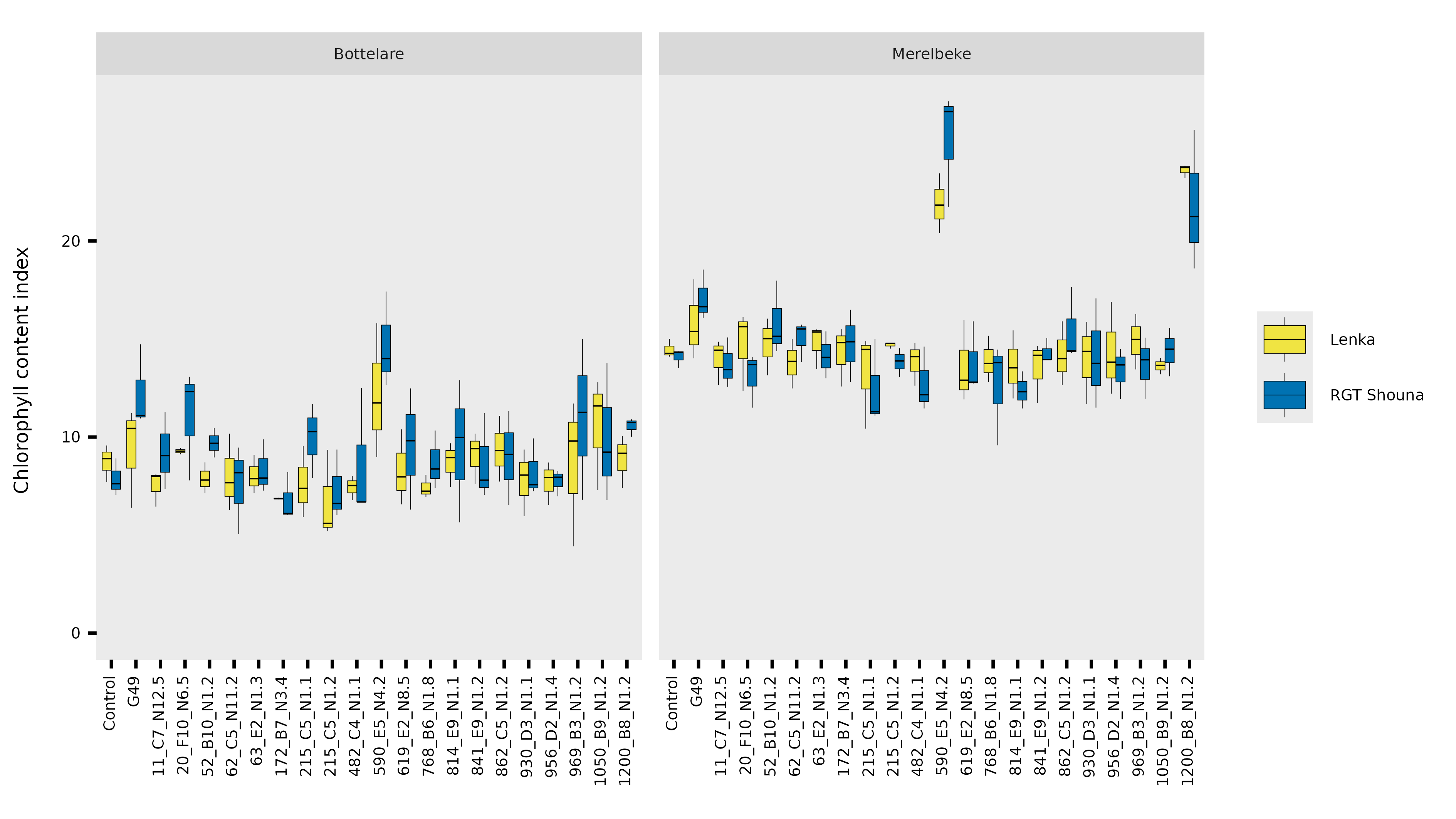


**D
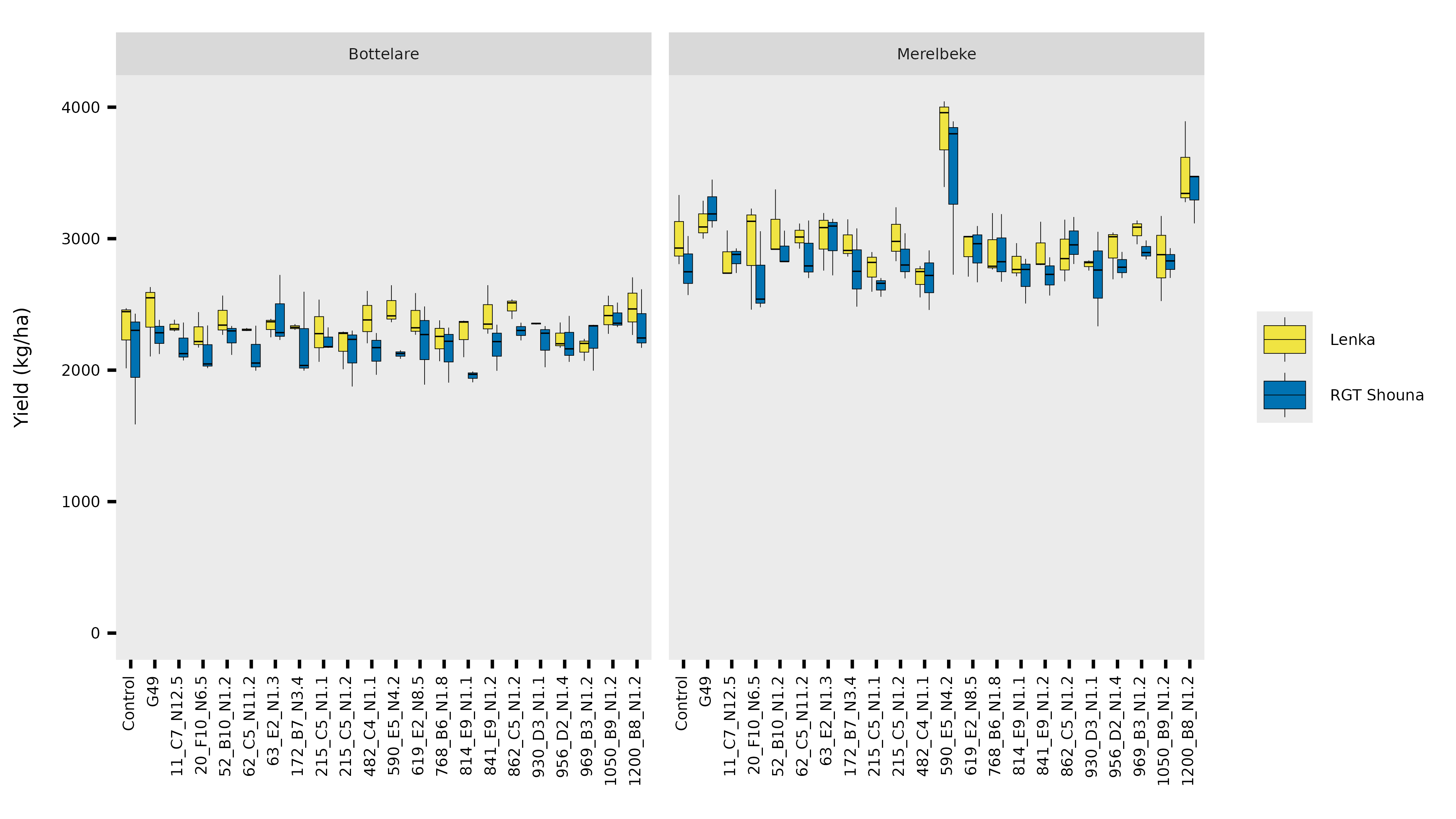
**

**E**
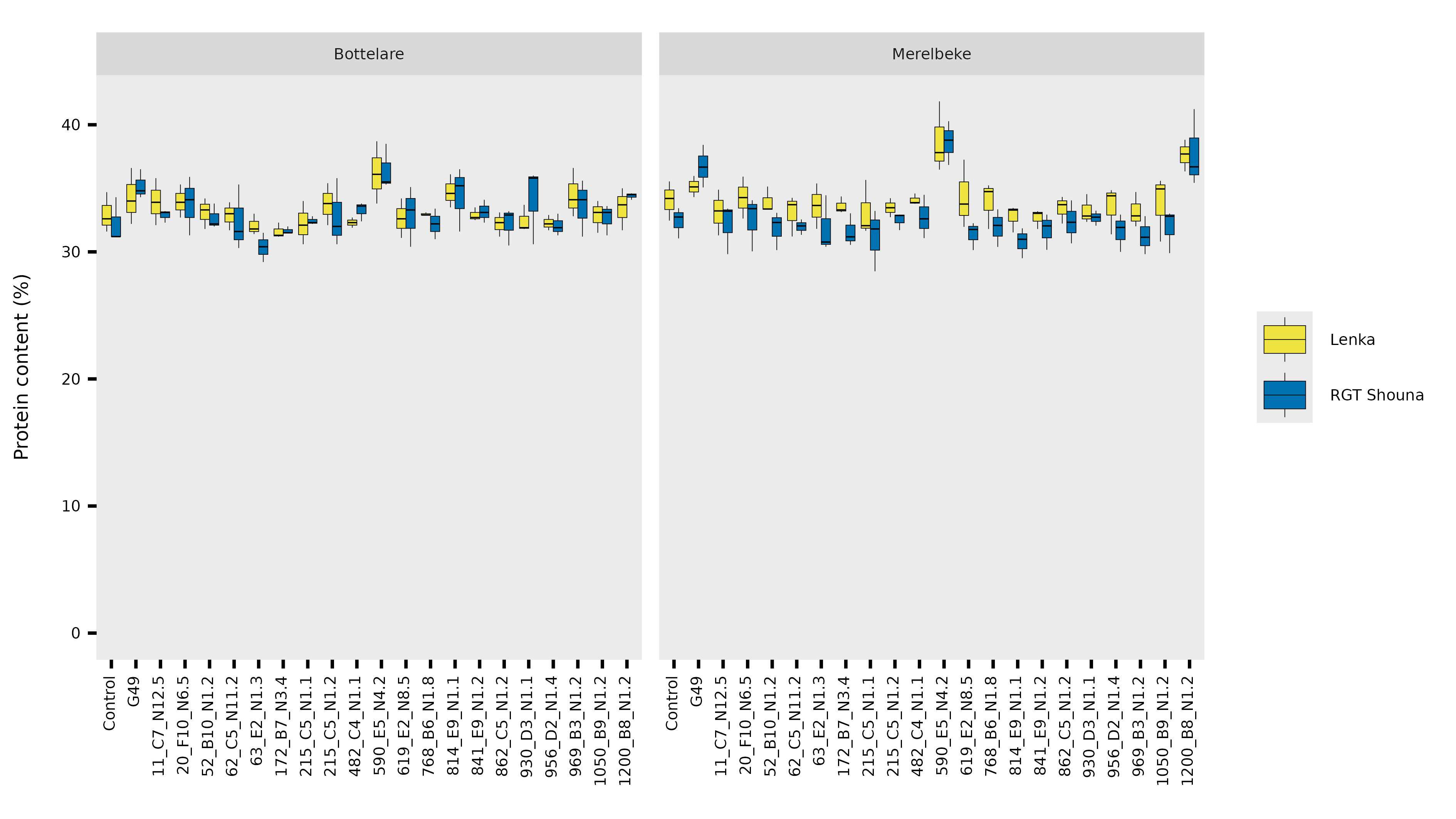
**F
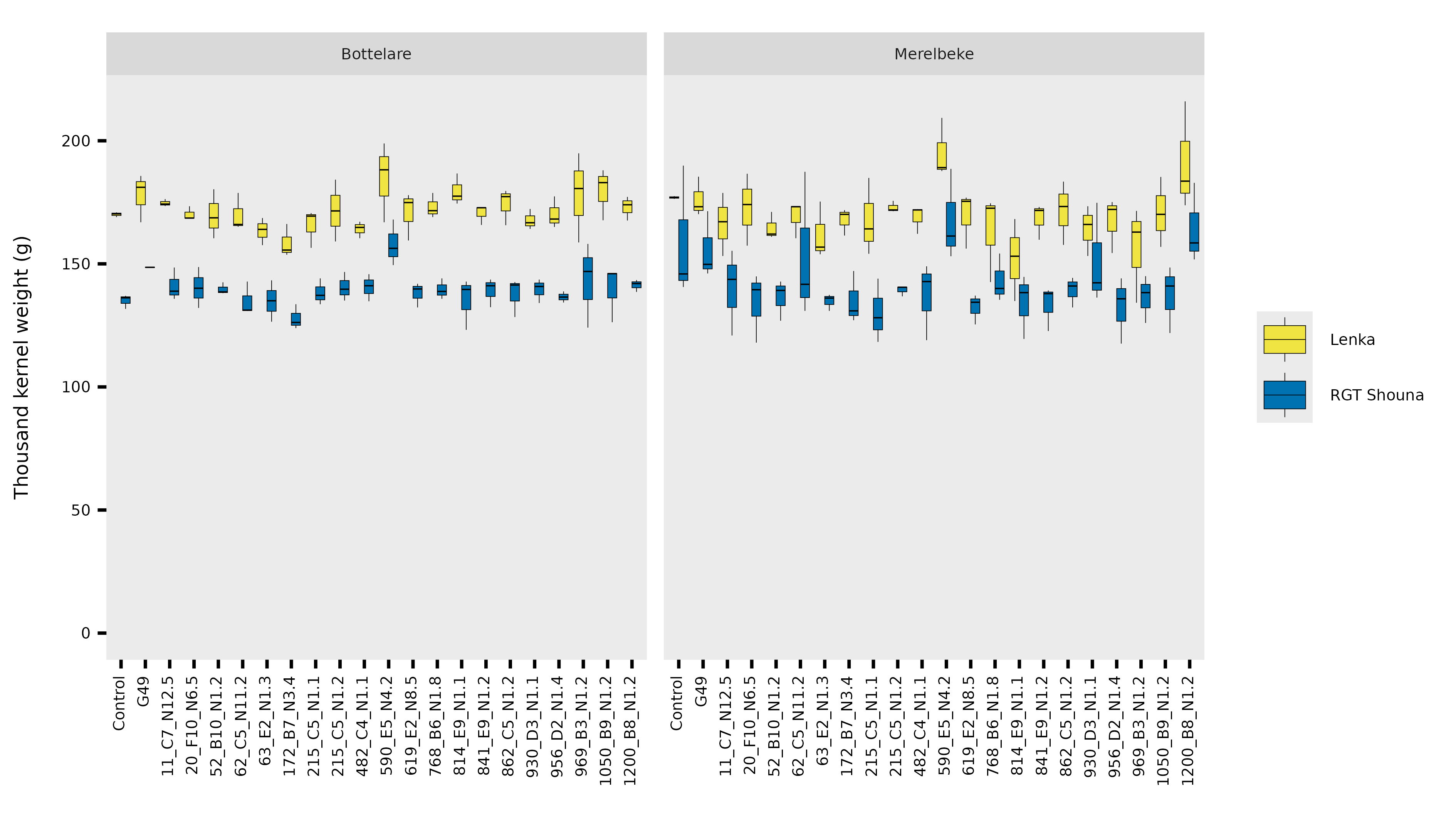
**

###### **Figure S13: Results of field trials.** The *Bradyrhizobium* strains 590_E5_N4.2 and 1200_B8_N1.2 were tested along a set of other strains. Soybean seeds of variety Lenka (yellow) and RGT Shouna (blue) were inoculated with the indicated isolated strains and sown in fields at two different locations (Bottelare and Merelbeke). Seeds inoculated with G49 or non-inoculated (Control) seeds were used as a positive or negative control, respectively. Nodule number (A), nodule dry weight (B), chlorophyll content (C), bean yield (D), bean protein content (E), and thousand kernel weight (F) were determined when plants reached growth stage R5 (A-C) or full maturity (D-F). For each treatment, variety, and location, three separate plots were analysed. Nodule and yield parameters were determined per plot, except for nodule number (A) and chlorophyll content (C), which were measured for five plants and ten leaves per plot, respectively. The centre lines in the boxplots show the median, with the box limits representing the upper and lower quartiles, and the whiskers representing the maximum and minimum value. Boxplot colour represents the soybean variety.


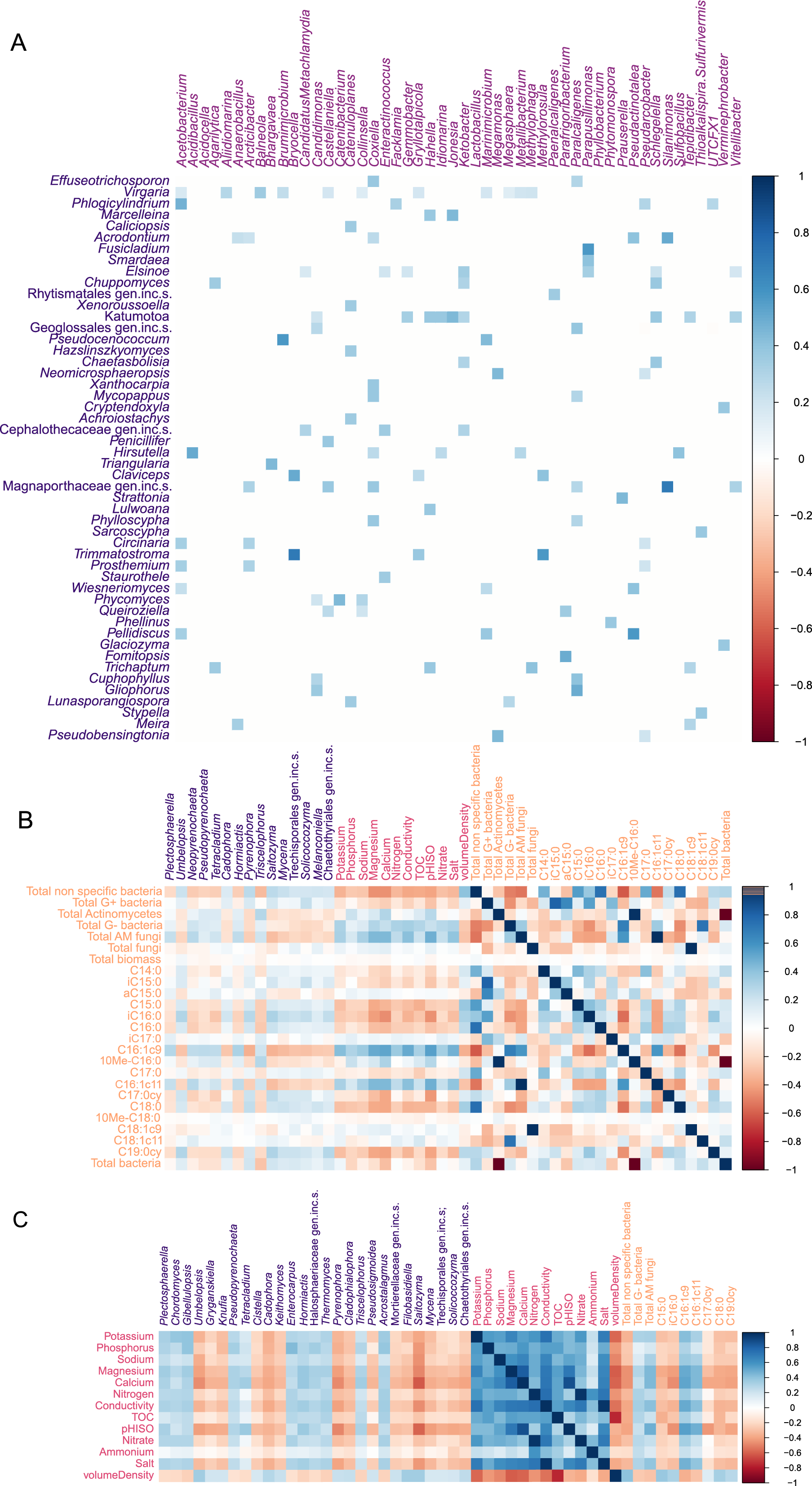


###### **Figure S14:** Spearman correlations between the ITS, 16S, PSC, and PLFA variables. (A) visualises correlations between the 50 most strongly correlated ITS (rows) and 16S (columns) variables. (B) shows the 50 variables (columns) that are most strongly correlated with the PLFA variables (rows) and (C) the 50 variables (columns) most strongly correlated with the PSC variables (rows).

**
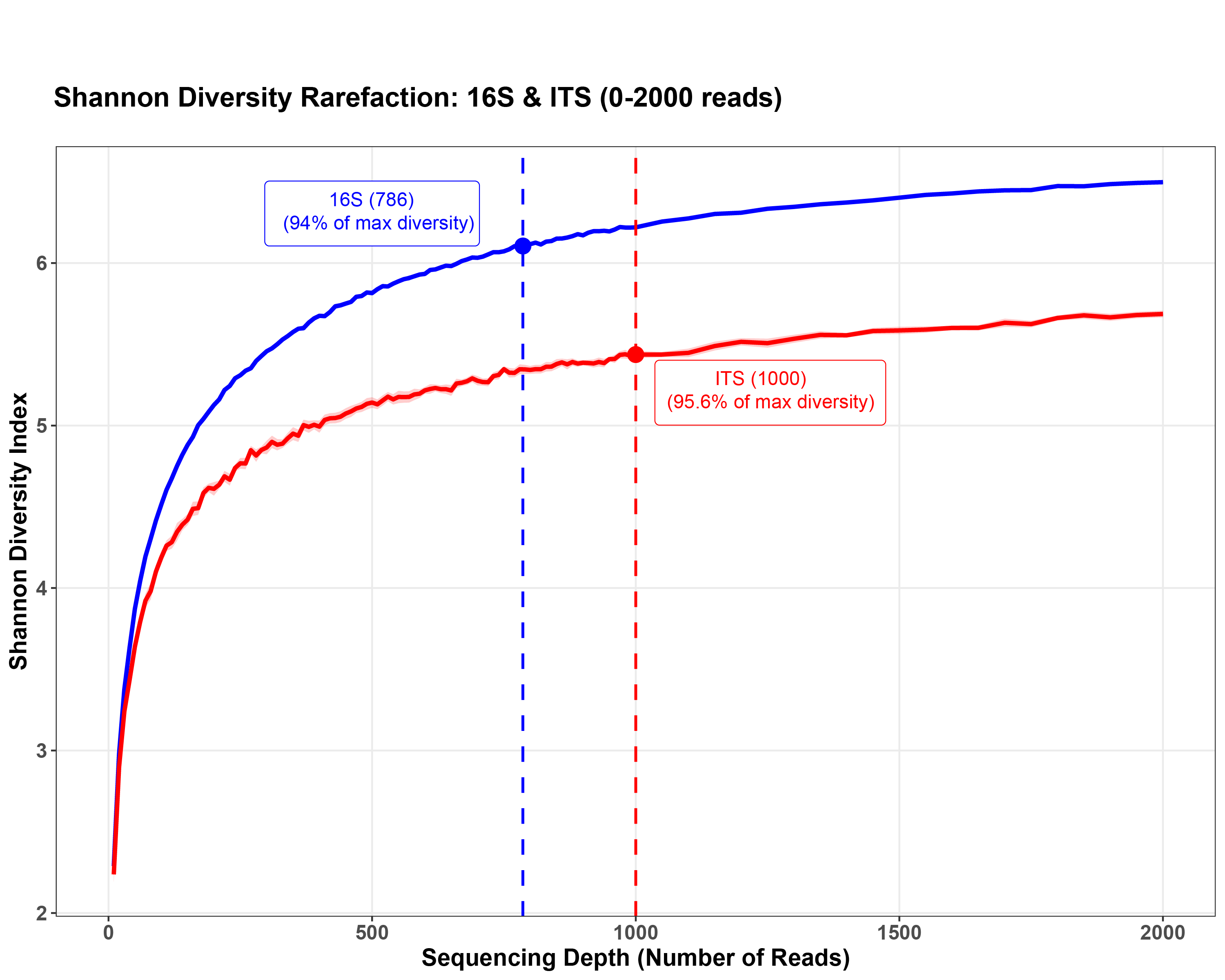
**

###### **Figure S15:** Shannon diversity rarefaction analysis for bacterial (16S rRNA gene, blue line) and fungal (ITS, red line) communities. Vertical dashed lines show chosen rarefaction depths capturing >94% of maximum diversity for both datasets (786 reads for 16S, 1000 reads for ITS).

# Supplementary Tables

###### Table S1: Table with information on the genome assembly and annotation, retrieved from the Bacterial and Viral Bioinformatics Resource Center (BV-BRC). It contains the number of contigs, the genome length, GC content, contig N50, contig L50, number of tRNA and rRNA genes, completeness, contamination (Contam.), the coding DNA sequences (CDS), and the number of repeated regions (Repeat) found in the genomes.

| Isolates | Contigs | Genome  length | GC (%) | N50 | L50 | tRNA | rRNA | Completeness (%) | Contam.  (%) | CDS | Hypothetical CDS | Repeat | PFAM CDS |
| --- | --- | --- | --- | --- | --- | --- | --- | --- | --- | --- | --- | --- | --- |
| *Bradyrhizobium* sp. | | | | | | | | | | | | | |
| 1050_B9_N1.2 | 6 | 9122251 | 63.74 | 8984645 | 1 | 55 | 6 | 98.5 | 3.5 | 9210 | 3803 | 117 | 8615 |
| 1200_B8_N1.2 | 1 | 9123846 | 63.98 | 9123846 | 1 | 56 | 3 | 99.8 | 2.1 | 9115 | 3701 | 170 | 8703 |
| 1200_D9_N1.1 | 1 | 9123100 | 63.98 | 9123100 | 1 | 50 | 3 | 99.8 | 2.1 | 9121 | 3706 | 170 | 8706 |
| 1200_D9_N1.2 | 1 | 9138826 | 63.95 | 9138826 | 1 | 50 | 3 | 99.8 | 2.5 | 9119 | 3681 | 155 | 8892 |
| 215_C5_N1.1 | 1 | 8838080 | 63.83 | 8838080 | 1 | 56 | 6 | 98.5 | 2.5 | 8920 | 3697 | 174 | 8273 |
| 215_C5_N1.2 | 1 | 8838080 | 63.83 | 8838080 | 1 | 56 | 6 | 98.5 | 2.5 | 8913 | 3688 | 174 | 8268 |
| 482_C4_N1.1 | 1 | 8969389 | 63.84 | 8969389 | 1 | 54 | 6 | 98.5 | 3.6 | 8883 | 3529 | 91 | 8346 |
| 482_C4_N1.8 | 1 | 8966771 | 63.84 | 8966771 | 1 | 54 | 6 | 98.5 | 3.6 | 8885 | 3533 | 91 | 8347 |
| 521_C7_N1.3 | 1 | 10214093 | 63.35 | 10214093 | 1 | 56 | 6 | 99.9 | 3.6 | 10420 | 4448 | 151 | 9407 |
| 590_E5_N4.2 | 1 | 9135623 | 63.95 | 9135623 | 1 | 50 | 3 | 99.8 | 2.4 | 9105 | 3674 | 153 | 8880 |
| 604_D8_N2.3 | 2 | 9708622 | 63.51 | 9305757 | 1 | 57 | 6 | 99.8 | 3 | 10074 | 4334 | 233 | 9053 |
| 613_E4_N2.2 | 1 | 8954086 | 63.87 | 8954086 | 1 | 53 | 6 | 98.5 | 3.1 | 8856 | 3554 | 109 | 8415 |
| 63_E2_N1.1 | 1 | 8934234 | 63.80 | 8934234 | 1 | 53 | 6 | 98.5 | 2.8 | 8924 | 3586 | 112 | 8543 |
| 63_E2_N1.3 | 1 | 8918488 | 63.81 | 8918488 | 1 | 53 | 6 | 98.5 | 2.8 | 8896 | 8527 | 112 | 8527 |
| 930_D9_N1.4 | 1 | 7352897 | 64.25 | 7352897 | 1 | 50 | 3 | 97.7 | 2 | 7157 | 2647 | 60 | 6448 |
| 956_D2_N1.4 | 1 | 9236448 | 63.68 | 9236448 | 1 | 54 | 6 | 98.5 | 3.1 | 9246 | 3820 | 132 | 8507 |
| 956_D2_N1_5 | 2 | 9235669 | 63.68 | 6122751 | 1 | 52 | 6 | 98.5 | 3.1 | 9241 | 3814 | 132 | 8497 |
| *Rhizobium* sp. | | | | | | | | | | | | | |
| 11_C7_N12.5 | 4 | 7609534 | 59.71 | 3914882 | 1 | 53 | 9 | 100 | 0.5 | 7658 | 2333 | 94 | 6972 |
| 32_C3_N1.1 | 5 | 7906496 | 59.91 | 4824156 | 1 | 54 | 9 | 99.8 | 0.2 | 8042 | 2578 | 60 | 6876 |
| 62_C5_N11.2 | 6 | 7371528 | 60.59 | 4850909 | 1 | 53 | 9 | 100 | 0.2 | 7458 | 2190 | 163 | 7048 |
| 768_B6_N1.8 | 5 | 7906496 | 59.91 | 4824156 | 1 | 54 | 9 | 97.7 | 2 | 8046 | 2583 | 60 | 6876 |
| 814_E9_N1.1 | 6 | 8068231 | 60.61 | 5039705 | 1 | 52 | 9 | 100 | 0 | 8231 | 2484 | 158 | 7656 |
| 862_C5_N1.2 | 6 | 7984548 | 60.60 | 4954050 | 1 | 51 | 9 | 100 | 0 | 8091 | 2378 | 157 | 7555 |
| 969_B3_N1.2 | 5 | 6418066 | 61.29 | 4607491 | 1 | 51 | 9 | 100 | 0 | 6400 | 1741 | 69 | 6122 |
| *Tardiphaga* sp. | | | | | | | | | | | | | |
| 1201_B9_N1.1 | 3 | 6397858 | 61.53 | 6283603 | 1 | 50 | 6 | 100 | 0.5 | 6232 | 2207 | 37 | 5421 |
| 1201_B9_N1.2 | 4 | 6381180 | 61.53 | 3200188 | 1 | 50 | 6 | 100 | 0.5 | 6208 | 2196 | 37 | 5398 |
| 172_B4_N1.3 | 1 | 6296309 | 61.60 | 6296309 | 1 | 50 | 6 | 100 | 0 | 6109 | 2201 | 40 | 5481 |
| 20_F10_N6.6 | 1 | 6448917 | 61.43 | 6448917 | 1 | 49 | 6 | 100 | 0.2 | 6364 | 2370 | 42 | 5492 |
| 215_C5_N2.1 | 1 | 6603980 | 61.42 | 6603980 | 1 | 49 | 6 | 100 | 0 | 6446 | 2363 | 32 | 5565 |
| 285_C5_N1.2 | 1 | 6228377 | 61.51 | 6228377 | 1 | 50 | 6 | 100 | 0 | 6065 | 2160 | 32 | 5377 |
| 367_B4_N1_1 | 3 | 6684679 | 61.43 | 6568205 | 1 | 51 | 6 | 100 | 0.5 | 6587 | 2456 | 40 | 5564 |
| 538_B7_N1.4 | 3 | 6633743 | 61.38 | 6447102 | 1 | 49 | 6 | 100 | 0.2 | 6562 | 2446 | 40 | 5481 |
| 619_E2_N8.5 | 3 | 6309736 | 61.49 | 6221213 | 1 | 49 | 6 | 100 | 0.2 | 6148 | 2198 | 33 | 5427 |
| 619_E2_N8.6 | 2 | 6309627 | 61.49 | 6252423 | 1 | 49 | 6 | 100 | 0.2 | 6155 | 2207 | 33 | 5420 |
| 768_D3_N2.1 | 1 | 6083462 | 61.65 | 6083462 | 1 | 49 | 6 | 99.5 | 0 | 5875 | 2042 | 21 | 5050 |
| 803_E3_N1.3 | 3 | 6235009 | 61.58 | 4831608 | 1 | 51 | 6 | 100 | 0.7 | 6070 | 2106 | 33 | 5394 |
| 804_B3_N1.9 | 2 | 6141180 | 61.55 | 5920151 | 1 | 49 | 6 | 100 | 0 | 5966 | 2085 | 32 | 5333 |
| 813_E8_N1.3 | 2 | 6069818 | 61.48 | 6013354 | 1 | 50 | 6 | 100 | 0.2 | 5882 | 2036 | 26 | 5118 |
| 839_C3_N1.4 | 2 | 6359327 | 61.47 | 6217173 | 1 | 51 | 6 | 100 | 0.2 | 6291 | 2412 | 28 | 5281 |
| 841_E9_N1.2 | 4 | 6435215 | 61.51 | 6283309 | 1 | 49 | 6 | 100 | 0 | 6348 | 2385 | 36 | 5477 |
| 862_B3_N1.1 | 1 | 6630561 | 61.35 | 6630561 | 1 | 53 | 6 | 100 | 0.2 | 6552 | 2520 | 37 | 5458 |
| 862_B3_N4.1 | 3 | 6492189 | 61.42 | 3626430 | 1 | 48 | 6 | 100 | 0 | 6371 | 2322 | 34 | 5534 |
| 866_E4_N1.4 | 1 | 6913662 | 61.33 | 6913662 | 1 | 49 | 6 | 100 | 0,2 | 6856 | 2683 | 53 | 5533 |
| 866_E4_N2.3 | 2 | 6913201 | 61.33 | 6351592 | 1 | 49 | 6 | 100 | 0,2 | 6852 | 2678 | 51 | 5532 |
| 94_D9_N1.1 | 2 | 6422742 | 61.47 | 3737309 | 1 | 49 | 6 | 99.8 | 0 | 6268 | 2271 | 42 | 5508 |

###### **Table S2:** Comparison of the number of red nodules between plants grown in vermiculite and inoculated with promising strains or with a Control treatment. The results of One-way ANOVA tests with Tukey multiple comparison correction are summarised in this table.

| **Group 1** | **Group 2** | **n_1_** | **n_2_** | **Mean 1** | **Mean 2** | **Mean diff** | **SE** | **df** | **q** | ***P* value** |
| --- | --- | --- | --- | --- | --- | --- | --- | --- | --- | --- |
| 590_E5_N4.2 | 1200_B8_N1.2 | 12 | 12 | 44.5 | 48.5 | -4 | 5.764 | 88 | 0.9813 | 0.997 |
| 590_E5_N4.2 | 1200_D9_N1.2 | 12 | 12 | 44.5 | 45.25 | -0.75 | 5.764 | 88 | 0.184 | >0.9999 |
| 590_E5_N4.2 | 1200_D9_N1.1 | 12 | 12 | 44.5 | 63.83 | -19.33 | 5.764 | 88 | 4.743 | 0.0249 |
| 590_E5_N4.2 | 521_C7_N1.3 | 12 | 12 | 44.5 | 45.92 | -1.417 | 5.764 | 88 | 0.3476 | >0.9999 |
| 590_E5_N4.2 | 604_D8_N2.3 | 12 | 12 | 44.5 | 7.167 | 37.33 | 5.764 | 88 | 9.159 | <0.0001 |
| 590_E5_N4.2 | G49 | 12 | 12 | 44.5 | 56 | -11.5 | 5.764 | 88 | 2.821 | 0.4911 |
| 590_E5_N4.2 | Control | 12 | 12 | 44.5 | 1 | 43.5 | 5.764 | 88 | 10.67 | <0.0001 |
| 1200_B8_N1.2 | 1200_D9_N1.2 | 12 | 12 | 48.5 | 45.25 | 3.25 | 5.764 | 88 | 0.7973 | 0.9992 |
| 1200_B8_N1.2 | 1200_D9_N1.1 | 12 | 12 | 48.5 | 63.83 | -15.33 | 5.764 | 88 | 3.762 | 0.1492 |
| 1200_B8_N1.2 | 521_C7_N1.3 | 12 | 12 | 48.5 | 45.92 | 2.583 | 5.764 | 88 | 0.6338 | 0.9998 |
| 1200_B8_N1.2 | 604_D8_N2.3 | 12 | 12 | 48.5 | 7.167 | 41.33 | 5.764 | 88 | 10.14 | <0.0001 |
| 1200_B8_N1.2 | G49 | 12 | 12 | 48.5 | 56 | -7.5 | 5.764 | 88 | 1.84 | 0.8961 |
| 1200_B8_N1.2 | Control | 12 | 12 | 48.5 | 1 | 47.5 | 5.764 | 88 | 11.65 | <0.0001 |
| 1200_D9_N1.2 | 1200_D9_N1.1 | 12 | 12 | 45.25 | 63.83 | -18.58 | 5.764 | 88 | 4.559 | 0.0361 |
| 1200_D9_N1.2 | 521_C7_N1.3 | 12 | 12 | 45.25 | 45.92 | -0.6667 | 5.764 | 88 | 0.1636 | >0.9999 |
| 1200_D9_N1.2 | 604_D8_N2.3 | 12 | 12 | 45.25 | 7.167 | 38.08 | 5.764 | 88 | 9.343 | <0.0001 |
| 1200_D9_N1.2 | G49 | 12 | 12 | 45.25 | 56 | -10.75 | 5.764 | 88 | 2.637 | 0.5783 |
| 1200_D9_N1.2 | Control | 12 | 12 | 45.25 | 1 | 44.25 | 5.764 | 88 | 10.86 | <0.0001 |
| 1200_D9_N1.1 | 521_C7_N1.3 | 12 | 12 | 63.83 | 45.92 | 17.92 | 5.764 | 88 | 4.396 | 0.0496 |
| 1200_D9_N1.1 | 604_D8_N2.3 | 12 | 12 | 63.83 | 7.167 | 56.67 | 5.764 | 88 | 13.9 | <0.0001 |
| 1200_D9_N1.1 | G49 | 12 | 12 | 63.83 | 56 | 7.833 | 5.764 | 88 | 1.922 | 0.873 |
| 1200_D9_N1.1 | Control | 12 | 12 | 63.83 | 1 | 62.83 | 5.764 | 88 | 15.42 | <0.0001 |
| 521_C7_N1.3 | 604_D8_N2.3 | 12 | 12 | 45.92 | 7.167 | 38.75 | 5.764 | 88 | 9.507 | <0.0001 |
| 521_C7_N1.3 | G49 | 12 | 12 | 45.92 | 56 | -10.08 | 5.764 | 88 | 2.474 | 0.6553 |
| 521_C7_N1.3 | Control | 12 | 12 | 45.92 | 1 | 44.92 | 5.764 | 88 | 11.02 | <0.0001 |
| 604_D8_N2.3 | G49 | 12 | 12 | 7.167 | 56 | -48.83 | 5.764 | 88 | 11.98 | <0.0001 |
| 604_D8_N2.3 | Control | 12 | 12 | 7.167 | 1 | 6.167 | 5.764 | 88 | 1.513 | 0.9614 |
| G49 | Control | 12 | 12 | 56 | 1 | 55 | 5.764 | 88 | 13.49 | <0.0001 |

###### **Table S3:** Detailed descriptions of soil characteristics, fertilizer, and sowing and harvesting dates of the fields used in the field trials.

|  | **Merelbeke** | **Bottelare** |
| --- | --- | --- |
| Sowing date | 5/05/2022 | 2/05/2022 |
| Harvest date | 5/10/2022 | 23/09/2022 |
| Preceding crop | Fibre flax | Fibre flax |
| Fertilisation | 80 kg K/ha | 80 kg K/ha |
| Soil pH-KCl | 5.8 | 6.0 |
| NO_3_-N (kg/ha)^1^ | 7.1 | 6.0 |
| NH_4_-N (kg/ha)^1^ | 6.3 | 7.4 |

^1^ *Soil layer 0-30 cm*

###### **Table S4:** NLPO AUC and 95% confidence interval of the multi-variable models. div = 16S Shannon index.

| **Datasets** | **eln-log (95% CI)** | **pls-log (95% CI)** | **RF (95% CI)** |
| --- | --- | --- | --- |
| PSC | 0.654 (0.631-0.676) | 0.628 (0.605-0.652) | 0.586 (0.563-0.609) |
| ITS | 0.557 (0.533-0.580) | 0.593 (0.570-0.616) | 0.584 (0.561-0.607) |
| PLFA | 0.560 (0.558-0.562) | 0.552 (0.528-0.576) | 0.532 (0.510-0.555) |
| 16S | 0.467 (0.444-0.490) | 0.491 (0.467-0.514) | 0.487 (0.464-0.511) |
| PSC+div | 0.658 (0.636-0.681) | 0.639 (0.617-0.662) | 0.595 (0.572-0.619) |
| PSC+PLFA+div | 0.627 (0.604-0.650) | 0.607 (0.584-0.630) | 0.600 (0.577-0.623) |
| PSC+PLFA+ITS+div | 0.593 (0.570-0.616) | 0.620 (0.598-0.643) | 0.615 (0.592-0.638) |
| All+div | 0.599 (0.576-0.622) | 0.581 (0.557-0.604) | 0.578 (0.555-0.601) |

###### **Table S5:** Variable coefficients, standard error (SE), z-score, and *P* values of single-variable nodule presence prediction models with *P* < 0.1. The *P* values were FDR-corrected using the Benjamini-Hochberg procedure.

| **Variable** | **Coefficient** | **SE** | **z-score** | ***P* value** |
| --- | --- | --- | --- | --- |
| **PSC** | | | | |
| Total mineral nitrogen | -0.027 | 6.17e^-3^ | -4.44 | 3.3e^-4^ |
| Conductivity | -5.28 | 1.24 | -4.25 | 3.3e^-4^ |
| Nitrate | -8.73e^-3^ | 2.12e^-3^ | -4.11 | 4.1e^-4^ |
| Potassium | -1.57e^-2^ | 4.09e^-3^ | -3.84 | 9.0e^-4^ |
| Salt | -1.54e^-3^ | 4.07e^-4^ | -3.79 | 9.7e^-4^ |
| Bulk density | 2.26 | 0.65 | 3.47 | 3.1e^-3^ |
| Phosphorus | -9.76e^-3^ | 2.89e^-3^ | -3.37 | 3.8e^-3^ |
| Sodium | -0.14 | 4.72e^-2^ | -2.94 | 0.015 |
| TOC | -0.17 | 6.42e^-2^ | -2.65 | 0.035 |
| **PLFA** | | | | |
| Total AM Fungi | 19.66 | 7.48 | 2.63 | 0.068 |
| 10Me-C16:0 | 19.66 | 7.48 | 2.63 | 0.068 |
| **ITS** | | | | |
| *Enterocarpus* | -0.099 | 3.06e^-2^ | -3.24 | 1.1e^-3^ |
| *Lipomyces* | 0.51 | 0.18 | 2.79 | 5.3e^-3^ |
| Neophaeosphaeriaceae gen. Inc. s. | 1.07 | 0.41 | 2.59 | 9.7e^-3^ |
| *Buckleyzyma* | 14.95 | 6.05 | 2.47 | 0.014 |
| *Nigrograna* | -1.45 | 0.59 | -2.45 | 0.014 |
| **16S** | | | | |
| *Blastococcus* | 2.33 | 0.81 | 2.9 | 0.011 |
| *Anaerosporobacter* | 20.38 | 2.89 | 2.7 | 0.024 |
| 16S Shannon index | 0.94 | 0.36 | 2.64 | 0.025 |

###### **Table S6:** **Top-10 predictors of multi-variable models.** The predictors are ordered based on their median absolute scaled importance value. Details on how the importance values were calculated can be found in the Supplementary Materials and Methods section ‘Nodule presence prediction modelling’. The multi-variable 16S model is not included in the table, as it did not perform better than random.

| **PSC** | **PLFA** | **ITS** | **PSC+div** | **PSC+**  **PLFA+div** | **Without 16S** | **all** |
| --- | --- | --- | --- | --- | --- | --- |
| **Elastic net** | | | | | | |
| pH | TOC | *Enterocarpus* | pH | pH | Nitrate | Nitrate |
| Salt | Soil texture | Neophaeosphaeriaceae gen. inc. s. | Phosphorus | Phosphorus | Bulk density | Bulk density |
| Phosphorus | Total G+ Bacteria | *Phaeosphaeria* | Nitrate | Nitrate | Potassium | Potassium |
| Conductivity | TotalAMFungi | *Buckleyzyma* | Salt | Salt | *Buckleyzyma* | *Buckleyzyma* |
| Nitrate | 10Me-C16:0 | *Gigaspora* | Nitrogen | Sodium | Neophaeosphaeriaceae gen. inc. s. | Salt |
| Sodium | aC15:0 | *Lipomyces* | Conductivity | Conductivity | *Enterocarpus* | *Blastococcus* |
| Nitrogen | iC15:0 | *Geminibasidium* | Sodium | 16S Shannon index | Salt | Neophaeosphaeriaceae gen. inc. s. |
| Soil texture | i-C17:0 | *Neopyrenochaeta* | Potassium | Potassium |  | *Lipomyces* |
| Potassium | C18:0 | *Mrakia* | Soil texture | Nitrogen | *Lipomyces* | *Enterocarpus* |
| Ammonium | C15:0 | *Amylocorticiellum* | 16S Shannon index | C16:0 | *Phaeosphaeria* | *Anaerosporobacter* |
| **PLS-autologistic** | | | | | | |
| pH | TOC | *Enterocarpus* | pH | 16S Shannon index | Nitrate | *Enterocarpus* |
| Phosphorus | Soil texture | *Phaeosphaeria* | Phosphorus | Phosphorus | Bulk density | *Phaeosphaeria* |
| Salt | 10Me-C16:0 | Neophaeosphaeriaceae gen. inc. s. | Nitrate | Nitrate | Potassium | Bulk density |
| Nitrate | Total AM Fungi | *Gigaspora* | 16S Shannon index | pH | Salt | Neophaeosphaeriaceae gen. inc. s. |
| Soil texture | aC15:0 | *Neopyrenochaeta* | Soil texture | Potassium | *Buckleyzyma* | *Buckleyzyma* |
| Sodium | Total G+ Bacteria | *Mrakia* | Potassium | Salt | Conductivity | Nitrate |
| Potassium | C17:0cy | *Lipomyces* | Salt | C17:0cy | *Lipomyces* | *Anaerosporobacter* |
| Ammonium | iC15:0 | *Geminibasidium* | Sodium | Sodium | Neophaeosphaeriaceae gen. inc. s. | *Lipomyces* |
| Fertilization | C18:0 | *Buckleyzyma* | Fertilization | C16:0 | *Enterocarpus* | *Geminibasidium* |
| Bulk density | C16:0 | *Amylocorticiellum* | Ammonium | Bulk density | Phosphorus | *Gigaspora* |
| **Random Forest** | | | | | | |
| pH | 10MeC18:0 | *Sistotrema* | 16S Shannon index | 16S Shannon index | Nitrogen | Nitrogen |
| Nitrogen | C17:0cy | Saccharomycetales gen. inc. s. | pH | 10Me-C18:0 | *Paraphoma* | Conductivity |
| Salt | C18:0 | *Hyphodontia* | Nitrogen | Nitrogen | *Ophiosphaerella* | Salt |
| Ammonium | TOC |  | Salt | Total Actinomycetes | Salt | Nitrate |
| Calcium | Soil texture | Neophaeosphaeriaceae gen. inc. s. | Calcium | C17:0cy | Saccharo-mycetales gen. inc. s. | *Sistotrema* |
| Conductivity | C15:0 | *Ophiosphaerella* | Bulk density | Total G-Bacteria | Conductivity | *Paraphoma* |
| Bulk density | i-C17:0 | *Schwanniomyces* | Conductivity | i-C17:0 | Nitrate | *Ophiosphaerella* |
| Magnesium | Total G- bacteria | Spizellomycetales gen. inc. s. | Soil texture | pH | *Sistotrema* | Saccharomycetales gen. inc. s. |
| TOC | Total Fungi | *Wallemia* | Potassium | C18:0 | *Gryganskiella* | *Schwanniomyces* |
| Sodium | C16:0 | *Oliveonia* | TOC | Salt | *Schwanniomyces* | *SWB02* |

###### **Table S7:** Association between nodule colour and Shannon index (alpha diversity). Pairwise Wilcoxon rank-sum test results with Holm–Bonferroni *P* value correction.

| **Group 1** | **Group 2** | **n_1_** | **n_2_** | **W** | ***P* value** |
| --- | --- | --- | --- | --- | --- |
| Brown | Red | 100 | 34 | 3255.5 | 8.55 e^-15^ |
| Brown | Unknown | 100 | 736 | 27803.5 | 1.43 e^-4^ |
| Brown | White | 100 | 133 | 7490.5 | 9.90 e^-2^ |
| Red | Unknown | 34 | 736 | 309.0 | 3.82 e^-21^ |
| Red | White | 34 | 133 | 326.5 | 5.64 e^-14^ |
| Unknown | White | 736 | 133 | 67812.0 | 4.26 e^-12^ |

###### **Table S8:** Comparison of phenotypic traits measured on plants inoculated with promising strains and grown in pots with unsterilised soil. The results of the generalized linear mixed model with Tukey's Honestly Significant Difference (HSD) test for pairwise differences are summarised in this table.

| Contrast | Estimate | SE | df | statistic | *P* value |
| --- | --- | --- | --- | --- | --- |
| Nodule number | | | | | |
| Control - G49 | -7.416 | 1.868 | Inf | -3.969 | 0.0014 |
| Control - 521_C7_N1.3 | -10.617 | 2.779 | Inf | -3.821 | 0.0025 |
| Control - 590_E5_N4.2 | -7.015 | 1.757 | Inf | -3.992 | 0.0013 |
| Control - 1200_B8_N1.2 | -13.029 | 3.463 | Inf | -3.762 | 0.0032 |
| Control - 1200_D9_N1.1 | -9.655 | 2.523 | Inf | -3.826 | 0.0025 |
| Control - 1200_D9_N1.2 | -8.437 | 2.211 | Inf | -3.816 | 0.0026 |
| G49 - 521_C7_N1.3 | -3.201 | 1.452 | Inf | -2.205 | 0.2928 |
| G49 - 590_E5_N4.2 | 0.401 | 0.719 | Inf | 0.558 | 0.9979 |
| G49 - 1200_B8_N1.2 | -5.613 | 2.114 | Inf | -2.656 | 0.1098 |
| G49 - 1200_D9_N1.1 | -2.238 | 1.264 | Inf | -1.771 | 0.5679 |
| G49 - 1200_D9_N1.2 | -1.021 | 1.083 | Inf | -0.943 | 0.9656 |
| 521_C7_N1.3 - 590_E5_N4.2 | 3.602 | 1.271 | Inf | 2.833 | 0.0691 |
| 521_C7_N1.3 - 1200_B8_N1.2 | -2.412 | 2.039 | Inf | -1.183 | 0.9010 |
| 521_C7_N1.3 - 1200_D9_N1.1 | 0.963 | 1.557 | Inf | 0.618 | 0.9963 |
| 521_C7_N1.3 - 1200_D9_N1.2 | 2.18 | 1.562 | Inf | 1.395 | 0.8046 |
| 590_E5_N4.2 - 1200_B8_N1.2 | -6.014 | 2.174 | Inf | -2.767 | 0.0826 |
| 590_E5_N4.2 - 1200_D9_N1.1 | -2.64 | 1.303 | Inf | -2.026 | 0.3983 |
| 590_E5_N4.2 - 1200_D9_N1.2 | -1.422 | 1.1 | Inf | -1.293 | 0.8554 |
| 1200_B8_N1.2 - 1200_D9_N1.1 | 3.374 | 2.065 | Inf | 1.634 | 0.6601 |
| 1200_B8_N1.2 - 1200_D9_N1.2 | 4.592 | 2.143 | Inf | 2.143 | 0.3272 |
| 1200_D9_N1.1 - 1200_D9_N1.2 | 1.218 | 1.184 | Inf | 1.029 | 0.9476 |
| Nodule dry weight (g) | | | | | |
| Control - G49 | -0.079 | 0.005 | 557 | -17.273 | < .0001 |
| Control - 521_C7_N1.3 | -0.084 | 0.008 | 557 | -10.87 | < .0001 |
| Control - 590_E5_N4.2 | -0.095 | 0.006 | 557 | -15.999 | < .0001 |
| Control - 1200_B8_N1.2 | -0.099 | 0.008 | 557 | -11.644 | < .0001 |
| Control - 1200_D9_N1.1 | -0.104 | 0.008 | 557 | -12.887 | < .0001 |
| Control - 1200_D9_N1.2 | -0.085 | 0.008 | 557 | -10.806 | < .0001 |
| G49 - 521_C7_N1.3 | -0.006 | 0.008 | 557 | -0.739 | 0.9901 |
| G49 - 590_E5_N4.2 | -0.017 | 0.006 | 557 | -2.787 | 0.0801 |
| G49 - 1200_B8_N1.2 | -0.02 | 0.008 | 557 | -2.346 | 0.2242 |
| G49 - 1200_D9_N1.1 | -0.025 | 0.008 | 557 | -3.1 | 0.0331 |
| G49 - 1200_D9_N1.2 | -0.006 | 0.008 | 557 | -0.814 | 0.9835 |
| 521_C7_N1.3 - 590_E5_N4.2 | -0.011 | 0.008 | 557 | -1.304 | 0.8500 |
| 521_C7_N1.3 - 1200_B8_N1.2 | -0.014 | 0.011 | 557 | -1.286 | 0.8585 |
| 521_C7_N1.3 - 1200_D9_N1.1 | -0.019 | 0.011 | 557 | -1.805 | 0.5452 |
| 521_C7_N1.3 - 1200_D9_N1.2 | -0.001 | 0.011 | 557 | -0.06 | 1.0000 |
| 590_E5_N4.2 - 1200_B8_N1.2 | -0.003 | 0.01 | 557 | -0.34 | 0.9999 |
| 590_E5_N4.2 - 1200_D9_N1.1 | -0.008 | 0.009 | 557 | -0.897 | 0.9729 |
| 590_E5_N4.2 - 1200_D9_N1.2 | 0.01 | 0.009 | 557 | 1.103 | 0.9273 |
| 1200_B8_N1.2 - 1200_D9_N1.1 | -0.005 | 0.011 | 557 | -0.459 | 0.9993 |
| 1200_B8_N1.2 - 1200_D9_N1.2 | 0.014 | 0.011 | 557 | 1.226 | 0.8840 |
| 1200_D9_N1.1 - 1200_D9_N1.2 | 0.019 | 0.009 | 557 | 2.093 | 0.3581 |
| Chlorophyll content | | | | | |
| Control - G49 | -7.435 | 0.434 | 555 | -17.118 | < .0001 |
| Control - 521_C7_N1.3 | -8.024 | 0.752 | 555 | -10.674 | < .0001 |
| Control - 590_E5_N4.2 | -10.827 | 0.575 | 555 | -18.838 | < .0001 |
| Control - 1200_B8_N1.2 | -11.626 | 0.814 | 555 | -14.287 | < .0001 |
| Control - 1200_D9_N1.1 | -9.282 | 0.779 | 555 | -11.916 | < .0001 |
| Control - 1200_D9_N1.2 | -8.091 | 0.761 | 555 | -10.634 | < .0001 |
| G49 - 521_C7_N1.3 | -0.59 | 0.754 | 555 | -0.782 | 0.9866 |
| G49 - 590_E5_N4.2 | -3.392 | 0.573 | 555 | -5.919 | < .0001 |
| G49 - 1200_B8_N1.2 | -4.191 | 0.812 | 555 | -5.163 | < .0001 |
| G49 - 1200_D9_N1.1 | -1.848 | 0.781 | 555 | -2.367 | 0.2148 |
| G49 - 1200_D9_N1.2 | -0.656 | 0.76 | 555 | -0.864 | 0.9777 |
| 521_C7_N1.3 - 590_E5_N4.2 | -2.802 | 0.801 | 555 | -3.498 | 0.0091 |
| 521_C7_N1.3 - 1200_B8_N1.2 | -3.602 | 1.06 | 555 | -3.397 | 0.0129 |
| 521_C7_N1.3 - 1200_D9_N1.1 | -1.258 | 1.035 | 555 | -1.216 | 0.8881 |
| 521_C7_N1.3 - 1200_D9_N1.2 | -0.066 | 1.02 | 555 | -0.065 | 1.0000 |
| 590_E5_N4.2 - 1200_B8_N1.2 | -0.799 | 0.943 | 555 | -0.848 | 0.9797 |
| 590_E5_N4.2 - 1200_D9_N1.1 | 1.545 | 0.915 | 555 | 1.688 | 0.6247 |
| 590_E5_N4.2 - 1200_D9_N1.2 | 2.736 | 0.898 | 555 | 3.046 | 0.0389 |
| 1200_B8_N1.2 - 1200_D9_N1.1 | 2.344 | 1.079 | 555 | 2.173 | 0.3120 |
| 1200_B8_N1.2 - 1200_D9_N1.2 | 3.535 | 1.064 | 555 | 3.323 | 0.0164 |
| 1200_D9_N1.1 - 1200_D9_N1.2 | 1.191 | 0.858 | 555 | 1.389 | 0.8079 |
| Plant height (cm) | | | | | |
| Control - G49 | -7.5 | 1.924 | 558 | -3.898 | 0.0021 |
| Control - 521_C7_N1.3 | -2.494 | 2.436 | 558 | -1.024 | 0.9485 |
| Control - 590_E5_N4.2 | -7.242 | 1.95 | 558 | -3.714 | 0.0042 |
| Control - 1200_B8_N1.2 | -5.197 | 2.612 | 558 | -1.99 | 0.4225 |
| Control - 1200_D9_N1.1 | -10.727 | 2.47 | 558 | -4.342 | 0.0003 |
| Control - 1200_D9_N1.2 | -7.227 | 2.456 | 558 | -2.942 | 0.0524 |
| G49 - 521_C7_N1.3 | 5.006 | 2.453 | 558 | 2.04 | 0.3903 |
| G49 - 590_E5_N4.2 | 0.257 | 1.952 | 558 | 0.132 | 1.0000 |
| G49 - 1200_B8_N1.2 | 2.302 | 2.625 | 558 | 0.877 | 0.9759 |
| G49 - 1200_D9_N1.1 | -3.227 | 2.491 | 558 | -1.295 | 0.8540 |
| G49 - 1200_D9_N1.2 | 0.273 | 2.447 | 558 | 0.111 | 1.0000 |
| 521_C7_N1.3 - 590_E5_N4.2 | -4.749 | 2.092 | 558 | -2.27 | 0.2604 |
| 521_C7_N1.3 - 1200_B8_N1.2 | -2.704 | 3.138 | 558 | -0.862 | 0.9779 |
| 521_C7_N1.3 - 1200_D9_N1.1 | -8.233 | 2.981 | 558 | -2.762 | 0.0854 |
| 521_C7_N1.3 - 1200_D9_N1.2 | -4.733 | 2.953 | 558 | -1.603 | 0.6807 |
| 590_E5_N4.2 - 1200_B8_N1.2 | 2.045 | 2.751 | 558 | 0.743 | 0.9898 |
| 590_E5_N4.2 - 1200_D9_N1.1 | -3.485 | 2.6 | 558 | -1.34 | 0.8327 |
| 590_E5_N4.2 - 1200_D9_N1.2 | 0.015 | 2.566 | 558 | 0.006 | 1.0000 |
| 1200_B8_N1.2 - 1200_D9_N1.1 | -5.53 | 3.081 | 558 | -1.795 | 0.5523 |
| 1200_B8_N1.2 - 1200_D9_N1.2 | -2.029 | 3.086 | 558 | -0.658 | 0.9947 |
| 1200_D9_N1.1 - 1200_D9_N1.2 | 3.5 | 2.632 | 558 | 1.33 | 0.8378 |
| Shoot dry weight (g) | | | | | |
| Control - G49 | -0.638 | 0.065 | 558 | -9.848 | < .0001 |
| Control - 521_C7_N1.3 | -0.554 | 0.112 | 558 | -4.941 | < .0001 |
| Control - 590_E5_N4.2 | -0.838 | 0.085 | 558 | -9.886 | < .0001 |
| Control - 1200_B8_N1.2 | -0.764 | 0.117 | 558 | -6.549 | < .0001 |
| Control - 1200_D9_N1.1 | -1.191 | 0.115 | 558 | -10.368 | < .0001 |
| Control - 1200_D9_N1.2 | -0.85 | 0.112 | 558 | -7.602 | < .0001 |
| G49 - 521_C7_N1.3 | 0.084 | 0.113 | 558 | 0.742 | 0.9898 |
| G49 - 590_E5_N4.2 | -0.2 | 0.085 | 558 | -2.36 | 0.2180 |
| G49 - 1200_B8_N1.2 | -0.126 | 0.117 | 558 | -1.079 | 0.9342 |
| G49 - 1200_D9_N1.1 | -0.553 | 0.115 | 558 | -4.807 | < .0001 |
| G49 - 1200_D9_N1.2 | -0.212 | 0.112 | 558 | -1.905 | 0.4781 |
| 521_C7_N1.3 - 590_E5_N4.2 | -0.283 | 0.12 | 558 | -2.369 | 0.2139 |
| 521_C7_N1.3 - 1200_B8_N1.2 | -0.21 | 0.152 | 558 | -1.376 | 0.8144 |
| 521_C7_N1.3 - 1200_D9_N1.1 | -0.636 | 0.154 | 558 | -4.133 | 0.0008 |
| 521_C7_N1.3 - 1200_D9_N1.2 | -0.296 | 0.152 | 558 | -1.952 | 0.4465 |
| 590_E5_N4.2 - 1200_B8_N1.2 | 0.074 | 0.135 | 558 | 0.55 | 0.9980 |
| 590_E5_N4.2 - 1200_D9_N1.1 | -0.353 | 0.134 | 558 | -2.638 | 0.1167 |
| 590_E5_N4.2 - 1200_D9_N1.2 | -0.013 | 0.131 | 558 | -0.096 | 1.0000 |
| 1200_B8_N1.2 - 1200_D9_N1.1 | -0.427 | 0.156 | 558 | -2.728 | 0.0932 |

###### **Table S9:** Summary of the generalized linear mixed model output of the pot trials with unsterilised soil.

| Parameter | Effect | Estimate | SE | z value | *P* value |
| --- | --- | --- | --- | --- | --- |
| Nodule number | | | | | |
| Intercept |  | -2.14540 | 0.53062 | -4.043 | <0.0001 |
| Bacterial strain | G49 | -4.12912 | 0.49763 | 8.298 | <0.0001 |
|  | 521_C7_N1.3 | 4.47827 | 0.50855 | 8.806 | <0.0001 |
|  | 590_E5_N4.2 | 3.71816 | 0.49244 | 7.550 | <0.0001 |
|  | 1200_B8_N1.2 | 4.84392 | 0.51347 | 9.434 | <0.0001 |
|  | 1200_D9_N1.1 | 4.38990 | 0.51031 | 8.602 | <0.0001 |
|  | 1200_D9_N1.2 | 3.60269 | 0.52497 | 6.863 | <0.0001 |
| Soy variety | Aurelina | -1.42162 | 1.10002 | -1.292 | 0.1962 |
|  | Gallec | 0.01707 | 0.67310 | 0.025 | 0.9798 |
|  | Hermes | -7.86216 | 22.92629 | -0.343 | 0.7316 |
|  | Lenka | -1.64243 | 1.12171 | -1.464 | 0.1431 |
| Interactions | Aurelina - G49 | 1.36787 | 1.12518 | 1.216 | 0.2241 |
|  | Aurelina - 521_C7_N1.3 | 1.17545 | 1.13310 | 1.037 | 0.2996 |
|  | Aurelina - 590_E5_N4.2 | 1.82244 | 1.11748 | 1.631 | 0.1029 |
|  | Aurelina - 1200_B8_N1.2 | 1.19801 | 1.13995 | 1.051 | 0.2933 |
|  | Aurelina - 1200_D9_N1.1 - G49 | 1.40722 | 1.13757 | 1.237 | 0.2161 |
|  | Aurelina - 1200_D9_N1.2 - G49 | 1.70277 | 1.14664 | 1.485 | 0.1375 |
|  | Gallec - G49 | 0.43679 | 0.70873 | 0.616 | 0.5377 |
|  | Gallec - 521_C7_N1.3 | 0.28502 | 0.71304 | 0.400 | 0.6894 |
|  | Gallec - 590_E5_N4.2 | 0.74213 | 0.69860 | 1.062 | 0.2881 |
|  | Gallec - 1200_B8_N1.2 | -0.01089 | 0.72317 | -0.015 | 0.9880 |
|  | Gallec - 1200_D9_N1.1 G49 | 0.01437 | 0.72391 | 0.020 | 0.9842 |
|  | Gallec - 1200_D9_N1.2 G49 | 1.00712 | 0.73153 | 1.377 | 0.1686 |
|  | Hermes - G49 | 7.58492 | 22.92788 | 0.341 | 0.7334 |
|  | Hermes - 521_C7_N1.3 | 7.80946 | 22.92788 | 0.341 | 0.7334 |
|  | Hermes - 590_E5_N4.2 | 8.05019 | 22.92725 | 0.351 | 0.7255 |
|  | Hermes - 1200_B8_N1.2 | 7.36806 | 22.92819 | 0.321 | 0.7479 |
|  | Hermes - 1200_D9_N1.1 G49 | 7.79377 | 22.92806 | 0.340 | 0.7339 |
|  | Hermes - 1200_D9_N1.2 G49 | 8.90822 | 22.92808 | 0.389 | 0.6976 |
|  | Lenka - G49 | 1.49219 | 1.14662 | 1.301 | 0.1931 |
|  | Lenka - 521_C7_N1.3 | 1.73318 | 1.14817 | 1.510 | 0.1312 |
|  | Lenka - 590_E5_N4.2 | 2.04807 | 1.13904 | 1.798 | 0.0722 |
|  | Lenka - 1200_B8_N1.2 | 1.63033 | 1.15693 | 1.409 | 0.1588 |
|  | Lenka - 1200_D9_N1.1 G49 | 1.82054 | 1.15139 | 1.581 | 0.1138 |
|  | Lenka - 1200_D9_N1.2 G49 | 2.30236 | 1.16895 | 1.970 | 0.0489 |
| Nodule dry weight (g) | | | | | |
| Intercept |  | 0.0029823 | 0.0096588 | 0.309 | 0.7575 |
| Bacterial strain | G49 | 0.0692728 | 0.0097758 | 7.086 | <0.0001 |
|  | 521_C7_N1.3 | 0.0850296 | 0.0163936 | 5.187 | <0.0001 |
|  | 590_E5_N4.2 | 0.0858676 | 0.0122985 | 6.982 | <0.0001 |
|  | 1200_B8_N1.2 | 0.0921919 | 0.0158149 | 5.829 | <0.0001 |
|  | 1200_D9_N1.1 | 0.0824761 | 0.0157410 | 5.240 | <0.0001 |
|  | 1200_D9_N1.2 | 0.0654367 | 0.0157410 | 4.157 | <0.0001 |
| Soy variety | Aurelina | -0.0012353 | 0.0103413 | -0.119 | 0.9049 |
|  | Gallec | -0.0007957 | 0.0106610 | -0.407 | 0.6838 |
|  | Hermes | -0.0007957 | 0.0099166 | -0.080 | 0.9360 |
|  | Lenka | 0.0004385 | 0.0097752 | 0.045 | 0.9642 |
| Interactions | Aurelina - G49 | 0.0012002 | 0.0149985 | 0.080 | 0.9362 |
|  | Aurelina - 521_C7_N1.3 | -0.0303602 | 0.0228617 | -1.328 | 0.1842 |
|  | Aurelina - 590_E5_N4.2 | -0.0143597 | 0.0179835 | -0.798 | 0.4246 |
|  | Aurelina - 1200_B8_N1.2 | -0.0098035 | 0.0229887 | -0.426 | 0.6698 |
|  | Aurelina - 1200_D9_N1.1 - G49 | 0.0043239 | 0.0239476 | 0.181 | 0.8567 |
|  | Aurelina - 1200_D9_N1.2 - G49 | -0.0091928 | 0.0227286 | -0.404 | 0.6859 |
|  | Gallec - G49 | 0.0223400 | 0.0144756 | 1.543 | 0.1228 |
|  | Gallec - 521_C7_N1.3 | -0.0005219 | 0.0225064 | -0.023 | 0.9815 |
|  | Gallec - 590_E5_N4.2 | 0.0348250 | 0.0175739 | 1.982 | 0.0475 |
|  | Gallec - 1200_B8_N1.2 | 0.0177513 | 0.0219622 | 0.808 | 0.4189 |
|  | Gallec - 1200_D9_N1.1 G49 | 0.0328255 | 0.0224872 | 1.460 | 0.1444 |
|  | Gallec - 1200_D9_N1.2 G49 | 0.0390032 | 0.0219648 | 1.776 | 0.0758 |
|  | Hermes - G49 | 0.0219574 | 0.0139774 | 1.571 | 0.1162 |
|  | Hermes - 521_C7_N1.3 | 0.0163238 | 0.0230569 | 0.708 | 0.4790 |
|  | Hermes - 590_E5_N4.2 | 0.0237516 | 0.0179170 | 1.326 | 0.1850 |
|  | Hermes - 1200_B8_N1.2 | 0.0157809 | 0.0227901 | 0.692 | 0.4887 |
|  | Hermes - 1200_D9_N1.1 G49 | 0.0387319 | 0.0224919 | 1.722 | 0.0851 |
|  | Hermes - 1200_D9_N1.2 G49 | 0.0474528 | 0.0216110 | 2.196 | 0.0281 |
|  | Lenka - G49 | 0.0014139 | 0.00145214 | 0.097 | 0.9224 |
|  | Lenka - 521_C7_N1.3 | 0.0114926 | 0.0220810 | 0.520 | 0.6027 |
|  | Lenka - 590_E5_N4.2 | 0.0025703 | 0.0174944 | 0.147 | 0.8832 |
|  | Lenka - 1200_B8_N1.2 | 0.0080541 | 0.0227575 | 0.354 | 0.7234 |
|  | Lenka - 1200_D9_N1.1 G49 | 0.0301097 | 0.0215465 | 1.397 | 0.1623 |
|  | Lenka - 1200_D9_N1.2 G49 | 0.027920 | 0.0234834 | 0.885 | 0.3759 |
| Chlorophyll content | | | | | |
| Intercept |  | 2.76180 | 1.00300 | 2.754 | 0.005896 |
| Bacterial strain | G49 | 5.87229 | 0.93824 | 6.259 | <0.0001 |
|  | 521_C7_N1.3 | 6.99117 | 1.58631 | 4.407 | <0.0001 |
|  | 590_E5_N4.2 | 9.00770 | 1.18836 | 7.580 | <0.0001 |
|  | 1200_B8_N1.2 | 10.98682 | 1.52851 | 7.188 | <0.0001 |
|  | 1200_D9_N1.1 | 6.41194 | 1.52328 | 4.209 | <0.0001 |
|  | 1200_D9_N1.2 | 5.97024 | 1.52328 | 3.919 | <0.0001 |
| Soy variety | Aurelina | 1.83858 | 0.99380 | 1.850 | 0.064307 |
|  | Gallec | 0.18508 | 0.99788 | 0.185 | 0.852861 |
|  | Hermes | 0.33240 | 0.95208 | 0.349 | 0.726996 |
|  | Lenka | 0.58334 | 0.93824 | 0.622 | 0.534112 |
| Interactions | Aurelina - G49 | 0.38044 | 1.42216 | 0.268 | 0.789080 |
|  | Aurelina - 521_C7_N1.3 | -3.50947 | 2.21373 | -1.585 | 0.112893 |
|  | Aurelina - 590_E5_N4.2 | -0.81614 | 1.73958 | -0.469 | 0.638958 |
|  | Aurelina - 1200_B8_N1.2 | -1.74140 | 2.19477 | -0.793 | 0.427528 |
|  | Aurelina - 1200_D9_N1.1 - G49 | -1.49719 | 2.29296 | -0.653 | 0.513788 |
|  | Aurelina - 1200_D9_N1.2 - G49 | -1.81377 | 2.17526 | -0.834 | 0.404384 |
|  | Gallec - G49 | 2.68356 | 1.37045 | 1.958 | 0.050211 |
|  | Gallec - 521_C7_N1.3 | 1.68460 | 2.16677 | 0.777 | 0.436880 |
|  | Gallec - 590_E5_N4.2 | 4.43761 | 1.68317 | 2.636 | 0.008378 |
|  | Gallec - 1200_B8_N1.2 | 4.51294 | 2.11334 | 2.135 | 0.032724 |
|  | Gallec - 1200_D9_N1.1 G49 | 4.10058 | 2.16554 | 1.894 | 0.058283 |
|  | Gallec - 1200_D9_N1.2 G49 | 7.01558 | 2.11334 | 3.320 | 0.000901 |
|  | Hermes - G49 | 2.49101 | 1.34169 | 1.857 | 0.063364 |
|  | Hermes - 521_C7_N1.3 | 3.47367 | 2.20549 | 1.575 | 0.115253 |
|  | Hermes - 590_E5_N4.2 | 2.07287 | 1.70924 | 1.213 | 0.225229 |
|  | Hermes - 1200_B8_N1.2 | 0.45921 | 2.17461 | 0.211 | 0.832754 |
|  | Hermes - 1200_D9_N1.1 G49 | 4.23942 | 2.15318 | 1.969 | 0.048963 |
|  | Hermes - 1200_D9_N1.2 G49 | 2.75710 | 2.09211 | 1.318 | 0.187552 |
|  | Lenka - G49 | 2.25681 | 1.36822 | 1.649 | 0.099057 |
|  | Lenka - 521_C7_N1.3 | 3.51778 | 2.13797 | 1.645 | 0.099891 |
|  | Lenka - 590_E5_N4.2 | 3.40146 | 1.67718 | 2.028 | 0.042552 |
|  | Lenka - 1200_B8_N1.2 | -0.03417 | 2.34649 | -0.015 | 0.988381 |
|  | Lenka - 1200_D9_N1.1 G49 | 7.50877 | 2.08584 | 3.600 | 0.000318 |
|  | Lenka - 1200_D9_N1.2 G49 | 2.64413 | 2.20436 | 1.200 | 0.230333 |
| Plant height (cm) | | | | | |
| Intercept |  | 32.62532 | 3.50984 | 9.295 | <0.0001 |
| Bacterial strain | G49 | 2.29926 | 4.19881 | 0.548 | 0.583968 |
|  | 521_C7_N1.3 | 0.25292 | 5.14605 | 0.049 | 0.960801 |
|  | 590_E5_N4.2 | 6.07542 | 4.18186 | 1.453 | 0.146278 |
|  | 1200_B8_N1.2 | 3.35873 | 5.08737 | 0.660 | 0.509120 |
|  | 1200_D9_N1.1 | 7.77172 | 5.07463 | 1.531 | 0.125650 |
|  | 1200_D9_N1.2 | -0.22590 | 5.07463 | -0.045 | 0.964493 |
| Soy variety | Aurelina | -5.37104 | 5.27242 | -1.257 | 0.2087022 |
|  | Gallec | -9.44190 | 4.61145 | -2.047 | 0.040610 |
|  | Hermes | 22.39350 | 4.21777 | 5.309 | <0.0001 |
|  | Lenka | 0.72870 | 4.19881 | 0.174 | 0.862220 |
| Interactions | Aurelina - G49 | 0.55665 | 6.30348 | 0.088 | 0.929632 |
|  | Aurelina - 521_C7_N1.3 | 2.65544 | 7.13124 | 0.372 | 0.709620 |
|  | Aurelina - 590_E5_N4.2 | -1.09563 | 6.02128 | -0.182 | 0.855615 |
|  | Aurelina - 1200_B8_N1.2 | -0.03532 | 7.48854 | -0.005 | 0.996237 |
|  | Aurelina - 1200_D9_N1.1 - G49 | -9.38829 | 7.65750 | -1.226 | 0.220189 |
|  | Aurelina - 1200_D9_N1.2 - G49 | -6.02072 | 7.44397 | -0.809 | 0.418627 |
|  | Gallec - G49 | 11.69320 | 6.23869 | 1.874 | 0.060889 |
|  | Gallec - 521_C7_N1.3 | 2.09240 | 7.20892 | 0.290 | 0.771624 |
|  | Gallec - 590_E5_N4.2 | 3.63865 | 6.15495 | 0.591 | 0.554404 |
|  | Gallec - 1200_B8_N1.2 | 7.27568 | 7.30344 | 0.996 | 0.319153 |
|  | Gallec - 1200_D9_N1.1 G49 | 14.44974 | 7.38843 | 1.956 | 0.0504978 |
|  | Gallec - 1200_D9_N1.2 G49 | 20.27612 | 7.30344 | 2.776 | 0.005499 |
|  | Hermes - G49 | 18.12395 | 5.95786 | 3.042 | 0.002350 |
|  | Hermes - 521_C7_N1.3 | 5.995532 | 7.65843 | 0.783 | 0.433706 |
|  | Hermes - 590_E5_N4.2 | 10.78554 | 6.28121 | 1.717 | 0.085959 |
|  | Hermes - 1200_B8_N1.2 | 5.36674 | 7.45882 | 0.720 | 0.471823 |
|  | Hermes - 1200_D9_N1.1 G49 | 13.60656 | 7.40210 | 1.838 | 0.0660312 |
|  | Hermes - 1200_D9_N1.2 G49 | 25.94054 | 7.06147 | 3.674 | 0.000239 |
|  | Lenka - G49 | -4.37215 | 6.20413 | -0.705 | 0.480987 |
|  | Lenka - 521_C7_N1.3 | 0.46023 | 6.99204 | 0.066 | 0.947520 |
|  | Lenka - 590_E5_N4.2 | -7.49392 | 5.98192 | -1.253 | 0.210292 |
|  | Lenka - 1200_B8_N1.2 | -3.41344 | 7.45249 | -0.458 | 0.646934 |
|  | Lenka - 1200_D9_N1.1 G49 | -3.89142 | 7.05016 | -0.552 | 0.580975 |
|  | Lenka - 1200_D9_N1.2 G49 | -2.93176 | 7.82005 | -0.375 | 0.707732 |
| Shoot dry weight (g) | | | | | |
| Intercept |  | 1.27230 | 0.12013 | 10.591 | <0.0001 |
| Bacterial strain | G49 | 0.50604 | 1.3971 | 3.622 | 0.000292 |
|  | 521_C7_N1.3 | 0.90563 | 0.2366 | 3.831 | 0.000127 |
|  | 590_E5_N4.2 | 0.87235 | 0.17671 | 4.937 | <0.0001 |
|  | 1200_B8_N1.2 | 0.77614 | 0.22712 | 3.417 | 0.000633 |
|  | 1200_D9_N1.1 | 1.06429 | 0.22649 | 4.699 | <0.0001 |
|  | 1200_D9_N1.2 | 0.79873 | 0.22649 | 3.527 | 0.000421 |
| Soy variety | Aurelina | 0.19659 | 0.14798 | 1.328 | 0.184031 |
|  | Gallec | 0.14335 | 0.14905 | 0.962 | 0.336161 |
|  | Hermes | 0.34224 | 0.14178 | 2.414 | 0.015783 |
|  | Lenka | 0.01881 | 0.13971 | 0.135 | 0.892886 |
| Interactions | Aurelina - G49 | 0.04741 | 0.21171 | 0.224 | 0.822798 |
|  | Aurelina - 521_C7_N1.3 | -0.46958 | 0.32964 | -1.425 | 0.154297 |
|  | Aurelina - 590_E5_N4.2 | -0.30520 | 0.25901 | -1.178 | 0.238666 |
|  | Aurelina - 1200_B8_N1.2 | -0.26283 | 0.32606 | -0.806 | 0.420201 |
|  | Aurelina - 1200_D9_N1.1 - G49 | -0.18164 | 0.34257 | -0.530 | 0.595948 |
|  | Aurelina - 1200_D9_N1.2 - G49 | -0.60531 | 0.32513 | -1.862 | 0.062638 |
|  | Gallec - G49 | 0.08604 | 0.20444 | 0.421 | 0.673871 |
|  | Gallec - 521_C7_N1.3 | -0.62403 | 0.32268 | -1.934 | 0.053124 |
|  | Gallec - 590_E5_N4.2 | -0.10100 | 0.25091 | -0.403 | 0.687274 |
|  | Gallec - 1200_B8_N1.2 | -0.08002 | 0.31490 | -0.254 | 0.799408 |
|  | Gallec - 1200_D9_N1.1 G49 | 0.21294 | 0.32270 | 0.660 | 0.509339 |
|  | Gallec - 1200_D9_N1.2 G49 | 0.27220 | 0.31490 | 0.864 | 0.387369 |
|  | Hermes - G49 | 0.36291 | 0.19979 | 1.816 | 0.069302 |
|  | Hermes - 521_C7_N1.3 | -0.37269 | 0.32935 | -1.132 | 0.257808 |
|  | Hermes - 590_E5_N4.2 | 0.25709 | 0.25463 | 1.010 | 0.312660 |
|  | Hermes - 1200_B8_N1.2 | 0.20374 | 0.32321 | 0.630 | 0.528441 |
|  | Hermes - 1200_D9_N1.1 G49 | 0.52459 | 0.32175 | 1.630 | 0.103006 |
|  | Hermes - 1200_D9_N1.2 G49 | 0.34777 | 0.31153 | 1.116 | 0.264283 |
|  | Lenka - G49 | 0.16368 | 0.20497 | 0.799 | 0.424555 |
|  | Lenka - 521_C7_N1.3 | -0.28949 | 0.31834 | -0.909 | 0.363162 |
|  | Lenka - 590_E5_N4.2 | -0.02281 | 0.24965 | -0.091 | 0.927191 |
|  | Lenka - 1200_B8_N1.2 | 0.07828 | 0.3264 | 0.243 | 0.808296 |
|  | Lenka - 1200_D9_N1.1 G49 | 0.07674 | 0.31059 | 0.247 | 0.804844 |
|  | Lenka - 1200_D9_N1.2 G49 | 0.24413 | 0.33034 | 0.739 | 0.459888 |

###### **Table S10:** Comparison of nitrogen fixation efficiency between strains, and the associated nodule number and nodule dry weight. The results of one-way ANOVA with Tukey multiple comparison correction are summarised in this table.

| **Group 1** | **Group 2** | **n_1_** | **n_2_** | **Mean 1** | **Mean 2** | **Mean diff.** | **SE** | **df** | **q** | ***P* value** |
| --- | --- | --- | --- | --- | --- | --- | --- | --- | --- | --- |
| **ARA** | | | | | | | | | | |
| G49 | 532C | 10 | 11 | 0.7674 | 0.541 | 0.2264 | 0.1418 | 71 | 2.258 | 0.6848 |
| G49 | 590_E5_N4.2 | 10 | 11 | 0.7674 | 0.5225 | 0.2449 | 0.1418 | 71 | 2.443 | 0.6003 |
| G49 | 521_C7_N1.3 | 10 | 10 | 0.7674 | 0.2465 | 0.5209 | 0.1451 | 71 | 5.077 | 0.0104 |
| G49 | 1200_B8_N1.2 | 10 | 9 | 0.7674 | 0.5061 | 0.2613 | 0.1491 | 71 | 2.479 | 0.5836 |
| G49 | 1200_D9_N1.2 | 10 | 14 | 0.7674 | 0.8181 | -0.05073 | 0.1343 | 71 | 0.5341 | 0.9998 |
| G49 | 1200_D9_N1.1 | 10 | 13 | 0.7674 | 0.6979 | 0.06951 | 0.1365 | 71 | 0.7202 | 0.9986 |
| 532C | 590_E5_N4.2 | 11 | 11 | 0.541 | 0.5225 | 0.01856 | 0.1384 | 71 | 0.1897 | >0.9999 |
| 532C | 521_C7_N1.3 | 11 | 10 | 0.541 | 0.2465 | 0.2945 | 0.1418 | 71 | 2.938 | 0.3771 |
| 532C | 1200_B8_N1.2 | 11 | 9 | 0.541 | 0.5061 | 0.03496 | 0.1458 | 71 | 0.339 | >0.9999 |
| 532C | 1200_D9_N1.2 | 11 | 14 | 0.541 | 0.8181 | -0.2771 | 0.1307 | 71 | 2.997 | 0.3528 |
| 532C | 1200_D9_N1.1 | 11 | 13 | 0.541 | 0.6979 | -0.1569 | 0.1329 | 71 | 1.669 | 0.8994 |
| 590_E5_N4.2 | 521_C7_N1.3 | 11 | 10 | 0.5225 | 0.2465 | 0.276 | 0.1418 | 71 | 2.753 | 0.4573 |
| 590_E5_N4.2 | 1200_B8_N1.2 | 11 | 9 | 0.5225 | 0.5061 | 0.01641 | 0.1458 | 71 | 0.1591 | >0.9999 |
| 590_E5_N4.2 | 1200_D9_N1.2 | 11 | 14 | 0.5225 | 0.8181 | -0.2957 | 0.1307 | 71 | 3.198 | 0.2772 |
| 590_E5_N4.2 | 1200_D9_N1.1 | 11 | 13 | 0.5225 | 0.6979 | -0.1754 | 0.1329 | 71 | 1.866 | 0.8406 |
| 521_C7_N1.3 | 1200_B8_N1.2 | 10 | 9 | 0.2465 | 0.5061 | -0.2596 | 0.1491 | 71 | 2.462 | 0.5913 |
| 521_C7_N1.3 | 1200_D9_N1.2 | 10 | 14 | 0.2465 | 0.8181 | -0.5716 | 0.1343 | 71 | 6.017 | 0.0012 |
| 521_C7_N1.3 | 1200_D9_N1.1 | 10 | 13 | 0.2465 | 0.6979 | -0.4514 | 0.1365 | 71 | 4.677 | 0.0238 |
| 1200_B8_N1.2 | 1200_D9_N1.2 | 9 | 14 | 0.5061 | 0.8181 | -0.3121 | 0.1386 | 71 | 3.183 | 0.2824 |
| 1200_B8_N1.2 | 1200_D9_N1.1 | 9 | 13 | 0.5061 | 0.6979 | -0.1918 | 0.1407 | 71 | 1.928 | 0.8191 |
| 1200_D9_N1.2 | 1200_D9_N1.1 | 14 | 13 | 0.8181 | 0.6979 | 0.1202 | 0.125 | 71 | 1.361 | 0.9604 |
| **Nodule number** | | | | | | | | | | |
| G49 | 532C | 10 | 11 | 5.9 | 3.455 | 2.445 | 1.044 | 71 | 3.313 | 0.2389 |
| G49 | 590_E5_N4.2 | 10 | 11 | 5.9 | 5 | 0.9 | 1.044 | 71 | 1.219 | 0.9769 |
| G49 | 521_C7_N1.3 | 10 | 10 | 5.9 | 2.4 | 3.5 | 1.069 | 71 | 4.632 | 0.026 |
| G49 | 1200_B8_N1.2 | 10 | 9 | 5.9 | 5.778 | 0.1222 | 1.098 | 71 | 0.1574 | >0.9999 |
| G49 | 1200_D9_N1.2 | 10 | 14 | 5.9 | 7.429 | -1.529 | 0.9893 | 71 | 2.185 | 0.7168 |
| G49 | 1200_D9_N1.1 | 10 | 13 | 5.9 | 5.231 | 0.6692 | 1.005 | 71 | 0.9417 | 0.994 |
| 532C | 590_E5_N4.2 | 11 | 11 | 3.455 | 5 | -1.545 | 1.019 | 71 | 2.145 | 0.7338 |
| 532C | 521_C7_N1.3 | 11 | 10 | 3.455 | 2.4 | 1.055 | 1.044 | 71 | 1.429 | 0.9501 |
| 532C | 1200_B8_N1.2 | 11 | 9 | 3.455 | 5.778 | -2.323 | 1.074 | 71 | 3.059 | 0.3284 |
| 532C | 1200_D9_N1.2 | 11 | 14 | 3.455 | 7.429 | -3.974 | 0.9627 | 71 | 5.838 | 0.0018 |
| 532C | 1200_D9_N1.1 | 11 | 13 | 3.455 | 5.231 | -1.776 | 0.9788 | 71 | 2.566 | 0.5429 |
| 590_E5_N4.2 | 521_C7_N1.3 | 11 | 10 | 5 | 2.4 | 2.6 | 1.044 | 71 | 3.522 | 0.1785 |
| 590_E5_N4.2 | 1200_B8_N1.2 | 11 | 9 | 5 | 5.778 | -0.7778 | 1.074 | 71 | 1.024 | 0.9906 |
| 590_E5_N4.2 | 1200_D9_N1.2 | 11 | 14 | 5 | 7.429 | -2.429 | 0.9627 | 71 | 3.568 | 0.1669 |
| 590_E5_N4.2 | 1200_D9_N1.1 | 11 | 13 | 5 | 5.231 | -0.2308 | 0.9788 | 71 | 0.3334 | >0.9999 |
| 521_C7_N1.3 | 1200_B8_N1.2 | 10 | 9 | 2.4 | 5.778 | -3.378 | 1.098 | 71 | 4.351 | 0.0447 |
| 521_C7_N1.3 | 1200_D9_N1.2 | 10 | 14 | 2.4 | 7.429 | -5.029 | 0.9893 | 71 | 7.189 | <0.0001 |
| 521_C7_N1.3 | 1200_D9_N1.1 | 10 | 13 | 2.4 | 5.231 | -2.831 | 1.005 | 71 | 3.983 | 0.0862 |
| 1200_B8_N1.2 | 1200_D9_N1.2 | 9 | 14 | 5.778 | 7.429 | -1.651 | 1.021 | 71 | 2.287 | 0.6719 |
| 1200_B8_N1.2 | 1200_D9_N1.1 | 9 | 13 | 5.778 | 5.231 | 0.547 | 1.036 | 71 | 0.7467 | 0.9983 |
| 1200_D9_N1.2 | 1200_D9_N1.1 | 14 | 13 | 7.429 | 5.231 | 2.198 | 0.9203 | 71 | 3.377 | 0.2189 |
| **Nodule dry weight (g)** | | | | | | | | | | |
| G49 | 532C | 10 | 11 | 0.02073 | 0.01422 | 0.006512 | 0.006479 | 71 | 1.421 | 0.9512 |
| G49 | 590_E5_N4.2 | 10 | 11 | 0.02073 | 0.01221 | 0.008521 | 0.006479 | 71 | 1.86 | 0.8426 |
| G49 | 521_C7_N1.3 | 10 | 10 | 0.02073 | 0.00344 | 0.01729 | 0.006631 | 71 | 3.687 | 0.1393 |
| G49 | 1200_B8_N1.2 | 10 | 9 | 0.02073 | 0.01793 | 0.002797 | 0.006813 | 71 | 0.5805 | 0.9996 |
| G49 | 1200_D9_N1.2 | 10 | 14 | 0.02073 | 0.03515 | -0.01442 | 0.006139 | 71 | 3.322 | 0.2361 |
| G49 | 1200_D9_N1.1 | 10 | 13 | 0.02073 | 0.0194 | 0.00133 | 0.006237 | 71 | 0.3016 | >0.9999 |
| 532C | 590_E5_N4.2 | 11 | 11 | 0.01422 | 0.01221 | 0.002009 | 0.006323 | 71 | 0.4494 | >0.9999 |
| 532C | 521_C7_N1.3 | 11 | 10 | 0.01422 | 0.00344 | 0.01078 | 0.006479 | 71 | 2.353 | 0.642 |
| 532C | 1200_B8_N1.2 | 11 | 9 | 0.01422 | 0.01793 | -0.00372 | 0.006665 | 71 | 0.7883 | 0.9978 |
| 532C | 1200_D9_N1.2 | 11 | 14 | 0.01422 | 0.03515 | -0.02093 | 0.005974 | 71 | 4.955 | 0.0134 |
| 532C | 1200_D9_N1.1 | 11 | 13 | 0.01422 | 0.0194 | -0.00518 | 0.006075 | 71 | 1.206 | 0.9781 |
| 590_E5_N4.2 | 521_C7_N1.3 | 11 | 10 | 0.01221 | 0.00344 | 0.008769 | 0.006479 | 71 | 1.914 | 0.824 |
| 590_E5_N4.2 | 1200_B8_N1.2 | 11 | 9 | 0.01221 | 0.01793 | -0.00572 | 0.006665 | 71 | 1.215 | 0.9774 |
| 590_E5_N4.2 | 1200_D9_N1.2 | 11 | 14 | 0.01221 | 0.03515 | -0.02294 | 0.005974 | 71 | 5.43 | 0.0047 |
| 590_E5_N4.2 | 1200_D9_N1.1 | 11 | 13 | 0.01221 | 0.0194 | -0.00719 | 0.006075 | 71 | 1.674 | 0.898 |
| 521_C7_N1.3 | 1200_B8_N1.2 | 10 | 9 | 0.00344 | 0.01793 | -0.01449 | 0.006813 | 71 | 3.008 | 0.3484 |
| 521_C7_N1.3 | 1200_D9_N1.2 | 10 | 14 | 0.00344 | 0.03515 | -0.03171 | 0.006139 | 71 | 7.305 | <0.0001 |
| 521_C7_N1.3 | 1200_D9_N1.1 | 10 | 13 | 0.00344 | 0.0194 | -0.01596 | 0.006237 | 71 | 3.619 | 0.1547 |
| 1200_B8_N1.2 | 1200_D9_N1.2 | 9 | 14 | 0.01793 | 0.03515 | -0.01722 | 0.006335 | 71 | 3.843 | 0.1088 |
| 1200_B8_N1.2 | 1200_D9_N1.1 | 9 | 13 | 0.01793 | 0.0194 | -0.00147 | 0.00643 | 71 | 0.3226 | >0.9999 |
| 1200_D9_N1.2 | 1200_D9_N1.1 | 14 | 13 | 0.03515 | 0.0194 | 0.01575 | 0.005711 | 71 | 3.9 | 0.0991 |

###### **Table S11:** Comparison of yield and nodule traits between strains in the field. The results of the generalized linear mixed model with Tukey's Honestly Significant Difference (HSD) test for pairwise differences are summarised in this table.

| Location | Contrast | Estimate | SE | df | t-ratio | *P* value |
| --- | --- | --- | --- | --- | --- | --- |
| Nodule number | | | | | | |
| Merelbeke | 1200_B8_N1.2 - G49 | 1.556 | 0.630 | 19.8 | 2.471 | 0.0960 |
|  | 1200_B8_N1.2 - Control | 4.761 | 0.630 | 19.8 | 7.561 | <.0001 |
|  | 1200_B8_N1.2 - 590_E5_N4.2 | 0.759 | 0.630 | 19.8 | 1.205 | 0.6312 |
|  | 590_E5_N4.2 - G49 | 0.798 | 0.719 | 13.1 | 1.109 | 0.6907 |
|  | 590_E5_N4.2 - Control | 4.002 | 0.719 | 13.1 | 5.564 | 0.0005 |
|  | G49 - Control | 3.205 | 0.719 | 13.1 | 4.455 | 0.0031 |
| Bottelare | 1200_B8_N1.2 - G49 | 1.149 | 0.278 | 11.0 | 4.125 | 0.0078 |
|  | 1200_B8_N1.2 - Control | 3.178 | 0.278 | 11.0 | 11.413 | <.0001 |
|  | 1200_B8_N1.2 - 590_E5_N4.2 | -0.307 | 0.278 | 11.0 | -1.101 | 0.6963 |
|  | 590_E5_N4.2 - G49 | 1.455 | 0.278 | 14.7 | 5.226 | 0.0006 |
|  | 590_E5_N4.2 - Control | 3.485 | 0.278 | 11.0 | 12.514 | <.0001 |
|  | G49 - Control | 2.030 | 0.278 | 11.0 | 7.288 | <.0001 |
| Nodule dry weight (g) | | | | | | |
| Merelbeke | 1200_B8_N1.2 - G49 | 0.160 | 0.067 | 19.7 | 2.369 | 0.1164 |
|  | 1200_B8_N1.2 - Control | 0.543 | 0.067 | 19.7 | 8.061 | <.0001 |
|  | 1200_B8_N1.2 - 590_E5_N4.2 | 0.068 | 0.067 | 19.7 | 1.008 | 0.7467 |
|  | 590_E5_N4.2 - G49 | 0.092 | 0.077 | 13.0 | 1.196 | 0.6401 |
|  | 590_E5_N4.2 - Control | 0.475 | 0.077 | 13.0 | 6.196 | 0.0002 |
|  | G49 - Control | 0.384 | 0.077 | 13.0 | 5.000 | 0.0012 |
| Bottelare | 1200_B8_N1.2 - G49 | 0.113 | 0.029 | 11.0 | 3.845 | 0.0124 |
|  | 1200_B8_N1.2 - Control | 0.312 | 0.029 | 11.0 | 10.646 | <.0001 |
|  | 1200_B8_N1.2 - 590_E5_N4.2 | -0.058 | 0.029 | 11.0 | -1.993 | 0.2478 |
|  | 590_E5_N4.2 - G49 | 0.171 | 0.029 | 14.7 | 5.839 | 0.0002 |
|  | 590_E5_N4.2 - Control | 0.371 | 0.029 | 11.0 | 12.640 | <.0001 |
|  | G49 - Control | 0.199 | 0.029 | 11..0 | 6.801 | 0.0001 |
| Chlorophyll content | | | | | | |
| Merelbeke | 1200_B8_N1.2 - G49 | 5.966 | 1.305 | 19.5 | 4.572 | 0.0010 |
|  | 1200_B8_N1.2 - Control | 8.293 | 1.305 | 19.5 | 6.356 | <.0001 |
|  | 1200_B8_N1.2 - 590_E5_N4.2 | -0.601 | 1.305 | 19.5 | -0.461 | 0.9667 |
|  | 590_E5_N4.2 - G49 | 6.567 | 1.447 | 12.3 | 4.537 | 0.0031 |
|  | 590_E5_N4.2 - Control | 8.894 | 1.447 | 12.3 | 6.145 | 0.0002 |
|  | G49 - Control | 2.327 | 1.447 | 12.3  3 | 1.608 | 0.4095 |
| Bottelare | 1200_B8_N1.2 - G49 | -1.092 | 1.312 | 9.5 | -0.832 | 0.8382 |
|  | 1200_B8_N1.2 - Control | 1.417 | 1.312 | 9.5 | 1.080 | 0.7094 |
|  | 1200_B8_N1.2 - 590_E5_N4.2 | -3.722 | 1.312 | 9.5 | -2.836 | 0.0741  . |
|  | 590_E5_N4.2 - G49 | 2.630 | 1.197 | 14.4 | 2.197 | 0.1706 |
|  | 590_E5_N4.2 - Control | 5.138 | 1.312 | 9.5 | 3.916 | 0.0141 |
|  | G49 - Control | 2.508 | 1.312 | 9.5 | 1.911 | 0.2859 |
| Yield (t/ha) | | | | | | |
| Merelbeke | 1200_B8_N1.2 - G49 | 151.532 | 171.648 | 19.5 | 0.883 | 0.8137 |
|  | 1200_B8_N1.2 - Control | 524.361 | 171.648 | 19.5 | 3.055  0 | 0.0299 |
|  | 1200_B8_N1.2 - 590_E5_N4.2 | -213.668 | 171.648 | 19.5 | -1.245 | 0.6071 |
|  | 590_E5_N4.2 - G49 | 365.200 | 224.193 | 18.9 | 1.629 | 0.3871 |
|  | 590_E5_N4.2 - Control | 738.030 | 224.193 | 18.9 | 3.292 | 0.0185 |
|  | G49 - Control | 372.830 | 224.193 | 18.9 | 1.663 | 0.3696 |
| Bottelare | 1200_B8_N1.2 - G49 | 65.434 | 199.334 | 6.8 | 0.328 | 0.9867 |
|  | 1200_B8_N1.2 - Control | 203.108 | 199.334 | 6.8 | 1.019 | 0.7447 |
|  | 1200_B8_N1.2 - 590_E5_N4.2 | 113.111 | 199.334 | 6.8 | 0.570 | 0.9379 |
|  | 590_E5_N4.2 - G49 | -48.147 | 113.111 | 14.0 | -0.426 | 0.9731 |
|  | 590_E5_N4.2 - Control | 89.527 | 199.334 | 6.8 | 0.449 | 0.9677 |
|  | G49 - Control | 137.675 | 199.334 | 6.8 | 0.691 | 0.8974 |
| Protein content (%) | | | | | | |
| Merelbeke | 1200_B8_N1.2 - G49 | 1.541 | 0.964 | 19.9 | 1.598 | 0.4022 |
|  | 1200_B8_N1.2 - Control | 4.436 | 0.964 | 19.9 | 4.601 | 0.0009 |
|  | 1200_B8_N1.2 - 590_E5_N4.2 | -0.767 | 0.964 | 19.9 | -0.796 | 0.8556 |
|  | 590_E5_N4.2 - G49 | 2.308 | 1.219 | 17.1 | 1.892 | 0.2675 |
|  | 590_E5_N4.2 - Control | 5.203 | 1.219 | 17.1 | 4.266 | 0.0026 |
|  | G49 - Control | 2.895 | 1.219 | 17.1 | 2.374 | 0.1201 |
| Bottelare | 1200_B8_N1.2 - G49 | -0.803 | 1.206 | 6.7 | -0.666 | 0.9064 |
|  | 1200_B8_N1.2 - Control | 1.332 | 1.206 | 6.7 | 1.104 | 0.6989 |
|  | 1200_B8_N1.2 - 590_E5_N4.2 | -2.387 | 1.206 | 6.7 | -1.979 | 0.2835 |
|  | 590_E5_N4.2 - G49 | 1.583 | 0.638 | 14.0 | 2.481 | 0.1067 |
|  | 590_E5_N4.2 - Control | 3.718 | 1.206 | 6.7 | 3.084 | 0.0700 |
|  | G49 - Control | 2.135 | 1.206 | 6.7 | 1.771 | 0.3625 |
| Thousand kernel weight (g) | | | | | | |
| Merelbeke | 1200_B8_N1.2 - G49 | 10.670 | 12.415 | 17.9 | 0.859 | 0.8254 |
|  | 1200_B8_N1.2 - Control | 16.973 | 13.231 | 16.0 | 1.283 | 05862 |
|  | 1200_B8_N1.2 - 590_E5_N4.2 | -2.484 | 12.415 | 17.9 | -0.200 | 0.9971 |
|  | 590_E5_N4.2 - G49 | 13.154 | 13.062 | 10.8 | 1.007 | 0.7489 |
|  | 590_E5_N4.2 - Control | 19.457 | 13.454 | 10.7 | 1.446 | 0.4996 |
|  | G49 - Control | 6.304 | 13.454 | 10.7 | 0.469 | 0.9644 |
| Bottelare | 1200_B8_N1.2 - G49 | -9.057 | 11.305 | 10.8 | -0.801 | 0.8525 |
|  | 1200_B8_N1.2 - Control | 4.632 | 10.616 | 9.9 | 0.436 | 0.9708 |
|  | 1200_B8_N1.2 - 590_E5_N4.2 | -14.174 | 10.616 | 9.9 | -1.335 | 0.5633 |
|  | 590_E5_N4.2 - G49 | 5.117 | 11.305 | 14.2 | 0.453 | 0.9680 |
|  | 590_E5_N4.2 - Control | 18.806 | 10.616 | 9.9 | 1.771 | 0.3406 |
|  | G49 - Control | 13.689 | 11.305 | 10.8 | 1.211 | 0.6332 |

###### Table S12: Position, size, and orientation of the genes selected for presence/absence screening

|  | Bradyrhizobium 1200_B8_1.2 | | | | Bradyrhizobium 1200_D9_N1.1 | | | | Bradyrhizobium 1200_D9_N1.2 | | | | Bradyrhizobium 521_C7_N1.3 | | | | Bradyrhizobium 590_E5_N4.2 | | | |
| --- | --- | --- | --- | --- | --- | --- | --- | --- | --- | --- | --- | --- | --- | --- | --- | --- | --- | --- | --- | --- |
| Genes | **Start** | **End** | **Strand** | **Length** | **Start** | **End** | **Strand** | **Length** | **Start** | **End** | **Strand** | **Length** | **Start** | **End** | **Strand** | **Length** | **Start** | **End** | **Strand** | **Length** |
| *fixA* | 7865417 | 7866283 | - | 867 | 7865319 | 7866185 | - | 867 | 7788500 | 7789366 | - | 867 | 2271580 | 2272446 | + | 867 | 7786936 | 7787802 | - | 867 |
| *nifA* | 7866801 | 7868618 | - | 1818 | 7866703 | 7868520 | - | 1818 | 7789884 | 7791701 | - | 1818 | 2269245 | 2271062 | + | 1818 | 7788320 | 7790137 | - | 1818 |
| *nolO* | 7871645 | 7873252 | - | 1608 | 7875543 | 7877252 | - | 1710 | 7794728 | 7796335 | - | 1608 | 2264611 | 2266218 | + | 1608 | 7793164 | 7794771 | - | 1608 |
| *nolN* | 7873288 | 7873671 | - | 384 | 7873190 | 7873573 | - | 384 | 7796371 | 7796754 | - | 384 | 2264192 | 2264575 | + | 384 | 7794807 | 7795190 | - | 384 |
| *nodJ* | 7873927 | 7874715 | - | 789 | 7873829 | 7874617 | - | 789 | 7797010 | 7797798 | - | 789 | 2263148 | 2263936 | + | 789 | 7795446 | 7796234 | - | 789 |
| *nodI* | 7874719 | 7875639 | - | 921 | 7874621 | 7875541 | - | 921 | 7797802 | 7798722 | - | 921 | 2262224 | 2263144 | + | 921 | 7796238 | 7797158 | - | 921 |
| *nodU* | 7875641 | 7877350 | - | 1710 | 7875543 | 7877252 | - | 1710 | 7798724 | 7800433 | - | 1710 | 2260513 | 2262222 | + | 1710 | 7797160 | 7798869 | - | 1710 |
| *nodC* | 7877920 | 7879377 | - | 1458 | 7877822 | 7879279 | - | 1458 | 7801003 | 7802460 | - | 1458 | 2258486 | 2259943 | + | 1458 | 7799439 | 7800896 | - | 1458 |
| *nodB* | 7879392 | 7880051 | - | 660 | 7879294 | 7879953 | - | 660 | 7802475 | 7803134 | - | 660 | 2257812 | 2258471 | + | 660 | 7800911 | 7801570 | - | 660 |
| *nodA* | 7880048 | 7880680 | - | 633 | 7879950 | 7880582 | - | 633 | 7803131 | 7803763 | - | 633 | 2257183 | 2257815 | + | 633 | 7801567 | 7802199 | - | 633 |
| *nodD1* | 7881474 | 7882418 | + | 945 | 7881376 | 7882320 | + | 945 | 7804557 | 7805501 | + | 945 | 2255445 | 2256389 | - | 945 | 7802993 | 7803937 | + | 945 |
| *nodD2* | 7883052 | 7884044 | + | 993 | 7882954 | 7883946 | + | 993 | 7806135 | 7807127 | + | 993 | 2253819 | 2254811 | - | 993 | 7804571 | 7805563 | + | 993 |
| *nolY* | 7888154 | 7888882 | + | 729 | 7888056 | 7888784 | + | 729 | 7811654 | 7812016 | + | 363 | 2248931 | 2249293 | - | 363 | 7810090 | 7810452 | + | 363 |
| *rhcU* | 8096822 | 8097859 | - | 1038 | 8096724 | 8097761 | - | 1038 | 8035311 | 8036348 | - | 1038 | 1659602 | 1660639 | + | 1038 | 8033742 | 8034779 | - | 1038 |
| *rhcT* | 8097856 | 8098677 | - | 822 | 8097758 | 8098579 | - | 822 | 8036345 | 8037166 | - | 822 | 1658784 | 1659605 | + | 822 | 8034776 | 8035597 | - | 822 |
| *rhcS* | 8098688 | 8098963 | - | 276 | 8098590 | 8098865 | - | 276 | 8037177 | 8037452 | - | 276 | 1658498 | 1658773 | + | 276 | 8035608 | 8035883 | - | 276 |
| *rhcR* | 8098966 | 8099631 | - | 666 | 8098868 | 8099533 | - | 666 | 8037455 | 8038120 | - | 666 | 1657830 | 1658495 | + | 666 | 8035886 | 8036551 | - | 666 |
| *rhcQ* | 8099624 | 8100745 | - | 1122 | 8099526 | 8100647 | - | 1122 | 8038113 | 8039234 | - | 1122 | 1656716 | 1657837 | + | 1122 | 8036544 | 8037665 | - | 1122 |
| *rhcN* | 8101254 | 8102609 | - | 1356 | 8101156 | 8102511 | - | 1356 | 8039743 | 8041098 | - | 1356 | 1654852 | 1656207 | + | 1356 | 8038174 | 8039529 | - | 1356 |
| *nolV* | 8102606 | 8103226 | - | 621 | 8102508 | 8103128 | - | 621 | 8041095 | 8041715 | - | 621 | 1654235 | 1654855 | + | 621 | 8039526 | 8040146 | - | 621 |
| *nolU* | 8103223 | 8103861 | - | 639 | 8103125 | 8103763 | - | 639 | 8041712 | 8042350 | - | 639 | 1653600 | 1654238 | + | 639 | 8040143 | 8040781 | - | 639 |
| *rhcJ* | 8103873 | 8104736 | - | 864 | 8103775 | 8104578 | - | 804 | 8042362 | 8043225 | - | 864 | 1652725 | 1653588 | + | 864 | 8040793 | 8041656 | - | 864 |
| *nopB* | 8104745 | 8104918 | - | 174 | 8104647 | 8104820 | - | 174 | 8043234 | 8043407 | - | 174 | 1652543 | 1652716 | + | 174 | 8041665 | 8041838 | - | 174 |
| *rhcC1* | 8105428 | 8106120 | + | 693 | 8105330 | 8106022 | + | 693 | 8043917 | 8044609 | + | 693 | 1651341 | 1652033 | - | 693 | 8042348 | 8043040 | + | 693 |
| *nopA* | 8113506 | 8113698 | + | 192 | 8113408 | 8113600 | + | 192 | 8052091 | 8052283 | + | 192 | 1645293 | 1645485 | - | 192 | 8048896 | 8049088 | + | 192 |
| *rhcV* | 8115719 | 8117818 | + | 2100 | 8115621 | 8117720 | + | 2100 | 8054304 | 8056403 | + | 2100 | 1641174 | 1643273 | - | 2100 | 8051109 | 8053208 | + | 2100 |
| *fixX* | 8137459 | 8137755 | - | 297 | 8137361 | 8137657 | - | 297 | 8073678 | 8073974 | - | 297 | 1623617 | 1623913 | + | 297 | 8070483 | 8070779 | - | 297 |
| *fixC* | 8137794 | 8139101 | - | 1308 | 8137696 | 8139003 | - | 1308 | 8074013 | 8075320 | - | 1308 | 1622271 | 1623578 | + | 1308 | 8070818 | 8072125 | - | 1308 |
| *fixB* | 8139113 | 8140222 | - | 1110 | 8139015 | 8140124 | - | 1110 | 8075332 | 8076441 | - | 1110 | 1621150 | 1622259 | + | 1110 | 8072137 | 8073246 | - | 1110 |
| *nifW* | 8141173 | 8141514 | - | 342 | 8141075 | 8141416 | - | 342 | 8077392 | 8077733 | - | 342 | 1619858 | 1620199 | + | 342 | 8074197 | 8074538 | - | 342 |
| *nifQ* | 8142169 | 8142945 | - | 777 | 8142071 | 8142847 | - | 777 | 8078387 | 8079163 | - | 777 | 1618427 | 1619203 | + | 777 | 8075192 | 8075968 | - | 777 |
| *nifH* | 8143092 | 8143976 | - | 885 | 8142994 | 8143878 | - | 885 | 8079309 | 8080193 | - | 885 | 1617396 | 1618280 | + | 885 | 8076114 | 8076998 | - | 885 |
| *nifO* | 8144544 | 8144969 | + | 426 | 8144446 | 8144871 | + | 426 | 8080761 | 8081186 | + | 426 | 1616403 | 1616828 | - | 426 | 8077566 | 8077991 | + | 426 |
| *nifZ* | 8148385 | 8148699 | - | 315 | 8148287 | 8148601 | - | 315 | 8084602 | 8084916 | - | 315 | 1612673 | 1612987 | + | 315 | 8081407 | 8081721 | - | 315 |
| *nifB* | 8149185 | 8150768 | - | 1584 | 8149087 | 8150670 | - | 1584 | 8085402 | 8086985 | - | 1584 | 1610604 | 1612187 | + | 1584 | 8082207 | 8083790 | - | 1584 |
| *nifT* | 8151432 | 8151656 | - | 225 | 8151334 | 8151558 | - | 225 | 8087649 | 8087873 | - | 225 | 1609716 | 1609940 | + | 225 | 8084454 | 8084678 | - | 225 |
| *nifS* | 8151653 | 8152834 | - | 1182 | 8151555 | 8152736 | - | 1182 | 8087870 | 8089051 | - | 1182 | 1607894 | 1608214 | + | 321 | 8084675 | 8085856 | - | 1182 |
| *blr1175* | 8153158 | 8153478 | - | 321 | 8153060 | 8153380 | - | 321 | 8089375 | 8089695 | - | 321 | 1608538 | 1609719 | + | 1182 | 8086180 | 8086500 | - | 321 |
| *fer3* | 8157058 | 8157351 | - | 294 | 8156960 | 8157253 | - | 294 | 8093276 | 8093569 | - | 294 | 1604021 | 1604314 | + | 294 | 8090081 | 8090374 | - | 294 |
| *Blr1748* | 8157576 | 8158040 | - | 465 | 8157478 | 8157942 | - | 465 | 8093794 | 8094168 | - | 375 | 1603332 | 1603796 | + | 465 | 8090599 | 8090973 | - | 375 |
| *nifX* | 8158047 | 8158442 | - | 396 | 8157949 | 8158344 | - | 396 | 8094263 | 8094658 | - | 396 | 1602930 | 1603325 | + | 396 | 8091068 | 8091463 | - | 396 |
| *nifN* | 8158439 | 8159848 | - | 1410 | 8158341 | 8159750 | - | 1410 | 8094655 | 8096064 | - | 1410 | 1601524 | 1602933 | + | 1410 | 8091460 | 8092869 | - | 1410 |
| *nifE* | 8159858 | 8161501 | - | 1644 | 8159760 | 8161403 | - | 1644 | 8096074 | 8097717 | - | 1644 | 1599871 | 1601514 | + | 1644 | 8092879 | 8094522 | - | 1644 |
| *nifK* | 8161594 | 8163150 | - | 1557 | 8161496 | 8163052 | - | 1557 | 8097810 | 8099366 | - | 1557 | 1598222 | 1599778 | + | 1557 | 8094615 | 8096171 | - | 1557 |
| *nifD* | 8163216 | 8164718 | - | 1503 | 8163118 | 8164620 | - | 1503 | 8099432 | 8100934 | - | 1503 | 1596654 | 1598156 | + | 1503 | 8096237 | 8097739 | - | 1503 |
| *Bsr1739* | 8167738 | 8167932 | - | 195 | 8167640 | 8167834 | - | 195 | 8103954 | 8104148 | - | 195 | 1593481 | 1593675 | + | 195 | 8100759 | 8100953 | - | 195 |
| *ybgC* | 2014802 | 2015245 | + | 444 | 2014782 | 2015225 | + | 444 | 1897294 | 1897737 | + | 444 | 8985850 | 8986293 | - | 444 | 1897366 | 1897809 | + | 444 |

###### Table S13: ANI values, representing the genetic similarity of the isolated strains to the respective type strains. Isolated strains tested in the field are highlighted in bold and red.

| **Isolated strain** | **Type strain** | **ANI** |
| --- | --- | --- |
| *Bradyrhizobium* sp. 1050_B9_N1.2 | *Bradyrhizobium baranii* subsp. *barranii* 144S4 | 97.07 |
| ***Bradyrhizobium* sp. 1200_B8_N1.2** | *Bradyrhizobium diazoefficiens* USDA110 | 99.68 |
|  | *Bradyrhizobium diazoefficiens* G49 | 98.72 |
| *Bradyrhizobium* sp. 1200_D9_N1.1 | *Bradyrhizobium diazoefficiens* USDA110 | 99.67 |
|  | *Bradyrhizobium diazoefficiens G49* | 98.69 |
| *Bradyrhizobium* sp. 1200_D9_N1.2 | *Bradyrhizobium diazoefficiens* USDA110 | 98.70 |
|  | *Bradyrhizobium diazoefficiens* G49 | 99.98 |
| *Bradyrhizobium* sp. 215_C5_N1.1 | *Bradyrhizobium baranii* subsp. *barranii* 144S4 | 97.13 |
| *Bradyrhizobium* sp. 215_C5_N1.2 | *Bradyrhizobium baranii* subsp. *barranii* 144S4 | 97.16 |
| *Bradyrhizobium* sp. 482_C4_N1.1 | *Bradyrhizobium baranii* subsp. *barranii* 144S4 | 97.20 |
| *Bradyrhizobium* sp. 482_C4_N1.8 | *Bradyrhizobium baranii* subsp. *barranii* 144S4 | 97.18 |
| *Bradyrhizobium* sp. 52_B10_N1.2 | *Bradyrhizobium baranii* subsp. *barranii* 144S4 | 97.03 |
| *Bradyrhizobium* sp. 521_C7_N1.3 | *Bradyrhizobium japonicum* USDA6 | 96.92 |
|  | *Bradyrhizobium japonicum* 532C | 96.96 |
| ***Bradyrhizobium* sp. 590_E5_N4.2** | *Bradyrhizobium diazoefficiens* USDA110 | 98.73 |
|  | *Bradyrhizobium diazoefficiens* G49 | 99.97 |
| *Bradyrhizobium* sp. 613_E4_N2.2 | *Bradyrhizobium baranii* subsp. *barranii* 144S4 | 97.22 |
| *Bradyrhizobium* sp. 63_E2_N1.1 | *Bradyrhizobium baranii* subsp. *barranii* 144S4 | 97.13 |
| *Bradyrhizobium* sp. 63_E2_N1.3 | *Bradyrhizobium baranii* subsp. *barranii* 144S4 | 97.07 |
| *Bradyrhizobium* sp. 930_D9_N1.4 | *Bradyrhizobium* *xenonodulans* 14ABT | 88.39 |
| *Bradyrhizobium* sp. 930_D9_N1.4 | *Bradyrhizobium baranii* subsp. *barranii* 144S4 | 88.94 |
| *Bradyrhizobium* sp. 956_D2_N1.4 | *Bradyrhizobium baranii* subsp. *barranii* 144S4 | 97.20 |
| *Bradyrhizobium* sp. 956_D2_N1.5 | *Bradyrhizobium baranii* subsp. *barranii* 144S4 | 97.18 |
| *Rhizobium* sp. 11_C7_N12.5 | *Rhizobium lusitanum* P1-7 | 99.09 |
| *Rhizobium* sp. 32_C3_N1.1 | *Rhizobium viscosum* DSM7307 | 91.43 |
| *Rhizobium* sp. 62_C5_N11.2 | *Rhizobium leguminosarum* USDA 2370 | 98.39 |
| *Rhizobium* sp. 768_B6_N1.8 | *Rhizobium viscosum* DSM7307 | 91.44 |
| *Rhizobium* sp. 814_E9_N1.1 | *Rhizobium leguminosarum* USDA 2370 | 98.18 |
| *Rhizobium* sp. 862_C5_N1.2 | *Rhizobium leguminosarum* USDA 2370 | 98.16 |
| *Rhizobium* sp. 969_B3_N1.2 | *Rhizobium redzepovicii* 18T | 99.59 |
| *Tardiphaga* sp. 1201_B9_N1.1 | *Tardiphaga robiniae* LMG 26467 | 96.51 |
| *Tardiphaga* sp. 1201_B9_N1.2 | *Tardiphaga robiniae* LMG 26467 | 96.50 |
| *Tardiphaga* sp. 172_B4_N1.3 | *Tardiphaga robiniae* LMG 26467 | 96.20 |
| *Tardiphaga* sp. 20_F10_N6.6 | *Tardiphaga robiniae* LMG 26467 | 96.37 |
| *Tardiphaga* sp. 215_C5_N2.1 | *Tardiphaga robiniae* LMG 26467 | 96.38 |
| *Tardiphaga* sp. 285_C5_N1.2 | *Tardiphaga robiniae* LMG 26467 | 96.54 |
| *Tardiphaga* sp. 367_B4_N1.1 | *Tardiphaga robiniae* LMG 26467 | 96.50 |
| *Tardiphaga* sp. 538_B7_N1.4 | *Tardiphaga robiniae* LMG 26467 | 96.49 |
| *Tardiphaga* sp. 619_E2_N8.5 | *Tardiphaga robiniae* LMG 26467 | 96.51 |
| *Tardiphaga* sp. 619_E2_N8.6 | *Tardiphaga robiniae* LMG 26467 | 96.52 |
| *Tardiphaga* sp. 768_D3_N2.1 | *Tardiphaga robiniae* LMG 26467 | 91.14 |
| *Tardiphaga* sp. 803_E3_N1.3 | *Tardiphaga robiniae* LMG 26467 | 96.51 |
| *Tardiphaga* sp. 804_B3_N1.9 | *Tardiphaga robiniae* LMG 26467 | 99.87 |
| *Tardiphaga* sp. 813_E8_N1.3 | *Tardiphaga robiniae* LMG 26467 | 93.17 |
| *Tardiphaga* sp. 839_C3_N1.4 | *Tardiphaga robiniae* LMG 26467 | 94.16 |
| *Tardiphaga* sp. 841_E9_N1.2 | *Tardiphaga robiniae* LMG 26467 | 96.28 |
| *Tardiphaga* sp. 862_B3_N1.1 | *Tardiphaga robiniae* LMG 26467 | 96.53 |
| *Tardiphaga* sp. 862_B3_N4.1 | *Tardiphaga robiniae* LMG 26467 | 96.39 |
| *Tardiphaga* sp. 866_E4_N1.4 | *Tardiphaga robiniae* LMG 26467 | 95.93 |
| *Tardiphaga* sp. 866_E4_N2.3 | *Tardiphaga robiniae* LMG 26467 | 95.91 |
| *Tardiphaga* sp. 94_D9_N1.1 | *Tardiphaga robiniae* LMG 26467 | 96.46 |

###### **Table S14:** Bioproject accession numbers for the respective datasets. Strain names denote the full genome sequence of the respective strain.

| **Dataset** | **Bioproject accession number** |
| --- | --- |
| Microbiome nodules and soil 16S rRNA and ITS | PRJNA1141995 |
| *Bradyrhizobium* sp. 63_E2_N1_1 | PRJNA1143383 |
| *Bradyrhizobium* sp. 63_E2_N1_3 | PRJNA1143385 |
| *Bradyrhizobium* sp. 215_C5_N1_1 | PRJNA1142925 |
| *Bradyrhizobium* sp. 215_C5_N1_2 | PRJNA1143191 |
| *Bradyrhizobium* sp. 482_C4_N1_1 | PRJNA1143192 |
| *Bradyrhizobium* sp. 482_C4_N1_8 | PRJNA1143194 |
| *Bradyrhizobium* sp. 521_C7_N1_3 | PRJNA1143196 |
| *Bradyrhizobium* sp. 590_E5_N4_2 | PRJNA1143200 |
| *Bradyrhizobium* sp. 604_D8_N2_3 | PRJNA1143204 |
| *Bradyrhizobium* sp. 613_E4_N2_2 | PRJNA1143206 |
| *Bradyrhizobium* sp. 930_D9_N1_4 | PRJNA1143387 |
| *Bradyrhizobium* sp. 956_D2_N1_4 | PRJNA1143390 |
| *Bradyrhizobium* sp. 956_D2_N1_5 | PRJNA1143391 |
| *Bradyrhizobium* sp. 1050_B9_N1_2 | PRJNA1140108 |
| *Bradyrhizobium* sp. 1200_B8_N1_2 | PRJNA1140550 |
| *Bradyrhizobium* sp. 1200_D9_N1_1 | PRJNA1140556 |
| *Bradyrhizobium* sp. 1200_D9_N1_2 | PRJNA1140558 |
| *Rhizobium* sp. 11_C7_N12_5 | PRJNA1143490 |
| *Rhizobium* sp. 32_C3_N1_1 | PRJNA1143491 |
| *Rhizobium* sp. 62_C5_N11_2 | PRJNA1143492 |
| *Rhizobium* sp. 768_B6_N1_8 | PRJNA1143494 |
| *Rhizobium* sp. 814_E9_N1_1 | PRJNA1150975 |
| *Rhizobium* sp. 862_C5_N1_2 | PRJNA1150976 |
| *Rhizobium* sp. 969_B3_N1_2 | PRJNA1150977 |
| *Tardiphaga* sp. 11_C7_N12_6 | PRJNA1143415 |
| *Tardiphaga* sp. 20_F10_N6_6 | PRJNA1143427 |
| *Tardiphaga* sp. 71_E8_N1_1 | PRJNA1143467 |
| *Tardiphaga* sp. 94_D9_N1_1 | PRJNA1143489 |
| *Tardiphaga* sp. 172_B4_N1_3 | PRJNA1143425 |
| *Tardiphaga* sp. 215_C5_N2_1 | PRJNA1143429 |
| *Tardiphaga* sp. 285_C5_N1_2 | PRJNA1143443 |
| *Tardiphaga* sp. 367_B4_N1_1 | PRJNA1143451 |
| *Tardiphaga* sp. 538_B7_N1_4 | PRJNA1143452 |
| *Tardiphaga* sp. 604_B6_N1_1 | PRJNA1143455 |
| *Tardiphaga* sp. 619_E2_N8_4 | PRJNA1143456 |
| *Tardiphaga* sp. 619_E2_N8_5 | PRJNA1143464 |
| *Tardiphaga* sp. 619_E2_N8_6 | PRJNA1143465 |
| *Tardiphaga* sp. 768_D3_N2_1 | PRJNA1143466 |
| *Tardiphaga* sp. 803_E3_N1_3 | PRJNA1143468 |
| *Tardiphaga* sp. 804_B3_N1_9 | PRJNA1143470 |
| *Tardiphaga* sp. 813_E8_N1_3 | PRJNA1143473 |
| *Tardiphaga* sp. 839_C3_N1_4 | PRJNA1143474 |
| *Tardiphaga* sp. 841_E9_N1_2 | PRJNA1143475 |
| *Tardiphaga* sp. 862_B3_N1_1 | PRJNA1143477 |
| *Tardiphaga* sp. 862_B3_N4_1 | PRJNA1143478 |
| *Tardiphaga* sp. 866_E4_N1_4 | PRJNA1143480 |
| *Tardiphaga* sp. 866_E4_N2_1 | PRJNA1143482 |
| *Tardiphaga* sp. 866_E4_N2_3 | PRJNA1143487 |
| *Tardiphaga* sp. 1201_B9_N1_1 | PRJNA1143410 |
| *Tardiphaga* sp. 1201_B9_N1_2 | PRJNA1143413 |

###### **Table S15:** Tuning parameters of the multi-variable nodule presence models.

| **models** | **eln-log** | | **pls-log** | | **RF** | | | |
| --- | --- | --- | --- | --- | --- | --- | --- | --- |
|  | **alpha** | **lambda** | **comp** | **Sign comp** | **ntree** | **mtry** | **Samp-size** | **Node-size** |
| PSC | 0.47 | 4.55 e-5 | 5 | 1-3 | 101 | 1 | 90 | 9 |
| PLFA | 0.19 | 0.71 | 2 | 1-2 | 101 | 16 | 10 | 61 |
| ITS | 0 | 12.81 | 2 | 1-2 | 101 | 71 | 470 | 81 |
| 16S | 0.17 | 0.32 | 11 | 1-11* | 101 | 257 | 30 | 41 |
| PSC+div | 0.01 | 0.14 | 6 | 1-2 | 101 | 16 | 110 | 49 |
| PSC+PLFA+div | 0.27 | 0.05 | 2 | 1-2 | 101 | 4 | 150 | 49 |
| PSC+PLFA+  ITS+div | 0 | 64.8 | 1 | 1 | 101 | 215 | 290 | 21 |
| All+div | 0.01 | 4.84 | 1 | 1 | 1001 | 1456 | 10 | 16 |

* model not better than random

###### **Table S16:** Comparison of Shannon index values between nodule colour groups. The results of the linear mixed model with Tukey's multiple testing correction for pairwise differences are summarised in this table. ‘Estimate’ denotes the estimated difference in Shannon index values between the two groups, with an estimate larger than 0 indicating more diversity in group 1 compared to group 2, and vice versa.

| **Contrast** | **Estimate** | **SE** | **df** | **t-ratio** | ***P* value** |
| --- | --- | --- | --- | --- | --- |
| Brown-Red | 2.5 | 0.17 | 988 | 14.67 | <0.0001 |
| Brown-Unknown | -0.45 | 9.27e^-2^ | 970 | -4.83 | <0.0001 |
| Brown-White | 0.27 | 0.11 | 966 | 2.37 | 0.083 |
| Red-Unkown | -2.95 | 0.15 | 992 | -19.58 | <0.0001 |
| Red-White | -2.23 | 0.16 | 979 | -13.58 | <0.0001 |
| Unknown-White | 0.72 | 8.07e^-2^ | 976 | 8.89 | <0.0001 |

# References

1. Vandecasteele B, Van Loo K, Ommeslag S *et al.* Sustainable Growing Media Blends with Woody Green Composts: Optimizing the N Release with Organic Fertilizers and Interaction with Microbial Biomass. *Agronomy* 2022;**12**: 422.

2. R Core Team. R: A Language and Environment for Statistical Computing. *R Found Stat Comput* *Vienna, Austria* 2022 (http://www.R-project.org/).

3. Pebesma E. Simple features for R: standardized support for spatial vector data. *R J* 2018;**10**:439–46.

4. Tennekes M. tmap: Thematic maps in R. *J Stat Softw* 2018;**84**:1–39.

5. Lahti L, Shetty S. “microbiome R package.” 2012-2019 (DOI: 10.18129/B9.bioc.microbiome).

6. McMurdie PJ, Holmes S. phyloseq: An R Package for reproducible interactive analysis and graphics of microbiome census data. *PLoS One* 2013;**8**:e61217.

7. Bivand R. R packages for analyzing spatial data: a comparative case study with areal data. *Geogr Anal* 2022;**54**:488–518.

8. Benjamini Y, Hochberg Y. Controlling the false discovery rate: a practical and powerful approach to multiple testing. *J R Stat Soc Ser B* 1995;**57**:289–300.

9. Friedman JH, Hastie T, Tibshirani R. Regularization paths for generalized linear models via coordinate descent. *J Stat Softw* 2010;**33**:1–22.

10. Kuhn M. Building predictive models in R using the caret Package. *J Stat Softw* 2008;**28**:1–26.

11. Bischl B, Lang M, Kotthoff L *et al.* mlr: machine learning in R. *J Mach Learn Res* 2016;**17**:170.

12. Airola A, Pahikkala T, Waegeman W *et al.* An experimental comparison of cross-validation techniques for estimating the area under the ROC curve. *Comput Stat Data Anal* 2011;**55**:1828–44.

13. Canty A, Ripley BD. boot: Bootstrap R (S-Plus) Functions. R Package Version 1.3-28.1. 2022.

14. Debeer D, Strobl C. Conditional permutation importance revisited. *BMC Bioinformatics* 2020;**21**:307.

15. Dumolin C, Aerts M, Verheyde B *et al.* Introducing SPeDE: high-throughput dereplication and accurate determination of microbial diversity from matrix-assisted laser desorption–ionization time of flight mass spectrometry data. *mSystems* 2019;**4**:10.1128/msystems.00437-19.

16. Beirinckx S, Viaene T, Haegeman A *et al.* Tapping into the maize root microbiome to identify bacteria that promote growth under chilling conditions. *Microbiome* 2020;**8**:54.

17. Joos L, Beirinckx S, Haegeman A *et al.* Daring to be differential: metabarcoding analysis of soil and plant-related microbial communities using amplicon sequence variants and operational taxonomical units. *BMC Genomics* 2020;**21**:733.

18. Quast C, Pruesse E, Yilmaz P *et al.* The SILVA ribosomal RNA gene database project: improved data processing and web-based tools. *Nucleic Acids Res* 2013;**41**:D590–6.

19. Yilmaz P, Parfrey LW, Yarza P *et al.* The SILVA and “All-species Living Tree Project (LTP)” taxonomic frameworks. *Nucleic Acids Res* 2014;**42**:D643–8.

20. De Tender CA, Debode J, Vandecasteele B *et al.* Biological, physicochemical and plant health responses in lettuce and strawberry in soil or peat amended with biochar. *Appl Soil Ecol* 2016;**107**:1–12.

21. Martin M. Cutadapt removes adapter sequences from high-throughput sequencing reads. *EMBnet.journal* 2011;**17**:10–2.

22. Callahan BJ, McMurdie PJ, Rosen MJ *et al.* DADA2: High-resolution sample inference from Illumina amplicon data. *Nat Methods* 2016;**13**:581–3.

23. Morgan M, Anders S, Lawrence M *et al.* ShortRead: a bioconductor package for input, quality assessment and exploration of high-throughput sequence data. *Bioinformatics* 2009;**25**:2607–8.

24. Pagès H, Aboyoun P, Gentleman R *et al.* Biostrings: Efficient manipulation of biological strings. R package version 2.70.2. 2024.

25. R Core Team. R: A Language and Environment for Statistical Computing. *R Found Stat Comput* *Vienna, Austria* 2019 (http://www.R-project.org/.

26. Sasada R, Weinstein M, Prem A *et al.* FIGARO: An efficient and objective tool for optimizing microbiome rRNA gene trimming parameters. *J Biomol Tech* 2020;**31**:S2.

27. Abarenkov K, Zirk A, Piirmann T *et al.* *UNITE General FASTA Release for Eukaryotes*., 2022.

28. Oksanen J, Simpson G, Blanchet F *et al.* vegan: Community Ecology Package. R package version 2.6-4. 2022.

29. Xu S, Zhan L, Tang W *et al.* MicrobiotaProcess: A comprehensive R package for deep mining microbiome. *Innov* 2023;**4**:100388.

30. Bates D, Mächler M, Bolker B *et al.* Fitting linear mixed-effects models using lme4. *J Stat Softw* 2015;**67**:1–48.

31. Lenth R V., Singmann H, Love J *et al.* emmeans: Estimated marginal means, aka least-squares means (Version 1.3. 4). 2019.

32. Songwattana P, Noisangiam R, Teamtisong K *et al.* Type 3 secretion system (T3SS) of *Bradyrhizobium* sp. DOA9 and its roles in legume symbiosis and rice endophytic association. *Front Microbiol* 2017;**8**: 1810.

33. UniProt Consortium T. UniProt: the universal protein knowledgebase. *Nucleic Acids Res* 2018;**46**:2699.

34. van der Velden N. geneviewer: gene cluster visualizations. R package version 0.1.10. 2025, DOI: 10.32614/CRAN.package.geneviewer.

35. Pannecoucque J, Goormachtigh S, Ceusters N *et al.* Soybean response and profitability upon inoculation and nitrogen fertilisation in Belgium. *Eur J Agron* 2022;**132**:126390.

36. Michiels J, Moris M, Dombrecht B *et al.* Differential regulation of *Rhizobium etli rpoN2* gene expression during symbiosis and free-living growth. *J Bacteriol* 1998;**180**:3620–8.

37. Tits M, Elsen A, Deckers S *et al.* *Bodemvruchtbaarheid van de Akkerbouw- En Weilandpercelen in België En Noordelijk Frankrijk*. Uitgaven van de Bodemkundige Dienst van België, 2020.
